# Air Purifier Intervention for Respiratory Viral Exposure in Elementary Schools: A Secondary Analysis of a Randomized Clinical Trial

**DOI:** 10.1001/jamanetworkopen.2025.36951

**Published:** 2025-10-10

**Authors:** Ye Sun, Dastan Haghnazari, Ching-Ying Huang, Aribah Baig, Minsik Kim, Amparito Cunningham, Colin Skeen, Jack M. Wolfson, Stephen T. Ferguson, Erica D. Pratt, Linda Valeri, Sophia Zhao, Diane R. Gold, Leonora Balaj, Petros Koutrakis, Wanda Phipatanakul, Peggy S. Lai

**Affiliations:** 1 Division of Pulmonary Medicine, Boston Children’s Hospital, Boston, Massachusetts; 2 Division of Pulmonary and Critical Care, Massachusetts General Hospital, Boston; 3 Department of Biological Engineering, Inha University, Incheon, Republic of Korea; 4 Division of Allergy and Immunology, Boston Children’s Hospital, Boston, Massachusetts; 5 Department of Biomedical Engineering, Boston University, Boston, Massachusetts; 6 Department of Environmental Health, Harvard T.H. Chan School of Public Health, Boston, Massachusetts; 7 Department of Biostatistics, Columbia Mailman School of Public Health, New York, New York; 8 Channing Division of Network Medicine, Brigham and Women’s Hospital, Boston, Massachusetts; 9 Department of Neurosurgery, Massachusetts General Hospital, Boston

## Abstract

**Importance:**

The magnitude of school respiratory virus exposure and the effectiveness of environmental mitigation measures remain unclear.

**Objective:**

To evaluate whether portable high-efficiency particulate air (HEPA) purifiers are associated with reduced respiratory virus exposure in elementary school classrooms.

**Design, Setting, and Participants:**

This ad hoc secondary analysis was performed between July 2023 and September 2024 and used data from a cluster-randomized, placebo-controlled trial (School Inner-City Asthma Intervention Study) of HEPA purifiers conducted from September 2015 to June 2020. A total of 200 classrooms from 39 public schools in Northeastern US were enrolled and randomized.

**Interventions:**

Classrooms were randomized 1:1 to receive either active HEPA purifiers or visually identical sham HEPA units (4 per classroom). School staff and investigators were blinded to intervention assignments.

**Main Outcomes and Measures:**

The primary outcome was high viral exposure, identified by K-means clustering of individual viral concentrations. Secondary outcomes included viral diversity (defined as number of detected virus types) and individual viral concentrations. Week-long bioaerosol samples were collected 3 times during 1 school year, and concentrations of 19 respiratory viruses were quantified by digital droplet polymerase chain reaction.

**Results:**

Of the 200 enrolled classrooms (91 in the sham purifier group and 109 in the HEPA purifier group) analyzed, the median (IQR) class size was 19 (18-20) and the median (IQR) grade was 3 (2-5). A total of 532 bioaerosol samples were collected; viruses were detected in 524 samples (98.5%), with a median (IQR) of 3 (2-5) viruses per classroom. Rhinovirus was most prevalent (476 [89.5%]), while respiratory syncytial virus A and B (66 [12.4%] and 127 [23.9%]) as well as influenza A and B (94 [17.7%] and 76 [14.3%]) were also detected. High viral exposure was present in 118 samples (22.2%). The HEPA purifier intervention was not associated with lower odds of high viral exposure (odds ratio [OR], 0.50; 95% CI, 0.08-3.25; *P* = .46) but did correspond to a modest reduction in viral diversity (β = −1.02; 95% CI, –1.68 to −0.35; *P* = .003). Elastic net regression identified relative humidity, grade, winter season, and coarse particulate matter as the environmental risk factors for viral exposure.

**Conclusions and Relevance:**

In this secondary analysis, HEPA purifiers were not associated with a reduction in high viral exposure but were associated with a modest decrease in viral diversity. Multicomponent interventions may be needed to mitigate respiratory viral exposures in schools.

## Key Points

**Question:**

Is the use of high-efficiency particulate air (HEPA) purifiers associated with reduced respiratory virus exposure in elementary school classrooms?

**Findings:**

In this secondary analysis of a randomized clinical trial of 200 classrooms, respiratory viral exposures were high; HEPA purifiers were not associated with an overall reduction in viral burden.

**Meaning:**

Mitigating respiratory viral exposures in schools may require multicomponent interventions, including addressing both air filtration and additional indoor environmental factors.

## Teaser

This secondary analysis of a randomized clinical trial examines the association of HEPA (high-efficiency particulate air) filters with respiratory viral exposure in elementary school classrooms.

## Introduction

Viral infections cause substantial morbidity in children^1,2^ and are a leading cause of school absenteeism.^3,4^ Schools, where children spend the majority of the day, are recognized as an important source of respiratory virus exposure.^5,6^ Environmental air sampling has been effective at detecting respiratory viruses, such as SARS-CoV-2 and influenza.^7,8,9^ However, comprehensive longitudinal assessments of viral exposures in schools are lacking, especially for noninfluenza respiratory viruses with pandemic potential.^10,11^ Quantifying viral exposure in bioaerosols is challenging due to low biomass^9,12^ and limitations in detecting multiple targets. Multiplexed digital droplet polymerase chain reaction (ddPCR) offers precision and sensitivity, especially in low-biomass samples.^13,14^

There is limited clinical evidence on effective measures to reduce indoor viral exposure. While high-efficiency particulate air (HEPA) filters are effective at reducing fine and coarse particulate matter (PM),^15,16,17^ their effectiveness against respiratory viruses transmitted via aerosols (<5 μm) and droplets (>5 μm) in clinical settings remains unclear due to factors such as occupancy, ventilation, indoor climate, and occupant-specific characteristics, such as age.^18,19,20^ In addition, while low-cost sensors exist for some indoor pollutants, none exists for detection of viruses. Some studies propose using carbon dioxide (CO_2_) as a surrogate marker for viral exposure^21^; however, most research on built-environment factors and viral exposures is model-based, lacking comprehensive epidemiologic evidence.

In this secondary analysis of a randomized clinical trial of HEPA purifiers in schools, we aimed to evaluate whether portable HEPA purifiers were associated with reduced respiratory virus exposures in elementary school classrooms. Secondarily, we examined the risk factors for high viral exposure.

## Methods

This is an ad hoc secondary analysis of the School Inner-City Asthma Intervention Study (SICAS-2; ClinicalTrials.gov identifier NCT02291302), a cluster-randomized, placebo-controlled trial of the effects of classroom-level HEPA purifiers and school-level integrated pest management on asthma morbidity. The study protocol and statistical analysis plan are provided in Supplement 1. During SICAS-2, elementary students aged 4 to 15 years with physician-diagnosed asthma were enrolled from public schools in Northeastern US from September 2015 to June 2020; eTable 1 in Supplement 2 provides the inclusion and exclusion criteria. This secondary analysis was conducted between July 2023 and September 2024. The Boston Children’s Hospital Institutional Review Board approved this analysis. Written parental informed consent and student assent were obtained in English or Spanish in SICAS-2. We followed the Consolidated Standards of Reporting Trials (CONSORT) reporting guideline.

SICAS-2 was powered to detect a 0.75-day difference in asthma symptoms with 90% power and α = .05, requiring 240 participants. A post hoc power analysis showed that the study had 90% power to detect the effect of the HEPA purifier intervention on high classroom viral exposure, corresponding to an odds ratio (OR) of 0.33. Per confidentiality agreements, the locations of the schools may not be disclosed. Detailed methods of the trial have been published along with its primary outcomes.^15,22^

### Participants and Randomization

Enrolled classrooms were randomized to the HEPA purifier intervention after the start of the school year. Classrooms were randomized in a 1:1 ratio to receive 4 either active or sham (placebo) portable HEPA purifiers (Model AP1013A; Coway Co Ltd) using random numbers generated with Stata, version 14 (StataCorp LLC). Students and teachers, school staff, and investigators were blinded to the intervention assignment. Randomization and follow-up were conducted during the 2015 to 2020 academic school years.

### HEPA Purifier Intervention

After baseline assessments, 4 HEPA purifiers, each with a delivery rate of 3000 L/min, were placed in each classroom.^15^ Sham purifiers were constructed by removing the filters from active purifiers and adding a sound generator to make them indistinguishable from the active purifiers. Additional intervention details are provided in the eMethods in Supplement 2.

### Baseline Classroom Assessments and Longitudinal Environmental Sampling

After enrollment, baseline classroom characteristics were assessed and week-long environmental samples, indoor air quality, and climate measures were collected in each enrolled classroom longitudinally throughout the school year (eFigure 1 in Supplement 2). Viral bioaerosol sampling was performed using open-faced samplers equipped with 5 uM polytetrafluoroethylene filters (Pall Corp) at a flow rate of 3 L/min over a 1-week period^23^; field blanks were collected as negative controls. Gravimetric measures of PM_2.5_ and coarse PM (PM_2.5-10_) with flow rate of 5 L/min, real-time CO_2_, relative humidity, and temperature (Smart Home Weather Stations; Netatmo) were collected, and air-exchange rate was calculated based on CO_2_ decay (eMethods in Supplement 2). Classroom and school characteristics were collected, including student demographics, class size (number of students per class), classroom dimension (m^3^), and type of ventilation system. Race and ethnicity were obtained from school enrollment databases. Reported racial and ethnic categories were American Indian or Alaska Native, Asian, Black or African American, Hispanic or Latino, not Hispanic or Latino, Native Hawaiian or Other Pacific Islander, White, and multiracial. Race and ethnicity data were included as part of demographic information to enable a more comprehensive and generalizable description of the study population.

### Respiratory Viral Quantification

We used ddPCR assays to perform absolute quantification of viral copies in the collected bioaerosol samples. Assays were multiplexed to simultaneously detect up to 19 different common respiratory viruses from each sample, including adenovirus; coronavirus (HKU1, NL63, and OC43); enterovirus (D68); respiratory syncytial virus (RSV) A and B; influenza A; influenza A (H1N1); influenza A (H3N2); influenza B; parainfluenza virus 1, 2, 3, and 4; human metapneumovirus; parechovirus; and rhinovirus. Viral copy number per cubic meter of air (copies/m^3^) was then calculated (eMethods in Supplement 2).

### Outcomes

The primary outcome was high viral exposure, defined using K-means clustering (eMethods in Supplement 2). In the absence of a validated threshold for airborne viral burden encompassing multiple respiratory viruses, this unsupervised approach was used to group samples based on shared patterns of viral concentration, avoiding arbitrary or subjective cutoffs. We first used within-cluster sum of squares and the silhouette method to determine that the optimal number of clusters was 2 (eFigure 2 in Supplement 2). We then compared viral concentrations between clusters. The cluster with overall higher viral concentrations was designated as the high viral exposure group. Secondary outcomes were viral diversity, defined as the total number of unique viruses detected per classroom based on the presence or absence of each virus, and individual viral concentrations.

To verify the clinical significance of high viral exposure, we looked at school absenteeism—defined as the mean number of days students were absent over 1 academic year in each school—as an exploratory outcome. Absenteeism was retrospectively collected for each academic year during which the study period occurred.

### Statistical Analysis

The eMethods in Supplement 2 provides additional details on all statistical analyses. Analyses were conducted with R, version 4.4.1 (R Project for Statistical Computing), using 2-sided tests. Statistical significance threshold was set at *P* < .05.

#### Classroom Characteristics and Indoor Air Quality

Classroom characteristics along with indoor air quality measures were summarized, stratified by HEPA purifier intervention status. Medians (IQRs) were calculated for continuous variables, and numbers (proportions) were calculated for categorical variables. Differences between groups were assessed using Wilcoxon rank-sum test for continuous variables and χ^2^ test for categorical variables.

#### Treatment Effect of HEPA Purifiers 

For our primary analysis, we conducted an intention-to-treat analysis including all classrooms with at least 1 baseline air sample collected. The main outcome of interest was the mean treatment effect of HEPA purifiers on the probability of high viral exposure, comparing preintervention and postintervention differences between treated (HEPA purifiers) and control (sham purifiers) classrooms. Secondary outcomes were compared between preintervention and postintervention periods. High viral exposure was analyzed using a generalized linear mixed-effects model with logit link. Viral diversity was analyzed using a linear mixed-effects model. Both models included fixed effects for intervention group and intervention period (before vs after), with the interaction of the 2 terms serving as the primary effect of interest. Random effects were used to account for repeated measures within each school year in classrooms and clustering of classrooms in schools.

Sensitivity analyses were performed using generalized additive mixed models, with the addition of a spline term for days since the start of school to adjust for the effect of season as a precision variable. Analyses of virus-specific changes in concentration after the HEPA purifier intervention were conducted using generalized additive mixed models. Viral concentrations were normalized using *z*-score transformation to allow for comparability. Due to multiple comparisons across individual viruses, false-discovery-rate correction was applied using the Benjamini-Hochberg method.

#### Implications of Viral Exposure for School Absenteeism

To evaluate the association between viral exposure and school absenteeism, we first calculated the proportion of classrooms with high viral exposure per year, and the mean number of viruses detected in each school. Absenteeism was similarly calculated per year in each school. We then used a linear regression model to investigate the effect of viral exposure on absenteeism.

#### Implications of Environmental Factors for Viral Outcomes

To investigate which environmental factors were associated with high viral exposure, we used a machine learning approach with elastic net regression. Missing indoor air quality measures were first imputed using K*-*nearest neighbors. Missingness primarily resulted from equipment failures and thus were assumed to be missing completely at random. Continuous variables were normalized using *z-*score transformation to ensure comparability. A random 50:50 split was performed to separate the data into training and validation datasets. Hyperparameter tuning was performed within the training dataset using 5-fold repeated cross-validation to maximize the area under the curve (AUC). Elastic net regression model performance was assessed on the validation dataset, after which the final model was refitted using the validation data to obtain coefficient estimates. Effect sizes for each factor are reported as ORs, with 95% CIs derived by bootstrap estimation. To demonstrate that variable importance was robust to machine learning approach, we also used random forest models for comparison. Additionally, complete case analysis was performed as a sensitivity analysis.

## Results

### School and Classroom Characteristics

**Figure 1.**
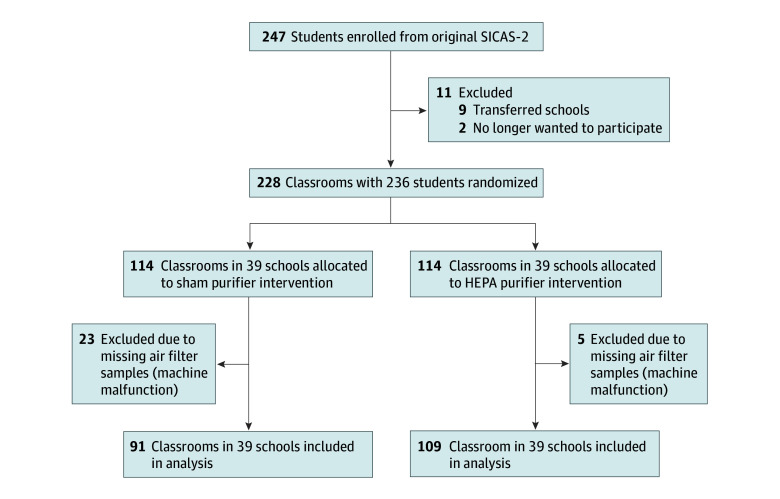
Flow Diagram of Secondary Analysis HEPA, high-efficiency particulate air; SICAS-2, School Inner-City Asthma Intervention Study.

A total of 200 elementary classrooms (91 in the sham purifier group and 109 in the HEPA purifier group) across 39 schools were included in this analysis (Figure 1). Twenty-three classrooms from the sham purifier group and 5 from the HEPA purifier group were excluded from analysis due to equipment malfunctions that prevented bioaerosol sample collection. Baseline school and classroom characteristics are presented in the Table.

**Table.** School and Classroom Characteristics and Summary of Indoor Environmental Factors

Characteristic	Total, median (IQR)	Intervention, median (IQR)		*P* value^a^
		Sham purifier	HEPA purifier	
Schools (n = 39 schools)				
Students, No.	390.5 (247.0-538.0)	NA	NA	NA
Class size, No.	18.0 (17.0-20.0)	NA	NA	NA
Low income, %^b^	59.8 (43.7-67.9)	NA	NA	NA
Sex, %				
Female	49.2 (47.6-51.5)	NA	NA	NA
Male	50.9 (49.0-53.0)	NA	NA	NA
Race and ethnicity, %^c^				
American Indian or Alaska Native	0.0 (0.0-0.4)	NA	NA	NA
Asian	2.5 (1.0-7.2)	NA	NA	NA
Black	25.0 (5.5-41.9)	NA	NA	NA
Hawaiian or Pacific Islander	0.0 (0.0-0.2)	NA	NA	NA
Hispanic	36.8 (28.2-60.0)	NA	NA	NA
White	14.5 (4.4-30.0)	NA	NA	NA
Multiracial	3.5 (2.0-4.9)	NA	NA	NA
No. of d absent per student per y	8.8 (7.5-10.3)	NA	NA	NA
Classrooms				
No. (%)	200 (100)	91 (45.5)	109 (54.5)	NA
Class size	19 (18-20)	19 (17-20)	19 (18-20)	.99
Central HVAC, No. (%)	47 (23.5)	20 (22.0)	27 (24.8)	.77
Grade	3 (2-5)	3 (2-5)	3 (2-5)	.32
Kindergarten, No. (%)	15 (7.5)	8 (8.8)	7 (6.4)	.11
Classroom dimension, m^3^	247.0 (226.0- 281.8)	244.1 (224.7-276.7)	249.5 (227.4-290.0)	.43
Preintervention indoor air quality measures				
PM_2.5_, μg/m^3^	5.4 (4.3-6.8)	5.3 (4.4-6.7)	5.6 (4.2-6.8)	.96
Coarse PM, μg/m^3^	7.4 (5.5-9.4)	7.4 (5.5-9.2)	7.5 (5.5-9.4)	.63
Relative humidity, %	50.6 (42.3-55.1)	48.3 (42.7-54.4)	51.1 (42.1-55.3)	.58
Temperature, °C	20.9 (20.0-21.7)	21.0 (20.3-21.8)	20.8 (19.7-21.5)	.11
Mean CO_2_, ppm	776.0 (675.4-901.9)	785.0 (685.8-903.0)	767.5 (674.7-898.4)	.57
Peak CO_2_, ppm	2154.5 (1668.6-2743.6)	2124.5 (1664.3-2736.7)	2206.4 (1713.9-2747.0)	.71
Air-exchange rate/h	3.3 (2.9-3.6)	3.3 (2.9-3.7)	3.2 (2.9-3.6)	.76

Abbreviations: CO_2_, carbon dioxide; HEPA, high-efficiency particulate air; HVAC, heating, ventilation, air conditioning; NA, not applicable; PM, particulate matter; PM_2.5_, fine PM; ppm, parts per million.

^a^ *P* values were derived using Wilcoxon rank-sum test for continuous variables and χ^2^ test for categorical variables.

^b^ Low income was defined as students’ participation in state-administered assistance programs.

^c^ Race and ethnicity were defined per the reporting guidelines of school districts.

Classrooms had a median (IQR) class size of 19 (18-20) students, and the median (IQR) grade level was 3 (2-5). Student demographics varied by school. The median (IQR) composition of enrolled students was 49.2% (47.6%-51.5%) females and 50.9% (49.0%-53.0%) males, of whom 25.0% (5.5%-41.9%) had Black, 36.8% (28.2%-60.0%) had Hispanic, and 14.5% (4.4%-30.0%) had White race and ethnicity. A median (IQR) of 59.8% (43.7%-67.9%) of students were from families with a low income, as defined by students’ participation in state-administered assistance programs. Median (IQR) absenteeism rate was 8.8 (7.5-10.3) days per year. Central HVAC (heating, ventilation, air conditioning) systems were present in only 47 classrooms (23.5%).

Indoor air quality measures were similar across randomized classrooms. Classroom ventilation was limited, with a low median (IQR) air-exchange rate of 3.3 (2.9-3.6) exchanges per hour and peak CO_2_ concentrations frequently exceeding recommended levels of 2000 ppm^24^ (overall median [IQR], 2154.5 [1668.6-2743.6] ppm).

### Air Sample Collection

Each classroom had a baseline sample collected before the intervention began. After intervention, 147 and 185 samples were collected for the sham purifier and HEPA purifier groups, respectively. All preintervention samples were collected from October through December, while postintervention samples were collected from January through June (eFigure 3 in Supplement 2). Due to the school shutdowns at the onset of the COVID-19 pandemic, no air samples were collected after December 2019.

### Respiratory Viral Detection in Classrooms

**Figure 2.**
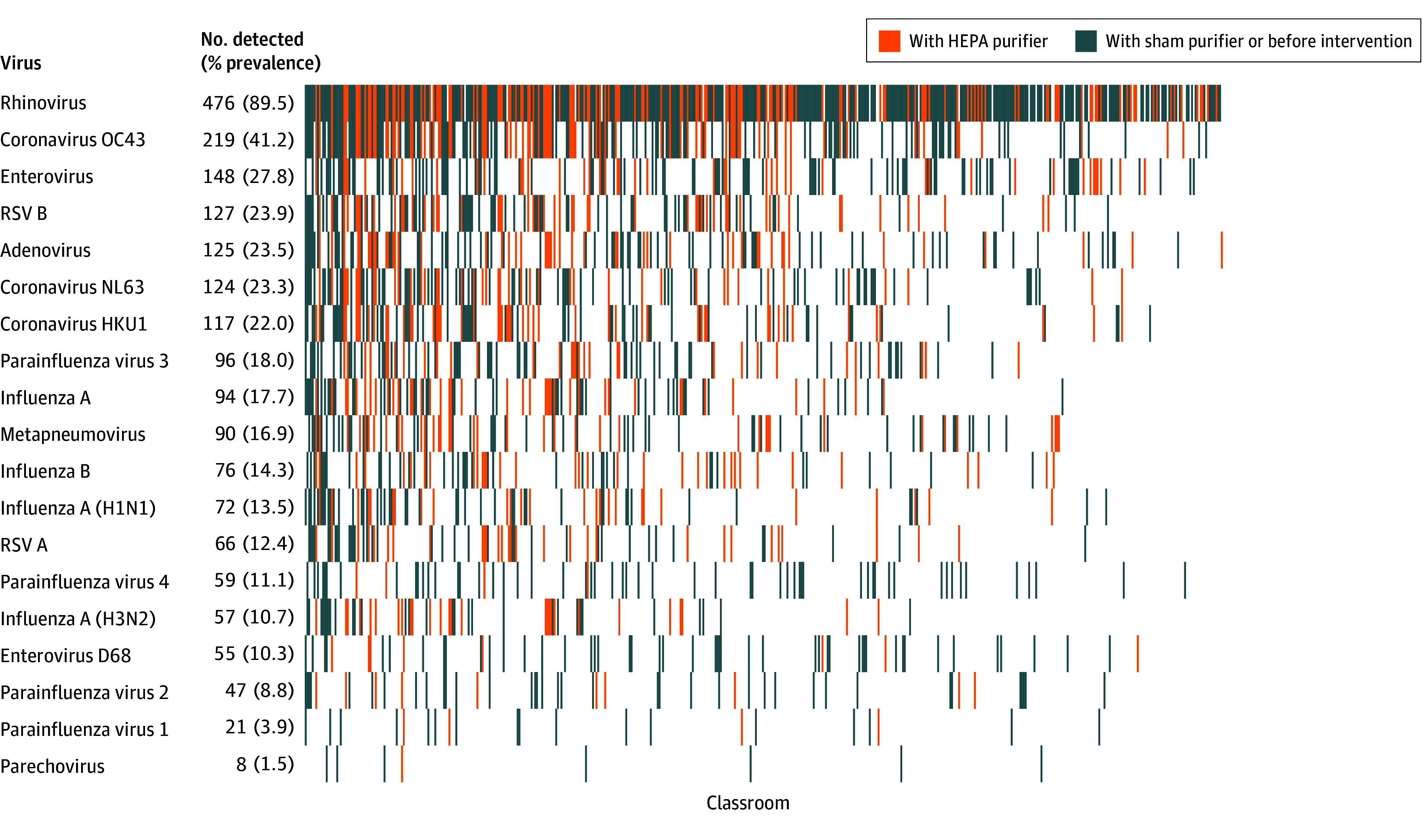
Distribution of Respiratory Viral Detection Across Classrooms Viruses are arranged from most (top) to least (bottom) prevalent, with number of detections and percent prevalence (out of 532 samples) for each virus. Individual classrooms are arranged from highest (left) to lowest (right) number of viruses detected per classroom. HEPA indicates high-efficiency particulate air; RSV, respiratory syncytial virus.

Of the 532 air samples collected, viruses were detected in 524 samples (98.5%), with a median (IQR) of 3 (2-5) and a range of 0 to 13 viruses per classroom (Figure 2). Rhinovirus was the most prevalent virus detected, present in 476 samples (89.5%). Coronavirus OC43 and enterovirus were also prevalent, detected in 219 samples (41.2%) and 148 samples (27.8%), respectively. Influenza A (pan-assay) was detected in 94 samples (17.7%) and influenza B was found in 76 samples (14.3%), while influenza A (H1N1) and influenza A (H3N2) were detected in 72 samples (13.5%) and 57 samples (10.7%), respectively. RSV A and B were detected in 66 samples (12.4%) and 127 samples (23.9%), respectively. Viral prevalence and concentrations by intervention group and period are summarized in eTable 2 in Supplement 2. Monthly patterns were observed in both viral concentration and detection rates, both of which peaked during the winter months (eFigure 4 in Supplement 2).

### Primary Outcome

Using K*-*means clustering, we identified 2 distinct groups of air samples: high viral exposure (118 [22.2%]) and low viral exposure (414 [77.8%]). The high-exposure group had higher mean (SD) concentrations of most viruses, including coronavirus OC43 (17 134 [11 607] vs 5158 [9061] copies/m^3^; *P* < .001), influenza A (8853 [11 267] vs 971 [3348] copies/m^3^; *P* < .001), and RSV A (7368 [10 930] vs 630 [3038] copies/m^3^; *P* < .001) (eFigure 5 and eTable 3 in Supplement 2). High viral exposure peaked in the winter (eFigure 6 in Supplement 2).

**Figure 3.**
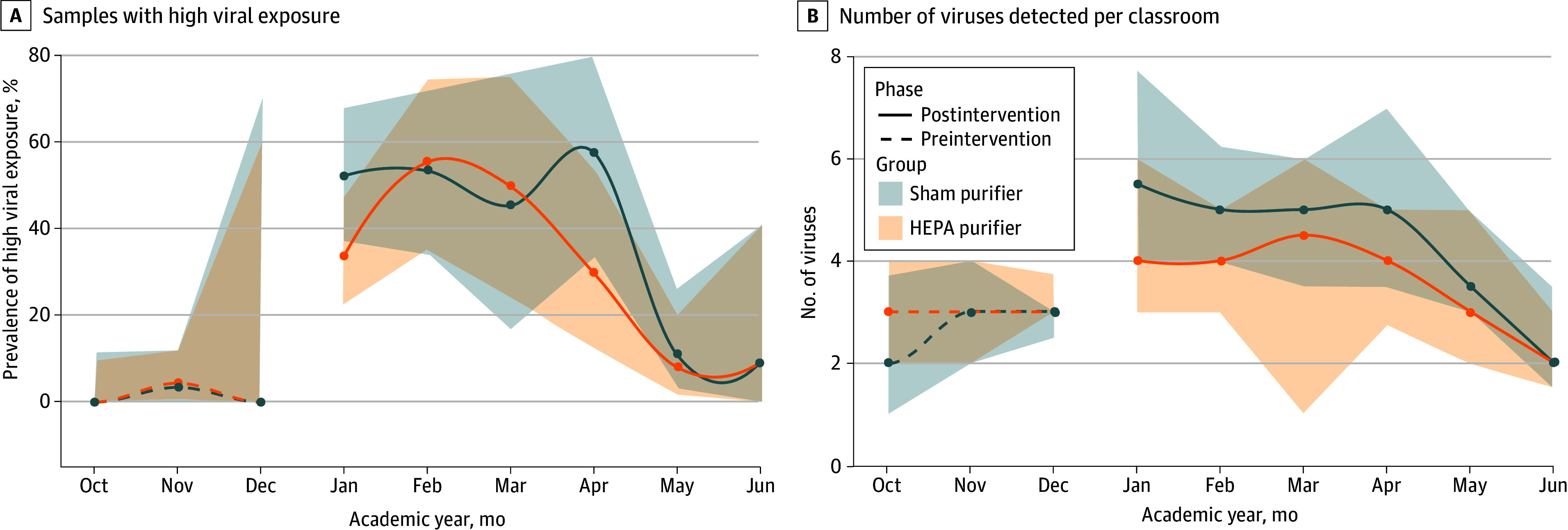
High-Efficiency Particulate Air (HEPA) vs Sham Purifier Intervention and Viral Exposure During the School Year A, Lines represent observed prevalence, and shaded areas indicate the 95% binomial CIs. B, Lines represent the medians, and shaded areas represent the IQRs.

In assessing the clinical relevance of the high viral exposure group, we found that schools with a higher proportion of classrooms with high viral exposure had almost 6 more absent days per student per year, after adjusting for class size, presence of central HVAC system, and the percentage of low-income students (β = 5.6; 95% CI, 1.6-9.7; *P* = .007). In intention-to-treat analyses, the HEPA purifier intervention was not associated with a significant reduction in the odds of high viral exposure (OR, 0.50; 95% CI, 0.08-3.25; *P* = .46) (Figure 3A), with no change after adjustment for seasonality (OR, 0.45; 95% CI, 0.07-3.07; *P* = .42).

### Secondary Outcome

The median (IQR) viral diversity was 3 (2-5), with a maximum of 13 viruses detected per classroom. Viral diversity was not associated with absenteeism (β = 0.5; 95% CI, –0.1 to 1.1; *P* = .10).

The HEPA purifier intervention was associated with lower viral diversity, with an estimated reduction of 1 virus per classroom (β = −1.02; 95% CI, –1.68 to −0.35; *P* = .003) (Figure 3B), or an overall 32.8% reduction in viral diversity. This effect was unchanged after adjustment for seasonality (β = −1.03; 95% CI, −1.65 to −0.42; *P* = .001).

Of the 19 viruses analyzed, the largest reductions in viral diversity were found for parainfluenza virus 3 (β = −0.43; 95% CI, –0.74 to −0.10; *P* = .01), coronavirus OC43 (β = −0.41; 95% CI, –0.70 to −0.12; *P* = .005), RSV B (β = −0.35; 95% CI, –0.66 to −0.03; *P* = .03), and coronavirus NL63 (β = −0.33; 95% CI, –0.64 to −0.01; *P* = .04). Here, β coefficients represent the change in viral concentration in SD units relative to the mean across all samples. However, these associations did not remain statistically significant after correction for multiple testing (eTable 4 in Supplement 2).

### Risk Factors for Classroom Viral Exposures

**Figure 4.**
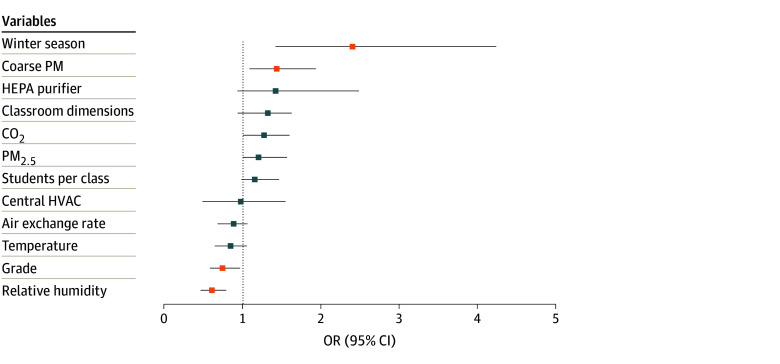
Factors Associated With High Viral Exposure Odds ratios (ORs) of indoor air quality measures and classroom characteristics were derived from elastic net regression model fitted on validation set; 95% CIs (error bars) were derived using bootstrapping with 2000 resamples. Variables are arranged from highest OR (top) to lowest OR (bottom), and variables where 95% CIs do not cross 1 are depicted in yellow. CO_2_ indicates carbon dioxide; HEPA, high-efficiency particulate air; HVAC, heating, ventilation, air conditioning; PM, particulate matter; PM_2.5_, fine PM.

After hyperparameter tuning, the optimal elastic net model for determining risk factors for high viral exposure had an AUC of 0.76. The final model, refitted on the validation dataset, achieved an AUC of 0.82, indicating good generalizability (eFigure 7 in Supplement 2). In the final model, higher relative humidity (OR, 0.60; 95% CI, 0.46-0.79) and higher grade level (OR, 0.74; 95% CI, 0.58-0.96) emerged as protective factors against high viral exposure. Conversely, the winter season (OR, 2.40; 95% CI, 1.41-4.23) and increased levels of coarse PM (OR, 1.43; 95% CI, 1.08-1.93) were identified as the most important risk factors for high viral exposure. CO_2_, temperature, PM_2.5_, and air-exchange rate were not associated with high viral exposure (Figure 4). Variable importance ranking using elastic net yielded similar results to those of random forest (eFigure 8 in Supplement 2).

In this analysis, missing data were observed for PM_2.5_ (54 [10.2%]), coarse PM (52 [9.8%]), and classroom dimensions (20 [3.8%]), along with relative humidity, CO_2_, and temperature (55 [10.3%]), Overall, 280 samples (22.4%) had at least 1 missing measure. Classrooms with missing data are summarized in eTable 5 in Supplement 2. Missing data were similarly distributed between the high viral cluster and low viral cluster groups (eTable 6 in Supplement 2). Complete case analysis showed similar findings to our analysis with imputed data (eFigure 9 in Supplement 2).

## Discussion

In this secondary analysis of SICAS-2, respiratory viruses were detected in 98.5% of air samples. Although the HEPA purifier intervention was not associated with a decrease in respiratory viral exposure overall, it was associated with a modest decrease in viral diversity. To our knowledge, SICAS-2 was not only the first large-scale, longitudinal school-based study that comprehensively quantified bioaerosol levels of 19 common respiratory viruses but was also the first randomized clinical trial that assessed the association between HEPA purifiers and viral load in the air.^20,25^ By leveraging the novel use of multiplexed ddPCR for absolute quantification of viral load in air samples,^8,26^ we overcame the limitations of low detection rates seen in prior studies using quantitative PCR.^7,9^ Furthermore, we demonstrated that high viral exposure, which peaked during winter months—the height of respiratory infections for school-aged children—was associated with increased school absenteeism.

Most studies on the effectiveness of HEPA filtration for decreasing viral load in the air are model based.^27^ The few existing studies on HEPA purifier use are limited by small sample size and do not have robust abilities to quantify viral load.^19,20,26^ While we did not find an association between HEPA purifier use and high overall viral exposure, the intervention was associated with a 32.8% reduction in viral diversity. However, the clinical significance of these changes is not clear given that we did not find an association between viral diversity and school absenteeism.

In our exploratory analysis of modifiable environmental factors associated with viral exposures, we found that higher levels of coarse PM were associated with increased viral exposure, even with HEPA purifier use, suggesting that further PM reduction may enhance the effectiveness of the intervention. Schools with limited ventilation, such as those in this study, may benefit most from using HEPA purifiers as a cost-effective solution when more comprehensive HVAC upgrades remain cost prohibitive. Additionally, low relative humidity was associated with high viral exposure, even accounting for the winter season. Model-based studies on relative humidity suggest that lower relative humidity levels below 40% could lead to longer persistence in the air of certain viruses, such as influenza and SARS-CoV-2, by promoting viral stability and suspension in aerosols.^28,29,30^ Low indoor humidity has also been shown to impair host defense against viral infection.^31^

School-aged children are particularly vulnerable to respiratory viral infections, which can result in adverse health outcomes, impaired academic performance and diminished educational attainment due to school absenteeism, and substantial economic costs related to caregiver work absenteeism.^32,33,34^ Although we did not find that the HEPA purifier intervention was associated with changes in high viral exposure, this secondary analysis was of a clinical trial powered for a different end point. Beyond air filtration or improving ventilation, complementary strategies to reduce viral exposure may include maintaining relative humidity levels between 40% and 60%, which may also enhance comfort for both students and teachers.

### Limitations

There are several limitations to our study. This secondary analysis used data from a trial that was originally powered for an asthma clinical end point; thus, the results require confirmation. Post hoc power analysis showed that we had insufficient power to detect small to modest reductions in the odds of high viral exposure. While measures were taken to prevent the HEPA cleaners from being turned off, we did not use sensors to monitor use, which may have altered the effectiveness of the intervention. We oversampled schools without central HVAC systems, which may limit generalizability, although these schools are most likely to benefit from HEPA purifier interventions due to low air-exchange rates from passive ventilation. This study was not powered to evaluate effect modification by other classroom characteristics affecting airflow. While we used a longitudinal study design, we sampled once per season rather than weekly given the enormous logistical challenges of performing large-scale school-based studies with exposure assessment. Postintervention samples were limited to winter, spring, and summer months, which limited our ability to study the full effectiveness of the intervention in the fall, when children return to school. While we assessed specific subtypes of certain viruses, such as influenza virus, due to feasibility we did not further characterize other viral clades, such as rhinovirus. Lastly, while we quantified viral load in air, we could not infer transmissibility.

## Conclusions

In this secondary analysis of SICAS-2, a randomized clinical trial of HEPA filters in elementary school classrooms, HEPA purifiers were not associated with a reduction in high viral exposure but were associated with a modest decrease in viral diversity. Future studies should further investigate multicomponent environmental interventions, such as targeting higher relative humidity, to reduce respiratory virus exposure in schools.

## Notes

Supplement 1.
Study Protocol and Statistical Analysis Plan

Supplement 2. **eTable 1.** Detailed Inclusion and Exclusion Criteria of Parent Study **eTable 2.** Prevalence and Mean Viral Concentrations by Intervention Group and Period **eTable 3.** Individual Viral Concentrations in High vs Low Viral Clusters **eTable 4.** Effect Size of HEPA Intervention on Individual Viral Concentrations **eTable 5.** Summary of Classrooms With Missing Measures **eTable 6.** Proportion of Missing Classroom Measure by Cluster **eFigure 1.** Overview of Study Design Over Each School Year **eFigure 2.** K-Means Cluster Analysis of Viral Concentrations **eFigure 3.** Overview of Sample Collection Months Based on Intervention Status and Visit Type **eFigure 4.** Monthly Trends in Respiratory Viral Exposures Across Classrooms **eFigure 5.** Comparison of Mean Concentrations of Viruses Between Clusters **eFigure 6.** Prevalence of High Viral Cluster Based on Season **eFigure 7.** Receiver Operating Characteristics (ROC) Curves for Elastic Net Regression Model on (A) Training Set and (B) Validation Set **eFigure 8.** Ranked Variable Importance of Indoor Air Quality and Classroom Characteristics in Predicting High Viral Exposure **eFigure 9.** Complete Case Analysis Using Elastic Net Model
eMethods.

eReferences.

Supplement 3.
Data Sharing Statement
