# Supplementary material for: Air Purifier Intervention for Respiratory Viral Exposure in Elementary Schools: A Secondary Analysis of a Randomized Clinical Trial
Source: JAMA Netw Open. 2025 Oct 10;8(10):e2536951. doi: 10.1001/jamanetworkopen.2025.36951 (PMC12514627; doi:10.1001/jamanetworkopen.2025.36951)
Supplement: Supplement 1. — Study Protocol and Statistical Analysis Plan [file jamanetwopen-e2536951-s001.pdf]

**Supplement 1:**

School Inner-City Asthma Intervention Study Protocol and Statistical Analysis Plan

This supplement contains the following items:

1. Full protocol of original parent study
2. Statistical analysis plan for parent study

# PROTOCOL NUMBER U01-BCH-01

## School Inner-City Asthma Intervention Study:

**VERSION 4.0 / VERSION DATE: 11/14/2015**

**IND#** *[n/a]*

**Study Sponsor(s):** The National Institute of Allergy and Infectious Diseases (NIAID)

**NIAID Funding Mechanism:** U01 AI110397-01A1

**Study Drug Manufacturer/Provider:** *[n/a]*


**PROTOCOL CHAIR- WANDA PHIPATANAKUL, MD, MS**

Professor of Pediatrics
Boston Children's Hospital
300 Longwood Avenue
Boston, MA 02115
Phone: 857-218-5336
Fax: 617-730-4709
Email:
wanda.phipatanakul@childrens.
harvard.edu

**NIAID MEDICAL OFFICER/MONITOR- PETER GERGEN, MD, MPH**
Division of Allergy, Immunology, and Transplantation – NIAID/NIH
5601 Fishers Lane
Bethesda, MD 20892-9828, USA
Phone: 240-627-3545
Fax: 301-480-4258
E-mail: pgergen@niaid.nih.gov

**BIOSTATISTICIAN- BRENT COULL, PHD**

Professor of Biostatistics
Harvard School of Public Health
655 Huntington Ave
Boston, MA 02115
Phone: 617-432-0156
Fax: 617-432-1659
E-mail: bacoull@hsph.harvard.edu

**NIAID NURSE CONSULTANT/PROJECT MANAGER-KATHERINE THOMPSON**
Division of Allergy, Immunology, and Transplantation – NIAID/NIH
PO Box 7
Lecanto, FL 34460, USA
Phone: 301-706-1177
E-mail: thompsonkath@niaid.gov

**REGULATORY OFFICER –** Ling Li Ph.D
OFFICE OF REGULATORY AFFAIRS
Division of Allergy, Immunology, and Transplantation – NIAID/NIH
5601 Fishers Lane
Bethesda, MD 20892-9828, USA
Phone: (240) 627-3765
Fax: (301) 480-1537
E-mail: lil16@niaid.nih.gov

# Protocol Synopsis

| | |
|---|---|
| **Title** | School Inner-City Asthma Intervention Study |
| **Short Title** | SICAS-2 |
| **Clinical Phase** | II |
| **Number of Sites** | 1 |
| **IND Sponsor/Number** | n/a |
| **Intervention(s)** | A. Classroom Air Filter Purifier, School Integrated Pest Management<br>B. Endpoints: Asthma Symptoms, health care utilization, rescue medication use, lung function |
| **Study Objectives** | The primary objective is to determine the efficacy of this environmental intervention to improve asthma outcomes. |
| **Study Design** | The study is a randomized, double-blind, placebo controlled, parallel Classroom Air Filter Intervention and a single-blind, controlled parallel trial of a school-wide (with targeting of participant classrooms) mouse-targeted integrated pest management (IPM) intervention. |
| **Primary Endpoint(s)** | The primary outcome measure is maximum days of symptoms/ 2 weeks. Maximum days of asthma symptoms will be assessed by questionnaire as done previously in NIAID funded in NCICAS, ICAS, and ICAC consortiums and studies[1-4]. The primary endpoint is the longitudinal vector of symptom-days at each of the five visits (baseline and follow up) for each subject. |
| **Secondary Endpoint(s)** | Secondary outcomes include: (1) school absences, (2) health care utilization, 3) Composite Asthma Severity Index adapted from the Inner-City Asthma Consortium[5], and incorporates day symptoms, short-acting beta agonist use, nights symptoms, and exacerbations (defined as systemic steroids for asthma) and lung function. Core outcomes come from the recent Asthma Outcomes Workshop[6] These data will be obtained from questionnaires, and lung function (i.e. $FEV_1$/FVC, $FEV_1$ percent predicted, percent change in FEV1 |

| | |
|---|---|
| | after short-acting beta agonist, FEF 25%-75%)with Koko spirometer<br>Degree of Exposure Reduction and Health Outcomes (Primary and Secondary) |
| **Accrual Objective** | 300 |
| **Study Duration** | 72 months total duration (accrual 60 months + follow-up period 12 months |
| **Treatment Description** | There will be four parallel groups randomized in a multi-level, factorial fashion: The student's primary CLASSROOM will be randomized to Air Filters versus Sham Control. Simultaneously, the student's SCHOOL will be randomized to receive intense IPM INTERVENTION (extermination and sealing of sealing of holes and cracks surrounding the classroom/cafeteria), education, reservoir cleaning versus control. |
| **Inclusion Criteria** | Subject and/or parent guardian must be able to understand and provide informed consent<br><br>Males and females who will be in grades K-8 (age 4-15 at randomization) during the subsequent academic school year after spring screening<br><br>Attend one of the schools that study team have permission to obtain classroom/school environmental samples for the subsequent academic school year.<br><br>Have no plans to move schools within the upcoming 12 months<br><br>Have health care insurance and asthma provider<br>Have physician-diagnosed asthma at least 1 year prior to the screening visit<br><br>Have evidence of active asthma disease as defined by at least one of the following:<br>• One asthma-related unscheduled visit to an emergency department (ED), clinic or urgent care facility in the previous 12 months<br>• One asthma-related overnight hospitalization in the previous 12 months<br>• One or more bursts of oral or injectable corticosteroids in the previous 12 months<br>• Wheezing symptoms in the past 12 months<br>• On Daily controller medications for asthma<br><br>Inclusion/exclusion criteria modeled from the NIAID funded Inner- |

| | |
|---|---|
| | City Asthma Studies)[1-4] |
| **Exclusion Criteria for Individual**<br><br><br><br><br><br><br><br><br><br><br><br><br><br><br><br><br><br><br><br><br>**Exclusion Criteria for School** | Individuals who meet any of these criteria are not eligible for enrollment as study participants:<br><br>Inability or unwillingness of a participant to give written informed consent or comply with study protocol in the judgment of the PI<br><br>Inability to perform spirometry<br><br>Lung disease, other than asthma, that requires daily medication<br><br>Cardiovascular disease that requires daily medication, excluding hypertension<br><br>Taking a beta-blocker<br><br>Currently receiving escalation (has not reached maintenance) of Immunotherapy (allergy shots)<br><br>Switching to a school where staff are not doing environmental sampling for that year.<br><br>Past or current medical problems or findings from physical examination or laboratory testing that are not listed above, which, in the opinion of the investigator, may pose additional risks from participation in the study, may interfere with the participant's ability to comply with study requirements or that may impact the quality or interpretation of the data obtained from the study.<br><br>Unable to access areas of school necessary to conduct extermination<br><br>School in extensive state of disrepair/damage as determined by Study Coordinator/PI judgment<br><br>Centralized Air Filtration or Purifying System (rare in Northeast schools) |
| **Study Stopping Rules** | The intervention will be ended if found disruptive to the classroom |

# Table of Contents


Glossary of Abbreviations ........ 10

1. Background and Rationale ........ 11

1.1. Background and Scientific Rationale ........ 11

*1.2.* Preclinical Experience: n/a This is an environmental intervention ........ 15

1.3. Clinical Studies for Intervention ........ 15

Summary of Other Relevant School-Based Intervention Studies: ........ 16

2. Study Hypotheses/Objectives ........ 17

2.1. Hypotheses ........ 17

2.2. Primary Objective(s) ........ 17

To determine whether a school/classroom-based environmental intervention (**classroom** High Efficiency Particulate Air (HEPA) filter/purifying units and **school** integrated pest management) will: ........ 17

2.3. Secondary Objective(s) ........ 17

To explore whether sensitization or other asthma phenotypes defined at baseline modify which pathways or networks of genes undergo intervention-related changes in methylation or gene expression that, in turn, are associated with improvement in asthma outcomes ........ 17

3. Study Design ........ 18

3.1. Description of Study Design ........ 18

3.2. Primary Endpoint ........ 19

3.3. Secondary Endpoint ........ 21

3.4. Exploratory Enpoint(s) ........ 22

3.5. Stratification, Randomization, and Blinding/Masking ........ 23

4. Selection of Participants and Clinical Sites/Laboratories ........ 24

4.1. Rationale for Study Population ........ 24

4.2. Inclusion Criteria ........ 24

4.3. Exclusion Criteria for Participant ........ 25

4.4. Selection of Clinical Sites/Labs ........ 25

5. Known and Potential Risks and Benefits to Participants ........ 26

5.1. Risks of Investigational Product or Intervention as cited in Investigator Brochure or Package Insert ................ 26
5.2. Risks of Investigational Product or Intervention cited in Medical Literature and/or those based on the Investigators' experience ........ 26
5.3. Risks of Other Protocol Specified Medications ........ 26
5.4. Risks of Study Procedures ........ 26
5.5. Potential Benefits ........ 28
6. Intervention ........ 28
6.1.1. Intervention #1 ........ 28
6.1.1.1. Formulation, Packaging, and Labeling ........ 28
6.1.1.2. Dosage, Preparation, and Administration ........ 29
6.1.2. Intervention #2 ........ 29
6.1.2.1. Formulation, Packaging, and Labeling ........ 29
6.2. Assessment of Participant Compliance with Investigational Agent ........ 29
6.3. Toxicity Prevention and Management ........ 29
6.4. Premature Discontinuation of Investigational Agent ........ 29
7. Other Medications ........ 29
7.1. Concomitant Medications ........ 29
7.2. Prophylactic Medications ........ 30
7.3. Prohibited Medications ........ 30
7.4. Rescue Medications ........ 30
8. Study Procedures ........ 30
8.1. Enrollment ........ 30
Screening Visit ........ 30
8.2 HEPA Air Filter Accountability ........ 35
9. Mechanistic Assays ........ 38
10. Biospecimen Storage ........ 39
11. Criteria for Participant and Study Completion and Premature Study Termination ........ 39
11.1. Participant Completion ........ 39
11.2. Participant Stopping Rules and Withdrawal Criteria ........ 40
11.3. Participant Replacement ........ 40
11.4. Follow-up after Early Study Withdrawal ........ 40

12. Overview ..... 40
12.1 Definitions ..... 41
12.1.2 Adverse Event (AE) ..... 41
N/A ..... 42
12.2.2 Unexpected Adverse Event ..... 42
12.2.3 Serious Adverse Event (SAE) ..... **Error! Bookmark not defined.**
12.2 Grading and Attribution of Adverse Events ..... **Error! Bookmark not defined.**
12.2.1 Grading Criteria ..... 42
12.2.2 Attribution Definitions ..... 43
12.4 Collection and Recording of Adverse Events ..... 43
12.4.1 Collection Period ..... 43
12.4.2 Collecting Adverse Events ..... 44
12.4.3 Recording Adverse Events ..... 44
12.5 Reporting of Serious Adverse Events and Adverse Events ..... 44
12.5.1 Reporting of Serious Adverse Events to Sponsor (DAIT/NIAID) ..... 44
12.5.2 Reporting to Health Authority ..... 45
Not Applicable ..... 45
12.5.3 Reporting of Adverse Events to IRBs ..... 45
12.7 Reporting of Unanticipated Safety Information ..... 45
12.8 Review of Safety Information ..... 45
12.8.1 Medical Monitor Review ..... 45
12.8.2 Data and Safety Monitoring Board(DDSMB) Review ..... 45
13. Statistical Considerations and Analytical Plan ..... 46
13.1 Overview ..... 46
13.2 Outcomes (Described in 1.1-1.3) ..... 46
Primary Outcome(s) ..... 46
Maintenance of Trial Treatment Randomization Codes ..... 46
13.4 Analysis Plan ..... 47
13.4.1 Analysis Populations. ..... 47
13.4.2 Primary Analysis of Primary Endpoint(s) ..... 47

13.4.3 Supportive Analyses of the Primary Outcome ..... 49
13.4.4 Analyses of Secondary and Other Endpoint(s)/Outcome(s) ..... 49
13.4.5 Analyses of Exploratory Endpoint(s) ..... 52
13.4.6 Descriptive Analyses ..... 52
Descriptive Analyses ..... 52
13.5 Interim Analyses (Not Applicable: Environmental Intervention- NOT a medication, high risk intervention) ..... 53
13.6 Statistical Hypotheses ..... 53
**Aim 1:** To determine the effectiveness of a school/classroom based environmental intervention [**Classroom** particle air filter/purifying units and school-wide integrated pest management (IPM)] to: ..... 53
13.7 Sample Size Considerations Sample Size Estimates: ..... 53
14. Identification and Access to Source Data ..... 57
14.1. Source Data ..... 57
14.2. Access to Source Data ..... 58
15. Protocol Deviations ..... 58
15.1. Protocol Deviation Definitions ..... 58
15.2. Reporting and Managing Protocol Deviations ..... 59
16. Ethical Considerations and Compliance with Good Clinical Practice ..... 59
16.1. Statement of Compliance ..... 59
16.2. Informed Consent Process ..... 59
16.3. Privacy and Confidentiality ..... 60
17. Quality Control and Quality Assurance ..... 60
18. Publication Policy ..... 61
19. References ..... 61

## Glossary of Abbreviations

| | |
|---|---|
| ACT | Asthma Control Test |
| CASI | Composite Asthma Severity Index |
| CFR | Code of Federal Regulations |
| CRF | Case Report Form |
| CTCAE | Common Terminology Criteria for Adverse Events |
| DAIT | Division of Allergy, Immunology, and Transplantation |
| DBPC | Double-Blind, Placebo Controlled |
| DCC | Data Coordinating Center |
| DSMB | Data Safety Monitoring Board |
| FDA | Food and Drug Administration |
| FeNO | Fractional Exhaled Nitric Oxide |
| FEF 25-75% | Forced expiratory flow between 25-75% of vital capacity |
| $FEV_1$ | Forced expiratory volume in 1 second |
| FVC | Forced Vital Capacity |
| GCP | Good Clinical Practice |
| HEPA | High Efficiency Particulate Air |
| ICAC | Inner-City Asthma Consortium |
| ICAS | Inner-City Asthma Study |
| ICH | International Conference on Harmonization |
| IEC | Institutional Ethics Committee |
| IMM | Independent Medical Monitor |
| IND | Investigational New Drug |
| IPM | Integrated Pest Management |
| IRB | Institutional Review Board |
| ISM | Independent Safety Monitor |
| MAAIT | Mouse Allergen Asthma Intervention Trial |
| MOP | Manual of Procedures |
| n/a | Not Applicable |
| NHLBI | National Heart Lung Blood Institute |
| NIH | National Institutes of Health |
| NIAID | National Institute of Allergy and Infectious Diseases |
| $NO_2$ | Nitrogen Dioxide |
| PC | Protocol Chair |
| PEF | Peak Expiratory Flow |
| PFT | Pulmonary Function Test |
| PI | [Site] Principal Investigator |
| PM | Particulate Matter |
| PM | Project Manager |
| SACCC | Statistical and Clinical Coordinating Center |
| SAE | Serious Adverse Event |
| SAP | Statistical Analysis Plan |
| SAR | Suspected Adverse Reaction |
| SICAS | School Inner-City Asthma Study- observational study |

| | |
|---|---|
| SICAS-1 | School Inner-City Asthma Study-observational study |
| SICAS-2 | School Inner-City Asthma Intervention Study – proposed clinical trial |
| SOP | Standard Operating Procedure |

# 1. Background and Rationale

## 1.1. Background and Scientific Rationale

**A) Impact and Significance** Asthma affects 12-15% of children in urban United States, accounts for over 14 million missed school days per year[7], and costs billions of dollars in health care utilization despite aggressive measures to identify remediable causes[8].

**B) Scientific Background and Rationale**
Elementary school children spend 7 to 12 hours a day in school, (primarily in one classroom), and school is nearly every child's "required occupation" and source of multiple environmental exposures. The **S**chool **I**nner-**C**ity **A**sthma **S**tudy-**1** (SICAS-1) (RO1 AI 073964, Phipatanakul) is the first obsetrvational American study to comprehensively evaluate the role of urban exposures in school, classroom and home environments and asthma morbidity[9,10]. SICAS-1 showed that classroom-specific mouse allergen, mold, and particulate pollutant exposures are independently associated with symptom worsening[11-13]. Until SICAS-1, most studies have focused on home exposures to allergenic and pollutant exposures and their associations with asthma morbidity[14]. A number of home-based trials have demonstrated that targeted interventions (including air filter units) are effective in decreasing asthma morbidity[1,15-17]. Using established integrated pest management measures, we are currently conducting an NIAID-funded home intervention clinical trial (AI 083238) to evaluate mouse-targeted IPM effects on asthma morbidity[18]. SICAS 1 provided the rationale for SICAS 2, which is a school based environmental intervention clinical trial targeted towards school-specific exposures identified in SICAS 1 as important.

In schools, we have demonstrated that we can effectively decrease classroom-specific toxic exposures during the academic school year by 75% to 90% utilizing *cleverly engineered, effective, and quiet* classroom-suitable **H**igh **E**fficiency **P**articulate **A**ir (HEPA) air filters.

**C) Innovation and Advancement of the Field:** Our next step is to apply successful school/community-based strategies to determine whether a school/classroom intervention will efficiently and effectively improve asthma morbidity by reducing harmful exposures. **This is an unprecedented, high impact opportunity to test whether we can efficiently benefit a community of children in the school environment as opposed to individuals in single homes.** Our goal is to determine the efficacy of a two-pronged intervention using **classroom** particle air filter units and **school-wide** targeted IPM/cleaning in reducing asthma morbidity in urban school children. *Furthermore, we aim to link molecular mechanisms induced by our clinical intervention and ascertain whether environmental control can induce disease-modifying effects on the epigenome/transcriptome.*

**D) Knowledge and Unmet Need Fulfilled from this Study to Reduce the Impact of this Public Health Concern and Sustainability**

Our study is the first ever comprehensive school-based environment intervention trial of HEPA filtration and IPM to improve asthma. Our engineers have unique skills and experience which they applied to development, tailoring and successful piloting of unique quiet high efficiency air cleaner technology (and matched sham filter controls) which we have demonstrated to be effective in reducing exposures and acceptable in the classrooms setting. Our established,

successful community relationships over the past decade make us uniquely positioned to give back to the community who, after participation in SICAS-1, [4] want this trial. Our study has potential for huge impact. Rather than just intervening for one asthmatic child in one home at a time, we aim to effectively and efficiently improve the school environment, and by so doing, improve control of asthma, one of the most prevalent and costly chronic diseases of childhood, in a large community of vulnerable symptomatic urban children.

Our school-based IPM and air cleaner trial will have particular relevance to long-term public policy and planning for disadvantaged urban U.S. schools with similar indoor environments, where, as we found in Northeast, 17% of elementary school children wake up at night because of asthma and 15% have missed school or medical care for asthma in the past year. Our proposed interventions will likely result in net savings, where implementation costs are offset by fewer symptom-days and improved quality of life for children, less health care utilization and less loss of work-days (greater economic productivity) for caregivers. Previous indoor environment intervention trials focused on individuals in single homes. If we demonstrate that reduction of classroom-specific exposures leads to improved asthma outcomes, then our findings can be translated into efficient and cost-effective strategies to benefit communities of children through improving the school environment, where children in America spend the majority of their day.

**Rationale for Selection of Intervention**

A) **Nature of Intervention to be Tested:**

**Classroom Environmental Intervention:** After randomization, active or sham (placebo) HEPA air filters will be placed in the primary home classroom, where elementary students spend the majority of their day. The students, staff, and investigators will be blinded to active versus sham. The Air Filters: AP103A model(Woongjin Coway Co., Ltd; Oreck Air Instinct™ 200, Oreck Direct, LLC) captures particles down to 0.1 micron in size and has a maximum clean air delivery rate (CADR) of 160 $ft^3$/min (CFM) for dust. The filter is designed for rooms up to 400 sq. ft., 4 filters worked well in our Pilot and well-received in classroom/school setting. **School Intervention:** The Integrated Pest Management (IPM) School will receive an IPM strategy (extermination with rodenticide, traps, and sealing of holes and cracks), air filters, cleaning reservoirs, and education regarding pest control measures. The intervention procedures will be those that were used in the pilot intervention study,[18] and the ICAS Pongracic Study[15]**,** and the current NIAID funded MAAIT Study (AI 083238). The school-specific IPM will focus on surrounding areas that feed into the classroom and harbor infestation by food and water sources (i.e. cafeteria). This is modeled after the home interventions that work by focusing on the child's bedroom, surrounding areas, and the kitchen. The home-based strategies have been proven as effective and cost-effective strategies for reducing pertinent allergen exposure and improving health outcomes in homes[1]. Our school-based strategies will focus on the child's primary classroom, surrounding areas, and the cafeteria. Unlike home intervention strategies, where it is impossible to blind, the School IPM strategies may also be single blinded, because the students attend school during the day, and the IPM will be conducted after hours when the students are home. Therefore, staff will know which school is randomized to IPM but the students will be blinded.

**B) Stage of Intervention** Phase III Environmental Intervention with Extensive Home-based Clinical experience that will be described in section 1.4

**C) Mechanism of action of the Intervention** Reducing classroom/school environmental exposure to particulate matter and its associated allergen and mold particles. Targeted integrated pest management to further reduce mouse allergen.

## D) Rationale for the Intervention Targets to Address the Overall Study Objective (Described in Section 2)

**RATIONALE FOR CLINICAL TRIAL INTERVENTION TARGETS: Mouse, Mold and Particle Pollution**

**1.1. Supporting Data (Clinical Aim 1):Overview of SICAS-1:** SICAS-1, our school-based inner-city observational study, was designed to assess the relation of classroom/school environment allergen and mold exposure to asthma morbidity, adjusting for home exposure. It is the most comprehensive study of its kind ever performed in the United States[4,9,19-24]. Every spring, **screening surveys** in English or Spanish were distributed to all students at 8-10 elementary schools. *Of 7032 school children screened, 22% had a history of asthma, which for most children was active and involved health care utilization: 15% of the 7032 had asthma-related missed school days or medical care in the past 12 months, and 17% had night time asthma symptoms.*

From the 700-1000 surveys collected each spring, children with asthma were recruited for extensive summer baseline phenotypic assessments, including allergy and lung function testing. Every year approximately 75-100 students were enrolled from 8-10 unique schools. School/classroom visits were conducted twice during the academic school year, approximately 6 months apart, to perform week-long school floor dust, air, airborne mold sampling, and inspections using standardized methods[4,25]. Home dust samples and inspection were also obtained. Standard measures[1] (**Table 1**) of maximum number of symptoms per 2 week period, school absences for asthma, caregiver and health care utilization data were collected 3, 6, 9, and 12 months after summer baseline assessments. During the academic school year we also measured follow up lung function and fractional exhaled NO (FeNO). We assessed associations of school, classroom and home specific exposure data to child health outcomes obtained during the academic school year. *Seventy-percent of enrolled children had at least one positive skin test. While specific sensitization to mouse was associated with slightly higher symptom counts (**Fig. 2**), the association of mouse allergen level with maximum symptom counts /2 weeks did not differ for those with any sensitization vs no sensitization. Our findings reported below (Sections **1.1.a, b, and c**) support our hypothesis that our interventions will benefit all the asthmatic children.*

**1.1.a. SICAS-1 Mouse Allergen (Figure 1), Mold Allergen and Particle Pollution Classroom/School Exposures:** *We measured exposures in all 36 schools and 241 classrooms of participating students (N=351). All schools and 99% of classrooms had detectable mouse allergen. Compared to participants' home levels[9,10], their classroom levels of mouse allergen were consistently higher[20].* Levels were markedly above levels shown to increase asthma morbidity in other studies[14] and comparable to those seen in occupational research facilities that house/breed mice[26]. School cat and dog allergen levels were variable[9] and not at levels previously shown to worsen symptoms[27]. While there was very little dust mite or cockroach allergen, we measured significant dust and airborne mold levels in the schools[21]. As well as reducing mouse exposures, our SICAS-2 intervention (IPM/cleaning and HEPA air filters) will decrease molds and other allergen or irritant components of particles (see **Sections 1.2 and 1.3** for preliminary data demonstrating intervention efficacy).

**1.1.b. Classroom MOUSE Exposure and Asthma Symptoms (Figure 2):** SICAS-1 enabled us to identify the *independent* role of classroom mouse allergen exposure in exacerbation of asthma symptoms, adjusting for potential confounders: home environmental tobacco smoke, *endotoxin,* household income, gender, age, race, body mass index[28], perceived stress[29] cockroach and home mouse exposure. Upon *updating analyses since the last submission to include our full asthma cohort, we observe that higher mouse allergen levels in classrooms were associated with higher asthma symptoms in the school year for all study participants, and these associations were not modified by specific or any sensitization. Overall a 10-fold increase in mouse allergen levels was associated with 45% more symptom days (95% CI: 9% to 91%, p=0.01) per 2 week period. Given that classroom mouse allergen levels were as high as in animal labs[30], we are not surprised to find associations of mouse allergen with increased asthma symptoms in non-sensitized as well as sensitized children. In occupational lab animal settings, twice as many lab techs have symptoms than are specifically sensitized, suggesting non-allergic effects of mouse allergen or mouse-associated particle exposures[31].*

**<u>Classroom MOLD Exposure and Multiple Asthma Outcomes:</u>** For clinically relevant taxa (e.g., Penicillium/ Aspergillus, Alternaria)[32], our classroom mold levels were also higher than those identified in home-based inner-city studies linking mold to increased asthma symptoms in children[33]. *Previously we reported associations of mold with symptoms only for mold-sensitized students.[12] Subsequent preliminary analyses for the full SICAS-1 cohort show that regardless of sensitization status, higher classroom air Alternaria was linked to more missed sleep due to asthma, changed caregiver plans [OR=1.96(1.27, 3.02); p=0.002 for a SD increase in Alternaria level], and a higher composite outcome score (more dyspnea, nocturnal wheeze, limited activity, rescue medication) [OR=1.73(1.19, 2.52)]. There is ample evidence that molds in home/school environments can worsen symptoms via not only allergic, but also non-allergic, irritant mechanisms.[33-35]*

**<u>Classroom Pollution Exposure and Asthma Symptoms (Figure 3):</u>** Pollution has been shown to worsen asthma in urban children[36]. SICAS-1 was not funded for air pollution assessment but outside funds enabled us to measure classroom-specific air pollutants over 1 week periods [integrated $NO_2$, black carbon (BC), $PM_{2.5}$; continuous $PM_{2.5}$] in a subset of classrooms. In SICAS-1 students, we demonstrated a exposure-response relationship between increasing concentrations of indoor $NO_2$ (ppb) and more asthma symptom-days, (adjusted relative rate (RRadj)=1.05 per unit increase in $NO_2$ levels (95% CI= 1.02, 1.08), p=0.002)[13]. $NO_2$ is likely a marker for local traffic and regional pollution infiltrating indoors,[37] given the absence of indoor school sources for this pollutant. We found similar exposure-response relationships of BC and $PM_{2.5}$ with increased symptoms;power was limited by the fact that we had fewer indoor measurements for these two pollutants.

**<u>1.2. Pilot HEPA Filter Intervention:</u>** We acknowledge that there is a paucity of data relating school-specific interventions and asthma/allergic symptoms. We conducted a pilot evaluation of HEPA filters (AP1008BH and AP1008CH model, Woongjin Coway Co., Ltd; Oreck Air Instinct™ 200, Oreck Direct, LLC) in two neighboring classrooms of approximately 1000 $ft^2$ in the normal classroom setting (control and intervention). Environmental sampling was conducted at baseline, during a two-week intervention and immediately after the intervention to compare exposure levels between the control and intervention classrooms at baseline, during, and post intervention. **Particle Reduction:** Our pilot study demonstrated a **75% reduction in $PM_{2.5}$** from the HEPA particle filter/purifiers after adjusting for natural ambient particles in control classrooms. Using the same study design, in another busy building, when windows were closed or minimally opened we demonstrated an **87-97% reduction in particles** and a 74% reduction when windows were opened 2.2 cm.

**<u>Allergen/Mold Reduction:</u>** Classroom HEPA air filters alone **reduced airborne mouse allergen by 75%** compared to control classrooms but did not reduce settled dust levels (which we plan to reduce with IPM). We found similar effects of HEPA filters in reducing classroom cat and dog allergen levels, which were, as previously documented, low to begin with, and therefore, likely of limited clinical significance[38]. We also demonstrated that classroom HEPA filters reduced mold levels (measured by Burkhart Mold Samplers as in SICAS-1) by **79%** in one day. Mold levels rose back up to baseline when the air filter/purifiers were turned off.

**<u>1.3. Pilot IPM Intervention:</u>** With donated resources, we conducted Integrated Pest Management (IPM) targeting mouse allergen in the school, utilizing similar methods conducted in homes[18] and the NIAID home IPM study (AI 083238). IPM included inspection, trapping with glue and snap traps, and exclusion (door sweeps and filled heating pipe holes with school maintenance). Within a couple months of **school-wide** IPM (focused on cafeteria/classroom, targeting surrounding reservoirs), we were able to effectively r**educe classroom mouse allergen levels in settled dust by 75%.**

***Summary*. Our observational SICAS-1 study provides the rationale for our Clinical Trial Intervention Targets in SICAS-2.** *We have demonstrated that **classroom** mouse allergen, mold, and air pollution exposures are highly prevalent and are important influences on childhood asthma morbidity. Moreover we have shown that we can reduce these exposures in our real world urban school settings. Addressing reviewer concerns that only children with specific sensitization to mouse or mold would benefit from SICAS-2 interventions --reduction of classroom airborne particles (including particle-associated mouse and mold allergens as well as pollution) and pest management of mouse and other associated exposures-- our data suggest that all symptomatic asthmatic school children will benefit.*

**E) Advantages of Intervention to be Tested:** SICAS-2 will be a factorial design with the classroom randomized double-blind, placebo controlled to Air Filter/purifier and school being randomized in a parallel fashion to an intervention of IPM/Education/Cleaning versus Control School. This allows the advantage of understanding the classroom specific benefits of the air filter/purifier and school wide (focused on classroom/cafeteria, and surrounding supporting areas) effects of the IPM intervention. Factorial designs have been validated and established[39,40] as an efficient use of resources by answering the effects of two interventions. The classroom intervention with the parallel school wide intervention allows us to maximize impact and efficiency of answering two questions in one trial.

**Advantages and Impact:** *Schools are equivalent to occupational settings: we have shown that exposures (specially to mouse) can be at occupational levels. Approaches to reduce exposure in the work place are relevant for improvement of asthma in large populations of school children. Our study is the first ever comprehensive school-based environment intervention trial of HEPA filtration and IPM to improve asthma. Our engineers have unique skills and experience which they applied to development, tailoring and successful piloting of unique quiet high efficiency air cleaner technology (and matched sham filter controls) which we have demonstrated to be effective in reducing exposures and acceptable in the classrooms setting. Our established, successful community relationships over the past decade make us uniquely positioned to give back to the community who, after participation in SICAS-1,* [4] *want this trial. Our study has potential for huge impact. Rather than just intervening for one asthmatic child in one home at a time, we aim to effectively and efficiently improve the school environment, and by so doing, improve control of asthma, one of the most prevalent and costly chronic diseases of childhood, in a large community of vulnerable symptomatic urban children.*

***1.2.*** **Preclinical Experience:** n/a This is an environmental intervention

### 1.3. Clinical Studies for Intervention

**Home Allergen Exposure, Sensitization, and Asthma Morbidity:** Allergen exposure in home environments is an important trigger in 80% to 90% of children with asthma[41,42]. It is well established from multiple cohorts, particularly NIAID funded National Cooperative Inner- City Asthma Study (NCICAS) and Inner-City Asthma Study/Consortium (ICAS/ICAC) that certain unique inner-city allergenic exposures in home environments such as cockroach[43-45] and rodent allergen[18,46-53] [14] are highly prevalent. These and other previous studies have shown that children who are sensitized to cockroach, mouse, and rat allergen have increased asthma symptoms and morbidity when exposed to that specific allergen in the home. Mold, such as Alternaria, Aspergillus, Cladosporium, and Penicillium, may be an important respiratory irritant or allergen in home environments and increase asthma morbidity even in asthmatic children not sensitized to mold[33,54-56] . The Inner-City Asthma Study, showed that particulate matter from outdoor pollution was

highly prevalent in homes[57], and that higher outdoor concentrations of $NO_2$ (from auto emissions) and indoor particulate pollutants were associated with asthma morbidity in urban children with asthma[36]. High indoor particulate-matter was associated with decreased lung function, greater respiratory symptoms, more frequent use of rescue medications [58,59], and evidence of inflammation as measured by elevated exhaled nitric oxide levels in asthmatic children[59].

**Home Based Allergen/Mold Abatement Intervention Studies:** There has been significant attention to the potential role of intervention measures in inner-city homes to reduce allergen exposure (particularly mouse, dust mite, and cockroach) and asthma morbidity in sensitized individuals[60-64]. Review of these data concluded that interventions to reduce these allergen levels should be routine in the management of allergic asthma and have shown benefit in reducing asthma symptoms and morbidity, particularly in those sensitized to the specific allergen[60,65-67,68-71]. In the Inner-City Asthma Study (ICAS), a recent comprehensive home intervention targeted at allergens to which the child was sensitized was found to be cost-effective[72] and helpful in decreasing asthma symptoms[1]. ICAS also demonstrated children sensitized to mouse allergen who had targeted mouse allergen intervention resulting in 50% reduction in home exposure demonstrated reduced asthma morbidity[15].

In the ICAS, a sub-study in 40 children that decreased mouse allergen by 50% regardless of baseline levels, were associated with decreased morbidity[15]. In only 18 Boston homes (12 intervention 6 controls), decreasing mouse allergen by 75% reduced symptom days by 1/week, but was just not powered for significant effects[18].

Recent publications from this work did determine that mold exposure was associated with symptoms and that simple home-based interventions that have worked in NCICAS and ICAS were beneficial for these children. In a Hurricane Katrina study, simple intervention of education and providing caretakers with HEPA filters showed reductions of mold counts by 50% and reduction of symptoms[73].

We are currently conducting an NIAID funded intervention trial in Boston and Baltimore (AI 083238) using an established targeted method to decrease mouse allergen in homes of children with asthma[18]. While not a randomized controlled trial, we have evidence from our partners in the Children's Hospital Boston Community Asthma Initiative that home based education and Integrated Pest Management (IPM) referrals improved health outcomes and was highly cost-effective in reducing hospital and health care utilization costs[74], suggesting interventions to reduce exposures are important in reducing asthma morbidity.

**Air Pollution Intervention in Homes**: Regardless of baseline levels, a New Zealand home intervention by using gas flues to reduce $NO_2$ from gas stoves (geometric mean 7.3 mcg/m3 versus 10.9 mcg/m3 in controls, $P<0.01$), resulted in significant reduction in asthma symptoms[75].

**Summary of Other Relevant School-Based Intervention Studies:**
There is a paucity of comprehensive data on school/classroom based environmental intervention studies and its effects on asthma morbidity. One Swedish study showed that levels of airborne cat allergen can be reduced by allergen avoidance measures in 2 classrooms, using special school clothing or pet ownership ban[76], while another Swedish study found that a term of curtain, upholstery, bookshelves and cupboard replacement along with increased regular cleaning did not alter cat allergen levels[77]. The same group found that increased cleaning measures to decrease allergen levels may have decreased self-reported asthma symptoms, but these studies did not influence actual cat allergen levels [78].

**Mold Intervention in Schools**: Pilot health benefits were demonstrated in a mold intervention in only two (one intervention one control) Finnish schools[34] with 50% reduction in mold levels. In a comparison of only two schools (one with intervention and one without), there were significant differences in symptoms by reducing mold spore levels 50%[34].

**$NO_2$ air pollution Intervention in Schools:** In one Australian school study an intervention replacing unflued gas heaters with electric heaters among 199 students (45 intervention and 73 controls) attending 18 schools effectively reduced $NO_2$ (Intervention mean (standard deviation) 15 ppb (6.6) and Control 47 ppb (26.8) and improved asthma symptoms as measured by difficulty breathing during the day (Relative Risk [RR] = 0.41; 95% CI: 0.07, 0.98) and night (RR = 0.32; 95% CI: 0.14, 0.69), chest tightness during the day (RR = 0.45; 95% CI: 0.25, 0.81), and daytime asthma attacks (RR = 0.39; 95% CI: 0.17, 0.93)[79]. Most schools in the United States no longer have active kitchens with stoves so the indoor source of $NO_2$ as seen in the Australian study is not applicable in America.

## 2. Study Hypotheses/Objectives

### 2.1. Hypotheses

Our central hypothesis is that reducing classroom/school exposure to particles and their associated allergens (particularly mouse/mold) will decrease asthma morbidity in students with asthma. In **S**chool **I**nner-**C**ity **A**sthma Intervention **S**tudy-**2** (**SICAS-2**) we plan to test our central hypothesis in an intervention study of 300 elementary school-aged children with asthma from multiple classrooms in 40 northeast inner-city elementary schools.

### 2.2. Primary Objective(s)

To determine whether a school/classroom-based environmental intervention (**classroom** High Efficiency Particulate Air (HEPA) filter/purifying units and **school** integrated pest management) will:
(a) Reduce asthma symptoms (primary outcome as used in other NIAID inner-city asthma studies[1,3]); and
(b) Reduce medication use, health care utilization, and improve lung function (secondary outcomes).

### 2.3. Secondary Objective(s)

To explore whether sensitization or other asthma phenotypes defined at baseline modify which pathways or networks of genes undergo intervention-related changes in methylation or gene expression that, in turn, are associated with improvement in asthma outcomes.

**Mechanistic Objective:**

**Hypothesis -** Growing evidence suggests that asthma phenotypes are sustained by gene expression alterations programmed through modifiable epigenetic mechanisms[80] .

To test the hypothesis that effects of school/classroom-based environmental interventions on symptoms/other measures of asthma control occur through changes in gene methylation or expression in pathways (and secondarily, in genes) relevant to airway function and asthma. Within pathways or networks of genes (pre-specified or discovered in

unsupervised analyses) we will (a) determine how our interventions influence changes in airway cell methylation or gene expression and (b) evaluate how our intervention-associated changes in methylation and gene expression influence asthma outcomes. Finally, using mediation analysis, we will (c) estimate how much of the intervention effects on asthma symptoms occur through methylation/expression changes[81,82].

# 3. Study Design

## 3.1. Description of Study Design

SICAS-2 is a factorial, randomized, placebo controlled, parallel group phase II clinical trial designed to assess the efficacy of a classroom (Air Filter) and, randomized, controlled, parallel group school (IPM) environmental intervention in improving asthma control in children with asthma. SICAS-2 is a single-center environmental intervention study. Three hundred children attending one of the Schools in the northeast metropolitan are (total of 40 schools) from September thru June in grades K to 8th (generally ages 4-15) will be randomized to one of the four groups :

(1) IPM School/Classroom Air Filter

(2) IPM School/Classroom Sham (Placebo) Air Filter

(3) Control School/ Classroom Air Filter and

(4) Control School/Classroom Sham (Placebo) Air Filter.

Each randomized participant will be followed for a total of about 12 months, approximately 6-12 weeks prior to randomization and 10 months following randomization.

**Figure 1 SICAS-2 Annual Schema**

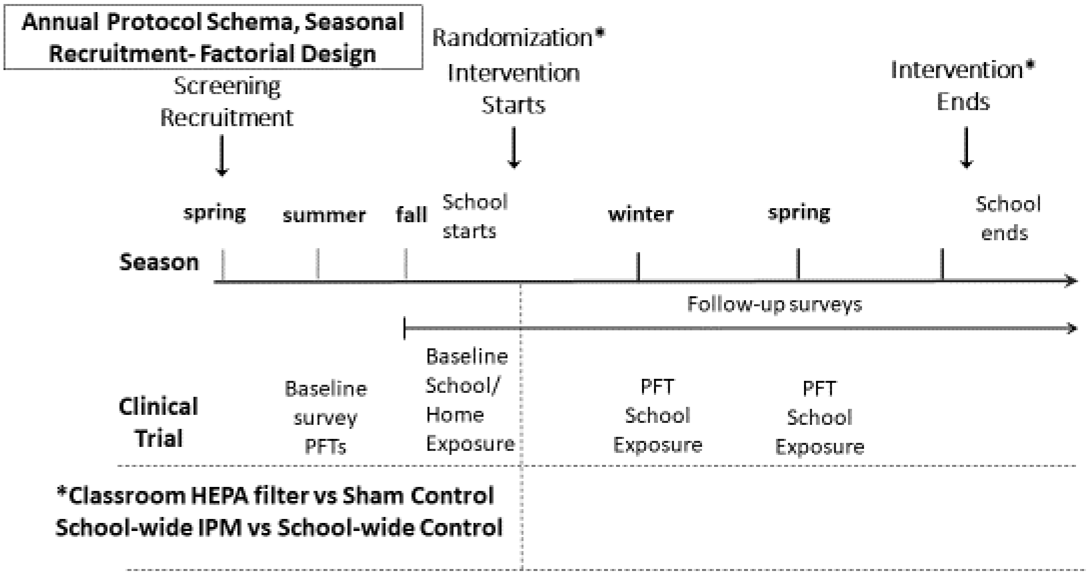


*Arms of SICAS-2 Study

| | |
|---|---|
| Cohort A - ARM 1 | SCHOOL: IPM INTERVENTION<br>CLASSROOM: AIR FILTER |
| Cohort B - ARM 2 | SCHOOL: IPM INTERVENTION<br>CLASSROOM: SHAM (PLACEBO) AIR FILTER |
| Cohort C - ARM 3 | SCHOOL: CONTROL<br>CLASSROOM: AIR FILTER |
| Cohort D - ARM 4 | SCHOOL: CONTROL<br>CLASSROOM: SHAM (PLACEBO) AIR FILTER |

### 3.2. Primary Endpoint

**See Table 1**. The primary endpoint is the rate of maximal number of days with asthma symptoms in the previous two weeks before the clinical outcome interview, defined as the largest value among the following three variables: number of days with wheezing, tightness in the chest, or cough; number of nights with disturbed sleep as a result of asthma; and number of days on which the child had to slow down or discontinue play activities because of asthma. The primary endpoint is the longitudinal vector of symptom-days at each of the visits for each subject ascertained at baseline and every two months follow up during the school year after randomization. We have proposed to include visit number, which will does not serve as a confounder but will remove any seasonal variation in symptoms exhibited independent of intervention groups. This is the main advantage of using the vector of multiple visit-specific symptom outcomes as the outcome, rather than the average of the values over the post-randomization period and allows us to control for

covariates. Maximum asthma symptom days over two weeks will be compared between the 4 groups: (1) IPM School/Classroom Air Filter (2) IPM School/Classroom Sham (Placebo) Air Filter, (3) Control School/ Classroom Air Filter and (4) Control School/Classroom Sham (Placebo) Air Filter. This is the same primary outcome used in the other NIAID funded Inner-City Asthma Consortium Studies[1,3].

The disease pattern that we anticipate (and have seen in SICAS-1) will be representative of that of most inner-city urban asthmatic children. Many have frequent nighttime awakenings with asthma symptoms, disrupted activities, and frequent use of rescue medication. Their disease takes its economic toll through loss of caregiver work days, and child absences or increased health care utilization that does not necessarily involve hospitalization or systemic steroid bursts. Our primary composite outcome "maximum symptom days" was used and validated as the primary outcome in the Inner-City Asthma[3,14,33,36] trial and other home-based clinical environmental intervention trials[1,15]. Symptom-days and health care utilization were both demonstrated to have economic relevance in cost-benefit analyses by the Inner-City Asthma trial[1,72,83].

**Table 1: Primary and Secondary Clinical Outcomes[1,5,72,83]**

| Primary Clinical Outcomes | Primary Indices |
|---|---|
| Maximum asthma symptom days in past two weeks | Maximum number of<br>1. Days with wheezing, tightness in the chest, or cough and/or<br>2. Nights with disturbed sleep as a result of asthma and/or<br>3. Days on which the child had to slow down or discontinue play activities because of asthma |
| **Secondary Clinical Outcomes** | **Primary Indices** |
| School Absences | Number of school days missed because of asthma/ 2 weeks |
| Health Care Utilization | Total asthma-related unscheduled visits (UVs) defined as sum of unscheduled clinic visits and emergency department visits, and asthma-related overnight hospitalizations /school year* |
| Composite Asthma Severity Index[5] | Day symptoms and albuterol use, night symptoms and albuterol use, controller treatment, lung function, and exacerbations (defined as systemic steroids for asthma )* |
| Pulmonary Function (PFT) | $FEV_1$; $FEV_1$/FVC*<br>[Secondary indices : (a) $FEF_{25-75}$.<br>(b) Bronchodllator responsiveness (% change in $FEV_1$ post albuterol)] |
| Degree of Exposure Reduction | Health Outcomes (Primary and Secondary) |

* Standardized core asthma outcomes from Asthma Outcomes Workshop[5,6]
**Health care use post randomization

### 3.3. Secondary Endpoint

Secondary outcomes specified in **Table 1** will be standardized according to the recent Asthma Outcomes Workshop guidelines[5,6]. They include school absences, Health Care Utilization; the Composite Asthma Severity Index PFT's and degree of exposure reduction. As demonstrated in our Cost Effectiveness Analysis Plan, a number of these outcomes can be used in estimating costs of the asthma morbidity and cost savings of the intervention.

**Pulmonary function:**

$FEV_1$,
$FEV_1$/FVC
FEF 25%-75%
percent change in $FEV_1$ after short-acting bronchodilator

**Mechanistic Endpoints:**

To explore whether sensitization or other asthma phenotypes defined at baseline modify which pathways or networks of genes undergo intervention-related changes in methylation or gene expression that, in turn, are associated with improvement in asthma outcomes.

**DNA/RNA Methylation/Transcriptomic Biomarkers.** DNA methylation and RNA gene expression will be measured as described below. Within pathways or networks of genes (pre-specified or discovered in unsupervised analyses) we will (a) determine how our interventions influence changes in airway cell methylation or gene expression and (b) evaluate how our intervention-associated changes in methylation and gene expression influence asthma outcomes. Finally, using mediation analysis, we will (c) estimate how much of the intervention effects on asthma symptoms occurs through methylation/expression changes[81,82]

Thus these measures can be considered to be intermediate outcomes, but the changes in these measures between baseline and near the end of the intervention are being considered both as outcomes of the interventions, as predictors of the clinical outcomes (e.g., symptoms/2-week period) and finally, as mediators of the relationship of the interventions to the clinical responses.

Precious specimens beyond our scope to analyze with funding from this proposal will be saved to provide opportunities for future testing of follow-up hypotheses.

Mechanistic endpoint is magnitude of changes in cell gene methylation or expression in pathways (and secondarily, in genes) relevant to airway function and asthma to determine if the effects of school/classroom-based environmental interventions on symptoms/other measures of asthma control occur through these changes. This will be done by evaluating within pathways or networks of genes (pre-specified or discovered in unsupervised analyses) we will (a) determine how our interventions influence changes in airway cell methylation or gene expression and (b) evaluate how our intervention-associated changes in methylation and gene expression influence asthma outcomes. Finally, using mediation analysis, we will (c) estimate how much of the intervention effects on asthma symptoms occur through methylation/expression changes

### 3.4. Exploratory Enpoint(s)

1. Days of rescue medication use/2 weeks;
2. Days of cough without a cold/2 weeks;
3. Days of exercise-induced asthma symptoms/2 weeks;
4. Nocturnal symptoms/2 weeks.
5. Days of slowed activity/2 weeks.
7. Asthma control, assessed by the Asthma Control Test
8. **Pulmonary inflammation,** assessed by exhaled nitric oxide (FeNO)

The following exploratory research questions will also be addressed:
(1) In the Intervention Group, does the degree of exposure reduction modify the effect of the intervention on asthma outcomes?

(2) Is there a multi-level effect in relation to School, Home, Class Effect on outcomes?

(3) In the Intervention group, does the presence of other indoor allergen sensitivities and exposures modify the effect of the intervention on asthma outcomes?

4) Does the degree of sensitization to mouse or mold (skin test size of allergen specific IgE level) modify the effect of the intervention?
Any of the above sensitization and exposure criteria
Other cut points will also be determined *a priori* and interactions will also be tested using exposure as a continuous variable based on the literature of clinically relevant exposure and sensitization levels.

5) Does obesity defined as Body Mass Index for age/percentiles modify these outcomes? This variable will be analyzed and adjusted for in models.

6) Does perceived stress defined as standardized perceived stress score[29] modify these outcomes? This variable will be analyzed and adjusted for in models.
7) Does sleep (habits,quality) affect any of these outcomes?
Validated comprehensive sleep questions and sleep diary will be collected.
Sleep quality assessed with wrist actigraphs (sleep monitors) which are worn for 5-7 days while sleeping.
Sleep quality assessed with sound level meter which will be placed in the home for 5-7 days.
Sleep Disordered Breathing will be assessed with the nasal oxygen cannula monitor and pulse oximeter.
These variable will be analyzed and adjusted for in models

8) Does pulse oximetry/ CO-Oximetry affect any of these outcomes? This variable will be analyzed and adjusted for in models

9) Are there any urinary biomarkers that predict outcomes? (i.e. metabolomics, proteomics, pesticides, urinary chemicals ( BP-3, BPA, Butylparaben, Ethylparaben, Methylparaben, Propylparaben, Triclosan, 2,4-dichlorophenol and 2,5-dichlorophenol, ispoprotenes, pesticides, benzenes, leukotrienes, and creatinine), that could effect outcomes

10) Are there any environmental metagenome, microbiome markers that could affect outcomes?

11) Are there any fungal, microbiome, bacterial markers that could affect or predict outcomes? (shot gun sequencing, ergosterol)

12) Would any food allergen exposures in the environment effect outcomes (i.e. peanut, milk, egg proteins)?

13) Does the intervention improve symptoms of other students in the classroom (i.e. non-allergic, nasal, irritant, other)?

14) Are there any other immune biomarkers which could further our understanding of mechanisms behind the response to our intervention such as pro-inflammatory cytokines such as IL-6, IL-33, leptin, basophils, TNF α, IFN-γ and other cytokines,

15) Are there gender differences that could modify our outcomes including serum gender specific hormones (i.e. E2,DHEAS,testosterone, SHBG, Progesterone)

16) Could certain nasal viruses, microbial organisms, and bacteria affect these outcomes?

17) Do Patient Reported Outcomes Measurement Information System (PROMIS) variables that measure patient–reported health status for physical, mental, and social well–being.affect our outcomes and are they reproducible in other cohorts of asthma and other chronic diseases?

18. Does the intervention prove to be cost-effective? (i.e. if it decreases missed school days and parent/caregiver missed days of work and improve quality of life in comparison to the cost of the intervention is it cost-effective?)?

19. Could any markers of ventilation, $CO_2$, temperature and humidity affect outcomes?

### 3.5. Stratification, Randomization, and Blinding/Masking

Randomization: Participants will be randomized within three weeks of the screening school visit in a 1:1 ratio using a statistical software package to generate random numbers. The randomization scheme will be developed by the DMAC. Participants at each site will be randomized independently of the other sites, and random blocks of 4 to 6 will be used to maintain similar enrollment into the two study groups at each site. There will be no stratification.

Masking: Although it may be possible to attempt to mask some study staff by increasing the number of research assistants at each site so that 1-2 RAs could be dedicated to collection of clinical outcome data, this would substantially increase the budget, so double-blind is not practical or feasible, even in a school-based study. Double-blinding in environmental home-based studies are also not feasible. There are also several aspects of the study that will guard against bias that could result from having unmasked study staff. First, all laboratory assays will be conducted by laboratory technicians who will be masked to group assignments. Second, some of the clinical data that are collected, including exhaled nitric oxide and pre- and post- bronchodilator spirometry, are objective measurements that are less subject to influence by the study staff or study participants. Third, analyses will be conducted to determine if any improvements in clinical outcomes are associated specifically with decreases in school allergen, mold pollutant levels, not just to group assignment.

The Air Filter Classroom Intervention, however, will be double-blind, placebo controlled randomization. Staff from the HSPH Environmental Assessment and Intervention Core who do not have contact with the study participants will service and change the air filters every 3 months at the school after school hours, maintaining participants, PI, and study staff blinding.

All laboratory studies (allergen ELISAs, allergen-specific IgE levels , endotoxin levels, PM 2.5, Black Carbon levels, $NO_2$ levels, and mold) will be performed in centralized laboratories in batches to minimize variability of the assay.

## 4. Selection of Participants and Clinical Sites/Laboratories

### 4.1. Rationale for Study Population

Health disparities urban school aged children is particularly high in regards to asthma and its associated morbidity, costing billions of dollars in health care utilization. Recent NIH/NIAID efforts have put significant resources in identifying remediable causes, particularly in the home environment.[8] Nearly all elementary school children spend 7 to 12 hours a day in school, and most of that time is spent in one classroom. It is nearly every child's required occupational environment. Our observational School Inner-City Asthma Study (SICAS-1) demonstrated the importance of the school environment in inner-city environments among elementary students. Therefore, focusing our population in elementary school age inner-city children makes this an ideal population for this study. Our proposed age/grade levels are a time where children spend the majority of their day in a primary classroom, and prior to further effect modifying changes that may change during puberty.

We are simply building on our established protocol and infrastructure developed during SICAS-1 and adding an established environmental school-based intervention to test our hypothesis**. This is an unprecedented, high impact opportunity to test whether we can efficiently benefit a community of children in the school environment as opposed to individuals in single homes.**

### 4.2. Inclusion Criteria

Individuals who meet all of the following criteria are eligible for enrollment as study participants:

1. Subject and/or parent guardian must be able to understand and provide informed consent
2. Males and females who are grades K-8 (age 4-15) during the subsequent academic school year after spring screening.
3. Attend one of the schools that study team have permission to obtain classroom/school environmental samples for the subsequent academic school year.
4. Have no plans to move schools within the upcoming 12 months
5. Have physician-diagnosed asthma at least 1 year prior to the screening visit
6. Have evidence of active, asthma as defined by at least one of the following:
    - Wheezing symptoms in the past 12 months
    - **On Daily controller medications for asthma**
    - One asthma-related unscheduled visit to an emergency department (ED), clinic or urgent care facility in the previous 12 months

- One asthma-related overnight hospitalization in the previous 12 months
- One or more bursts of oral or injectable corticosteroids in the previous 12 months

(criteria modeled from the NIAID funded Inner-City Asthma Consortium inclusion/exclusion criteria and SICAS) [1-4]

### 4.3. Exclusion Criteria for Participant

Individuals who meet any of these criteria are not eligible for enrollment as study participants:

1. Inability or unwillingness of a participant to give written informed consent or comply with study protocol Coordinator/PI judgment
2. Lung disease, other than asthma, that requires daily medication
3. Inability to do lung function testing
4. Cardiovascular disease that requires daily medication, excluding hypertension
5. Taking a beta-blocker
6. Currently receiving escalation (has not reached maintenance) of Immunotherapy (allergy shots)
7. Switching to a school where staff are not doing environmental sampling for that year.
8. Current, diagnosed, mental illness or current, diagnosed or self-reported drug or alcohol abuse that, in the opinion of the investigator, would interfere with the participant's ability to comply with study requirements

Past or current medical problems or findings from physical examination or laboratory testing that are not listed above, which, in the opinion of the investigator, may pose additional risks from participation in the study, may interfere with the participant's ability to comply with study requirements or that may impact the quality or interpretation of the data obtained from the study.

Exclusion Criteria for School

1. Unable to access areas of school necessary to conduct extermination
2. School in extensive state of disrepair/damage as determined by Study PI
3. Centralized Air Filtration or Purifying System ( although feasible to account for this, rare in schools)

### 4.4. Selection of Clinical Sites/Labs

The **Asthma Clinical Research Center (ACRC)** is run by Dr. Wanda Phipatanakul and was the single site that conducted all the clinical visits and recruitment for SICAS-1, and successfully recruited 400 elementary school children with the identical inclusion/exclusion criteria for SICAS-2. SICAS-1 entailed identical clinical procedures and a more aggressive milestone target than proposed for SICAS-2, ensuring the success of this Intervention protocol.

The **CTSU laboratory** is experienced in molecular biology procedures, immunological assays, radioimmunoassays, and specialized sample processing of various body fluids. Genetic testing and evaluation is also available as part of the Boston Children's Hospital Program for Genomics. As part of Harvard Catalyst, the study has access to all the CLIA approved laboratory facilities of **Boston Children's Hospital Clinical Laboratory** and of the entire Harvard Community including the **Harvard School of Public Health (HSPH) Laboratory of Human Epigenetics and Genetics,** the **Program in**

**Genomics,** the **Integrated Health Science Facility**, the **Harvard Partners Microarry Core and Columbia University** for the nasal epigenetic/expression mechanistic studies, and the **HSPH Environmental Chemistry Laboratory** for the analysis of environmental particulate matter and pollutants. **University of Iowa** will provide the analysis of the mold spore sampling and allergen/endotoxin analysis of the environmental dust and air samples. This entire infrastructure and set up has already been running in place successfully for SICAS-1.

## 5. Known and Potential Risks and Benefits to Participants

### 5.1. Risks of Investigational Product or Intervention as cited in Investigator Brochure or Package Insert

*Not Applicable: Environmental Intervention*

### 5.2. Risks of Investigational Product or Intervention cited in Medical Literature and/or those based on the Investigators' experience

Portable Room High Efficiency Particulate Air (HEPA) Air Purifier Filters are commercially available and provide no risk to the children as they are used to improve environmental air quality.

Integrated Pest Management may involve using caulking agents, traps, sealing plugging of holes and keep pests out of buildings as directed by manufacturer instructions.

Cleaning will be standard practices that are to improve the environment and as routinely done by janitors in a school setting. We plan to use non-bleach agents. Commercially available, non-bleach cleaning agents (e.g. 409®, Fantastik®) will be used. The risk of any irritation or symptoms is very small. The cleaning agents will be used as directed by the manufacturers as routinely done.

Commercially available, non-bleach cleaning agents (e.g. 409®, Fantastik®) will be used during the cleaning that occurs during the first IPM visit. Commercially available foam caulking agents (Pur-fill®) will be used to seal holes and cracks during the IPM visits. Both of these agents contain potential irritants that could cause asthma symptoms. The risk of developing asthma symptoms serious enough to require emergency medical attention is very small. The cleaning agents will be used as directed by the manufacturers.

### 5.3. Risks of Other Protocol Specified Medications

**Risks of Albuterol:** Albuterol is a medicine used routinely to help diagnose asthma and to treat asthma, and serious reactions are rare. The more common side effects are

- fast heart beat
- shakiness
- upset stomach
- heartburn
- nervousness

### 5.4. Risks of Study Procedures

**Skin Testing:**

There is a risk of

- Feeling of itching where the skin test was done,
- Red raised bump, like an insect bite, where the skin test was done
- Rarely wheezing or shortness of breath
- 1 in 10,000 tests results in a reaction such as sneezing, itchy eyes or hives
- Even more rarely, a reaction may occur that may be life-threatening, and in extremely rare cases may cause death

**Stopping Antihistamines:** If child is taking antihistamine medication, and stops taking it before skin testing, child may have more allergy symptoms such as sneezing, runny nose or stuffy nose.

**Blood Draw**: Taking blood may cause pain, bleeding, or bruising where the needle is placed.

- In rare cases, it may result in fainting.
- There is a small risk of infection.

**Spirometry**

- Light-headedness during the breathing out period.
- Occasionally minor chest soreness for several days after the testing.

**Exhaled Nitric Oxide**

- Your child may become lightheaded or dizzy but this is unlikely

**Pulse Oximetrry/**

- There are no known risks

**Pulse CO Oximetry**

- **There are no known risks**

**Sleep Monitor**

- The nasal sensor may cause slight discomfort i.e.tickling sensation in the nose).
- The actigraph worn on the wrist overnight may cause slight discomfort.

**Saliva**

- There is no known risk associated with spitting into a cup.

**Buccal brush Collection**

- There is no known risk associated with the collection of buccal cells using small, soft brush

**Urine Collection**

- There is no known risk associated with providing a urine sample

**Nasal swab/blow**

- There is no known risk with providing a nasal sample in this manner.

**Skin swab**

- **There is no known risk with providing a skin swab**

**Nasorption**

- There is no known risk with providing a nasal fluid sample in this manner

**Nasal Epithelial Cell Collection**

- Epistaxis
- Nose may feel a bit of tickling in the area or mild irritation.

### 5.5. Potential Benefits

Individual participant benefits : The potential benefits for the study participant includes receiving an assessment of allergic sensitivities, assessment of school environmental exposures, classroom air filters, extermination and cleaning, as well as education regarding environmental control practices. The study participants may also benefit from improved asthma control.

Benefits to Society : The potential benefits to others include the identification of a method to reduce asthma morbidity in students. This knowledge could impact policy decisions regarding environmental interventions to reduce school/classroom exposures that may trigger asthma symptoms. This information will directly impact the medical care provided to this patient population and could confirm the role of a school/classroom based environmental interventions that could effectively and efficiently benefit a community of children. *If we demonstrate that reduction of classroom-specific exposures leads to improved asthma outcomes, then our findings can be translated into efficient and cost-effective strategies to benefit communities of children through improving the school environment, where children in America spend the majority of their day.*

## 6. Intervention

### 6.1.1. Intervention #1

COWAY AP1013Amodel High Efficiency Particulate Air (HEPA) Air Purifier Filtration Unit- commercially available for use of air filtration in homes, buildings, and classrooms

#### 6.1.1.1. Formulation, Packaging, and Labeling

Four COWAY AP1013A model, room Air Filters will be deployed in each school randomized to the IPM Group (appropriate for rooms up to 170 square feet). Units will be deployed in the enrolled SICAS-2 student's primary classroom. Number of Units will be determined by the size of classroom. The units are available in commercial packaging that includes an instruction manual. The HSPH Intervention Core will do the masking and blinding of the Sham (Placebo) Air Filters and the Active Air Filters with a coded randomization for deployment. The study staff and investigators will be blinded to whether the Air Filters are Active or Sham. For details on commercially available air filters, used commercially in rooms for air filtration, company website (Ransport,New Jersey or Los Angeles, CA)

http://coway-usa.com/product/03_airpurifier.html

##### 6.1.1.2. Dosage, Preparation, and Administration

Air filter units will be stored at each site according to the manufacturer's recommendations and product label. Air filter units will be checked for functionality prior to deploying in a school.

#### 6.1.2. Intervention #2

Integrated Pest Management/Cleaning

##### 6.1.2.1. Formulation, Packaging, and Labeling

We are modeling methods off of published methods that work in Boston homes , which led to our NIAID funded Home based study (Mouse Allergen Asthma Intervention Trial U01AI083238), and which utilizes successful strategies reducing mouse allergen in homes.

In order to ensure the provision of high quality IPM services according to the study protocol, IPM company (Buono Pest) that already have been trained for the NIAID AI 083238 will work with the study teams. Buono Pest is licensed and insured and have been successfully working with us in the NIAID funded Mouse Allergen Asthma Intervention Trial. Buono Pest will provide approximately 3-4 IPM technicians who will undergo training for the SICAS-2 IPM protocol along with the IPM Company President/CEO and/or operations manager, whichever is most appropriate.

**Overview:** Each IPM Module will include two school visits. The first visit of each module will be conducted by a Research Assistant (RA) and an IPM technician (IT) from Buono Pest. The two IPM visits will be 4±3 weeks apart for the initial module that is delivered after randomization. Subsequent IPM modules will occur if there is evidence of infestation or report of infestation at the follow up school assessment visits, and will consist of two IPM visits that will be 4±3 weeks apart. The control group will receive one IPM intervention visit. IPM is further described in the study procedures section 8 after completion of the study.

### 6.2. Assessment of Participant Compliance with Investigational Agent

Not Applicable (Environmental Intervention)

### 6.3. Toxicity Prevention and Management

Not Applicable (Environmental Intervention)

### 6.4. Premature Discontinuation of Investigational Agent

Not Applicable (Environmental Intervention)

## 7. Other Medications

### 7.1. Concomitant Medications

#### 7.1.1. Protocol-mandated

Not Applicable(Environmental Intervention)

#### 7.1.2. Other permitted concomitant medications

Medications and/or procedures prescribed by the participant's health care provider or initiated and obtained by the participant's parent/guardian are permitted.

### 7.2. Prophylactic Medications

Not Applicable

### 7.3. Prohibited Medications

Taking a beta-blocker
Currently receiving escalation (has not reached maintenance) of Immunotherapy (allergy shots)
Antihistamines will be discontinued for 3-5 days prior to the screening clinic visit for skin testing.

### 7.4. Rescue Medications

Not Applicable.

## 8. Study Procedures

Clinical and Environmental Evaluations will occur during five types of study events: clinic visits, telephone questionnaires, school/home assessment visits, home assessment visits, and school subject follow-up visit.

### 8.1. Enrollment

We will utilize the same successful recruitment/enrollment strategy that we have been implementing in SICAS 1.

We will use, as we have done successfully in SICAS-1, validated screening surveys[84]( collected under a screening survey protocol) that were distributed to 8-10 elementary schools to be filled out by students and teachers. From SICAS-1, we have averaged 1000 to 1500 surveys returned each spring.

Potential study participants will be screened over the telephone and those who meet study criteria and fulfill inclusion/exclusion criteria by an eligibility screening form will be explained about the study and invited to be scheduled for a baseline enrollment study visit in the clinic.

### 8.2 Screening Visit

This visit will take place at the **Asthma Clinical Research Center (ACRC)** in order to confirm eligibility and phenotypically characterized participants' asthma before randomization at the start of the school year. The procedures performed will be:

- *Informed Consent***:** The research study will be explained in lay terms to each potential research participant. The participant's parent/guardian will sign an informed consent form and the participant will sign an assent form before undergoing any study procedures. Once the informed consent and assent have been signed, the participant is considered enrolled in the study and will be assigned a unique participant number.

- *Questionnaires* - assess clinical characteristics (asthma symptoms, health care utilization, home characteristics);
- *Anthropometric Measurements***:** Height, weight and waist will be taken.
- *Oximetry*: The saturation of oxygen will be obtained by placing an electric device in the finger.

- *CO oximetry* : The percentage carbon monoxide in the blood will be assessed by placing a small device in one of the fingers.
- *Skin testing:* Allergy skin testing will be performed to 14 allergens, using the MultiTest II device (Lincoln Diagnostics, Decatur, IL). The allergen extracts to be used are: Timothy grass, Penicillium, German Roach, Oak tree pollen, Dog, Cat, Mouse epithelia, Rat epithelia, *Dermatophagoides farinae*, *Dermatophagoides pteronyssinus* Der p, Aspergillus mix, Alternaria tenuis, Cladosporium, Ragweed pollen mix . Skin tests will be performed according to standard procedures[85]
- *Fractional Exhaled NO (FeNO): F* eNO is a known marker of pulmonary inflammation and will provide a non-invasive means of assessing pulmonary inflammation.. Measurement of exhaled nitric oxide will be obtained prior to lung function testing, and will be obtained according to the American Thoracic Society Guidelines[86]. Exhaled nitric oxide concentrations will be measured using an FDA-approved (for clinical use in asthma management) handheld device that uses electrochemical technology to quantify FeNO levels (NIOX Mino/NIOX Vero System, Aerocrine, Sweden). .
- *Spirometry and Bronchodilator reversibility:* Pre- and post-bronchodilator spirometry will be performed according to ATS guidelines[87]. At least three reproducible flow-volume loops will be obtained using the portable Koko spirometer, after which albuterol will be administered via nebulizer. Approximately 10-15 minutes after completing the nebulized albuterol, spirometry will be repeated to obtain post-bronchodilator FVC, FEV1, FEF25-75, and PEF.
- *Nasal swab/blow:* This will be obtained by swabbing the nares to clean and blowing into a nasal sample kit to be frozen and saved for future analysis of viral and microbial organisms that could affect clinical response to the intervention.
- *Skin swab:*_ This will be obtained by swabbing the crease behind the ear with a damp swab. This sample will be frozen and saved for future analysis of viral and microbial organisms that could affect clinical response to the intervention.
- *Nasorption:* This will be obtained by placing a small piece of absorbent paper in one of the nares of the child's nose and staff will hold that side close for a few seconds. These samples will be stored and may be used for future understanding of inflammatory markers linked with asthma or environmental changes
- *Nasal brushing (optional):* Only children who have agreed to this procedure We will collect nasal epithelial cells from behind the inferior turbinate. The procedure will be done in kids 10 and older using a cytology brush under direct visualization with a nasal illuminator. This procedure will be done one time before the start of school and one time before the child finishes the school year. The procedure will only be done in the hospital clinic. The collected brush will be submerged in RLT Plus lysis buffer plus b-mercaptoethanol and frozen at -80 degrees C until extraction. Samples will be stored at Baccarelli's Laboratory.
- *Venipuncture* A 35 ml venous blood sample will be obtained so that the serum can be used to measure total IgE and allergen-specific IgE levels. Cell count with differential to obtain eosinophils will also be obtained. The leftover serum and plasma will be frozen at Biocore (BCH)for future research use.
  *Urine Collection:* A sample of 25 ml of urine will be collected and Nic-Alert/cotinine and specific gravity measure done. An aliquot of urine from the screening visit will be saved for measurement of F-isoprostane, pesticides and other inflammatory markers. Additional aliquots will be saved and frozen in the biorepository for future use.

- *Saliva*: This sample will be used to measure cortisol levels as a marker for stress. Children will chew on parafilm paper for up to 15 minutes or until 6-10ml of saliva are collected. The sample will be aliquoted. They will be sent to be frozen and stored in the biorepository for future use should funding become available.
- *Buccal Swabs(optional):* This sample will be collected to look into DNA/RNA epigenetic changes due to environmental exposure. We will use a cytology brush (6 brushes) to get the sample from the inside of the cheeks and lips. This sample will be frozen and saved in the biorepository for future use should funding become available.
- *Sleep monitor (optional):* There are 3 parts of this evaluation.

a) Evaluation of sleep disordered breathing: The subject will wear a home sleep testing device that is FDA approved to safely monitor sleep in children down to age 2. There are three sensors 1) a pneumatic sensor attached to a Velcro belt measures respiratory effort. 2) a nasal cannula records airflow and snoring and 3) a pulse oximeter measures pulse and oxygen saturation. The device will be worn for 2 nights, only while subject is asleep.

**b)** Evaluation of sleep habits and quality: The wrist actigraph is worn like a wrist watch and records movements during day and night. The wrist actigraph will be worn for a total of 7 days (+/-2). A sleep diary will be completed while the subject wears the actigraph.

**c)** Home noise evaluation: To measure the noise inside and outside the home, we will ask the participant to place a sound meter. This is a machine that will be placed in the child's bedroom and will record the amount of noise in this space

If a subject cannot complete all activities at one screening visit, he/she may return within a week to complete the remainder of study activities.

8.3 **Home Environmental Assessment:** This assessment will done once at any point throughout the subject's participation in the study. Dust sampling collection from the participant's bedroom and kitchen. Home samples will be linked to school/class sampling as a surrogate measure for home exposure and for adjusted analyses. Home environmental assessments will also be done to differentiate the effect of home exposure in adjusted analyses with the primary focus of this study on the school/classroom specific exposure and clinical outcomes.

**8.4 Randomization**. The DCC Data Management System will randomize the classroom to receive active versus sham (placebo) air purifiers and the school to receive the IPM intervention. Randomization will occur by site and with random blocks. The randomization scheme will be developed by the Data Coordinating Center (DCC) and embedded into the data management system so that schools and classrooms can be randomized, linked to enrolled SICAS-2 students. Study staff will not have access to the randomization codes.

**8.5 Integrated Pest Management (IPM)**

Intervention includes IPM Module of two IPM visits, conducted 4 ±3 weeks apart.

**IPM Intervention Visit 1**: If the School has been randomized to the IPM School, the study coordinator will schedule an IPM visit in the fall after the baseline school sampling. The IPM intervention team will include the Research Assistants (RA)

and IPM Technicians (IT). The Classroom/School Inspection Form(s) will be used to document a walk through the school in order to sketch a layout and document visual evidence of infestation and holes/cracks in the structure. IPM procedures will include placement of traps, sealing of holes and cracks as specified in the MOP.
Our school-based work will focus on the primary exposure school room of interest, the child's primary home classroom, which is where we focused in our observational findings that provided supporting data for this study. We 1) inspect conditions, record observations, 2) Identify pests, 3) continue follow up and adjust as needed, 4) record observations and activities, 5) educate staff. Focusing on the classroom/cafeteria will also allow us to isolate and control conditions focused where the child spends the majority of his/her time. We will also clean and target IPM on the heating system of the classroom, and evaluate nearby surrounding areas, including cafeteria, and any nearby support areas if they directly feed into the classroom such as storage rooms, boiler rooms, janitor/storage closets if they have evidence of mouse infestation.

**IPM Intervention Visit 2**: The second visit of the IPM module will serve as a booster visit and will include an assessment of the status of the mouse infestation and repeat setting of traps, and sealing of holes and cracks.

**Additional IPM Intervention Visits**: Subsequent IPM modules will be delivered only if there is persistent or recurrent mouse infestation so that IPM will be tailored to the infestation status of the school. Mouse infestation will be assessed at the follow up school visits and a school will be considered to have ongoing or recurrent mouse infestation if there is evidence of mouse infestation during inspection or if staff reports seeing mice, or evidence of mice, during subsequent sampling.

**IPM Accountability:** Several procedures will be in place to ensure adherence to the protocol, including timely and appropriate delivery of IPM services. These procedures include:
1. IPM technicians will undergo training and certification prior to enrollment. The training and certification will be led by Certified Pest Management QI .
2. IPM technicians will be required to complete all study forms and document procedures; forms will be reviewed by the Study Coordinator and 10% will be reviewed by Pest Management Specialist annually
4. Annual site visits will be conducted by Pest Management Quality Control Specialist to observe at least 1 IPM visit to the schools.
5. IPM Company President or Operations Manager will participate in regular conference calls to ensure timely communication of any protocol deviations or issues that need to be addressed.
6. For IPM visits, the visits and dates of the visits will be documented. In addition, the IPM Technician(s) will document the number of traps placed.

**8.6 School Environmental Assessment:** Participating schools for the Academic Year will be scheduled for a baseline school visit to determine allergen/mold/particulate exposure status in the fall prior to deploying the randomized intervention. At the school visit, a school environmental assessment will be conducted, and settled dust and table wipes will be collected from SICAS-2 student's home classroom, cafeteria , and gym, The air samples will be collected from the student's home classroom to have a baseline determination of airborne dust. School environmental assessment includes the following procedures:

Completion of School Assessment/Inspection Form

- Collection of settled dust from cafeteria, primary home classroom and gym.

- Air pollution, air and mold samples will be collected from the primary home classroom.

The dust and air samples from home and school will be shipped to Indoor Biotechnologies for allergen (dust mite, mouse, rat, cockroach, cat, dog, alternaria, milk, peanut, egg)/endotoxin analysis. The mold samples will be analyzed at the University of Massachusetts, Amherst. Particulate Pollutant monitoring samples will be done at HSPH .

A return school visit will occur approximately one week after the first school visit to collect the air sampling equipment and samples. Follow-up school environmental assessments will take place once during the fall school semester and one during the spring school semester.

**8.7 Phone call surveys:** These will be performed in order to collect health outcomes (e.g., symptoms, health care use; time-activity) and home environmental information. They will be conducted after the start of the school year (baseline), and every 2 months after the intervention is deployed, yielding 4 follow-up measures during the school year, including 1 at the end of the school year.

**8.8 School Subject Follow-up visit**: By the same standard procedures performed at the screening visit, we will obtain anthropometric measurements (height and weight), Co-oximetry measurement, spirometry, FeNO, at the fall and spring follow-up visits. Nasal swab/blow, skin swab, nasorption and buccal swabs will only be performed during the spring follow-up visit. Follow-up clinical assessments will be linked in timing to the environmental sampling done during the academic year, post randomization. Not only will the timing of the assessments enable us to evaluate the effects of the interventions on these outcomes (lung function, FeNO,) partway through and near the end of the intervention periods. This timing will also enable us to do secondary analyses on the relation of these outcomes to short-term measured school exposures in the different treatment arms.

These school environmental and clinical measures will enable us to: (1) estimate the efficacy of the interventions in opposite seasons and (2) conduct secondary analyses linking season-specific health outcomes with exposure measures rather than treatment group.

**8.9 Focused Cleaning:**

At the firs visit of the first IPM module, the research assistant will complete a focused cleaning aimed at (1) removing allergen reservoirs; and (2) removing clutter to aid the IPM technician. Procedures will include removal of dead mice from traps, removal of trash and clutter, removal of mouse droppings, wet mopping of floors with hard surfaces and vacuuming carpeted floors with HEPA filtered vacuum cleaner. Our methods are modeled off of successful intervention work in Boston homes[18] which led to our NIAID funded Home based study (Mouse Allergen Asthma Intervention Trial U01AI083238), and which utilizes successful strategies targeted to the bedroom, inspection targeted in surrounding areas, and kitchen. Our school-based study will focus on the primary exposure school room of interest, the child's classroom, surrounding support areas, and the cafeteria.

**8.10 Education:**

An educational module will be delivered to IPM Schools. The Education SOP which can be found in the MOP details the information to be covered and the Education Form will serve as documentation of delivery of the Education Module.

The module will review the approaches to reducing mouse allergen levels – source removal, prevention of re-entry, and cleaning of allergen reservoirs with school staff.

8.11 HEPA Air Filter Accountability

According to the manufacturer, the portable air filter units have a particle collection efficiency of 99.9% for particles as small as 0.3 microns. All filters will be replaced at each school visit, every 3 months, as recommended by the manufacturer's instructions.

For the Classroom Air Filters, a research assistant will assess the units at the school visits to document functionality and whether the unit is on and plugged in at the time of the school visit. Reminders are taped on the units to encourage compliance. If the units are turned off or unplugged, staff will communicate with the school contacts to encourage compliance.

8 . 1 2 **Unscheduled Visits**

There will not be unscheduled visits since the study will not be managing the study participant's asthma. Instead, documentation of the study participant's primary care provider will occur at the screening clinic visit and participants who do not identify a primary care provider will be referred to one. A participant who contacts study staff with acute health concerns will be referred to his/her primary care provider or the Emergency Department, depending on nursing or physician judgment.

**8.13 Laboratory Reference Ranges:** We will use age and sex specific published standard reference ranges for all clinical labs to define out of range safety lab values in the clinical database. Laboratory results will also be reviewed by the PI or her physician investigator designee as they become available to follow-up promptly with the family regarding any abnormal results that require medical intervention. The original lab results reports for each participant will be signed and data to verify they have been reviewed and action taken where appropriate.

**Schedule of Events:**
**Table 2 Surveys**

| Measure | Pre-Screening<br>Pre-baseline<br>(Spring) | Screening<br>(Summer) | Baseline Survey Fall | Randomization | 2 mo Follow up<br>Fall-Winter | 4 mo Follow up<br>Winter-Spring | 6 mo Follow up<br>Spring | Final Survey<br>End of School |
|---|---|---|---|---|---|---|---|---|
| Student Screening Survey | X | | | | | | | |
| Eligibility Form | X | | | | | | | |
| Baseline Questionnaires<br>*Primary outcome Ascertained | | X | X | | | | | |
| Follow-up Questionnaires<br>* Primary outcome Ascertained | | | | | X | X | X | X |
| Safety Survey (identified through health outcome surveys) | | | | | X | X | X | X |

| Table 3 Clinical Procedures | Screening<br>(Jun-Sep) | Randomization | School Follow-Up 1<br>(Dec-Feb) | School Follow-Up 2<br>(Mar-Jun) |
|---|---|---|---|---|
| Participant Baseline Visit Data and Sample Collection<br>• CBC/Allergen specific IgE<br>• Serum, plasma, urine and saliva for storage<br>• Nasal swab and blow<br>• Skin swab<br>• nasoprtion<br>• Buccal Swab (optional)<br>• Height, Weight, Heart rate, Pulse Oximetry | X | | | |

| | | | | |
|---|---|---|---|---|
| • Skin Allergy Test<br>• Pre/Post Albuterol Spirometry<br>• Nasal Brush (optional) | | | | |
| Participant Follow-up Visit Data Collection<br><br>• Spirometry<br>• FeNO<br>• CO-oximetry | **X** | | **X** | **X** |
| Participant Follow-up Visit Sample Collection at School<br><br>• Nasal Swab and Blow<br>• Buccal Swab<br>• Skin swab<br>• nasorption | | | | **X** |
| Participant Follow-up Clinic Visit Sample Collection<br><br>• Nasal Brush (optional) done in clinic only | | | **<----------** | **----------- >** |

**Table 4: Environmental Measures and Forms (School, Classroom, and Home)**

| Measure | Baseline (Oct-Nov) | Randomization | Follow-Up 1 (Dec-Feb) | Follow-Up 2 (Mar-Jun) |
|---|---|---|---|---|
| School Contact and Demographics Form | X | | | |
| School/Classroom Evaluation | X | | X | X |
| School Environmental Samples | X | | X | X |
| Home Environmental Samples- Vacuum Dust** | <----------- | | -------------- | ----------- > |

****One home dust sample is taken at any time during the year as a surrogate measure for home exposure**

## 9. Mechanistic Assays

**<u>Rationale:</u>**

**<u>Mechanistic Background Data Methylation and gene expression to environmental responses</u>** Epithelial cells (ECs)[93, 94] provide the front-line physical, immune, and chemical barrier to the allergen and irritant exposures that we will reduce[88,8988,8982, 89]. Pattern recognition receptors (PRRs: TLRs, NOD-like receptors, C-type lectins; protease-activated receptors) on ECs recognize proteolytic allergens and allergen-associated microbial contaminants, with EC release of innate cytokines (e.g., TSLP,CM-CSF, IL-25, IL-33) that can activate dendritic cells and increase Th2 cytokine expression, or activate basophils, mast cells and eosinophils involved in allergic/asthmatic responses. Altered EC barrier function with tight-junction disruption is linked to poor ciliary clearance or increased mucous production in asthma. EC innate[96,9790,9190,91], barrier, and repair function may be dysregulated by particle pollution components as well as cigarette smoke[98].

Gene expression is regulated in part by upstream epigenetic programming (e.g., by DNA methylation, as well as by a coordinated suite of less accessible epigenetic marks acting in synergy with DNA methylation). Both can be influenced by environmental exposures,[92-96] as well as by genetics. We have shown that within-person methylation of PRRs (TLR4) is decreased with particle pollution exposure[97]. We found higher airway inflammation (FENO) was associated with methylation of the innate pro-inflammatory cytokine IL-6 (29.0%; p=0.004) and iNOS in asthmatic children[98]. While epigenetic silencing of some genes may be established in utero, we have demonstrated plasticity in methylation of other genes, with changes that begin with short-term exposures and become greater with cumulative exposure over months[99-107].

**<u>I Mechanistic Assays</u>*<u>:</u>*** This would be the first trial assessing how school-based interventions (integrated pest management and/or reduction in classroom particles levels) influence changes in methylation and downstream gene expression pathways or networks of genes that, in turn, have relevance to reduction of asthma morbidity and improvement in the natural course of the disease. Application of bioinformatic techniques will enable us to discover pre-specified as well as novel pathways of genes that respond to our intervention with changes in methylation and/or

changes in gene expression relevant to prevention of asthma morbidity. Thus ascertainment of the benefits of our environmental intervention at the molecular level will help to identify novel pathways to target in pharmacologic therapy for children with asthma, and to explore whether pathway responses vary by asthma phenotype or by baseline gene expression and methylation. Furthermore, our study has clinical applicability, yielding measures with direct relation to airway biomarkers[83, 114108,109108,109], utilizing samples that can be obtained from children non-invasively, rather than by more invasive procedures like lung biopsy.

**Health Outcomes from Students in Entire Classrooms (Exploratory):**

Secondarily, depending on scheduling with the school, we will redistribute surveys to entire classrooms of the same classrooms/schools that we are collecting environmental dust/air sampling data during the academic year. This will allow us to have a one follow up measure of health outcomes form the entire classrooms, linked to the environmental sampling. This will provide us with symptoms (including nasal) from the students without asthma that are enrolled in the study.

This allows to efficiently obtain health outcome data of our intervention in entire classrooms of these students. In SICAS-1, most of classrooms of students in our study had at most 2-3 students in a classroom of 20 students. Although these outcomes are not the primary specific aims of SICAS-2, this will allow us to assess preliminary respiratory and nasal symptoms from entire schools, linked to our intervention and preliminarily determine the intervention on 10-20 times the number of enrolled SICAS-2 students with asthma or 3000-6000 students, and greatly leverage the wealth of information on determining the effectiveness of our intervention in a community of students.

## 10. Biospecimen Storage

Serum, plasma, saliva, urine and buccal brushes, nasal swab and blow samples will be stored using unique identifiers. These samples will be stored at BIOCORE part of Boston Children's Hospital for possible future use. Samples will be saved for future analyses such as immune markers such as pro-inflammatory cytokines such as IL-6, IL-33, leptin, basophils, TNF α, IFN-γ and other cytokines, stress markers such as salivary cortisol and α-amylase, serum gender specific hormones (i.e. E2,DHEAS,testosterone, SHBG, Progesterone) urinary metabolomics, proteomics, urinary chemicals ( BP-3, BPA, Butylparaben, Ethylparaben, Methylparaben, Propylparaben, Triclosan, 2,4-dichlorophenol and 2,5-dichlorophenol, ispoprotenes, pesticides, benzenes, leukotrienes, and creatinine), and buccal brushes saved for epigenetics/RNA gene expression, and nasal swab/blow samples saved for viral, microbial, and bacterial organism analysis, which may help us further our understanding of mechanisms behind the interventions, pending future funding

## 11. Criteria for Participant and Study Completion and Premature Study Termination

### 11.1. Participant Completion

Subject who has completed summer baseline clinical and clinical follow up through the entire subsequent academic school year has completed the study as long as they remain in the school classroom where the intervention and

environmental sampling is also done subsequently. Required milestones include minimum clinical baseline evaluation and clinical outcomes post randomization of intervention linked to classroom exposure sampling.

### 11.2. Participant Stopping Rules and Withdrawal Criteria

Participants may be prematurely terminated from the study for the following reasons:

1. Voluntary withdrawal by the study participant may occur at any point during the course of the study. Every effort will be made to collect clinical and AE data via telephone.
2. The participant is "lost to follow-up" (i.e., no further follow-up is possible because attempts to reestablish contact with the participant have failed). After up to 10 attempts at follow-up by through varying methods, phone, email, letters[110], which is the commonly required for follow up in this population to maintain retentions, we will assume patient is lost to follow up.
3. The participant dies.
4. A study participant will be discontinued from further study intervention if any clinical adverse event, other medical condition or situation occurs such that continued participation in the study would not be in the best interest of the participant. This includes an asthma hospitalization that results in intubation.
5. The intervention is determined to be too disruptive in the classroom or school and the school or classroom staff request withdrawal from the study.

### 11.3. Participant Replacement

Study participants who have completed one follow up clinical visit after the school/classroom has been randomized to the environmental intervention will not be replaced. Participants who withdraw or are withdrawn will not be replaced.

### 11.4. Follow-up after Early Study Withdrawal

Voluntary withdrawal by the study participant may occur at any point during the course of the study. Every effort will be made to collect clinical and AE data via telephone.

### 11.5. Study Stopping Rules

Given the nature of this intervention and the enrollment schedule, we will not have a priori study stopping rules for early efficacy or futility. If we learn of new information that would make the intervention unsafe (unlikely in an environmental intervention study, but i.e. a severe adverse event directly related to the intervention), that could also warrant stopping the study if determined necessary by the DSMB.

## 12. Overview

This section defines the types of safety data that will be collected under this protocol and outlines the procedures for appropriately collecting, grading, recording, and reporting those data. Adverse events that are classified as serious according to the definition of health authorities must be reported promptly (per Section 12.5, Reporting of Serious Adverse Events and Adverse Events) to the sponsor DAIT/NIAID. Appropriate notifications will also be made to site PI, Institutional Review Boards (IRBs), and health authorities.
Information in this section complies with ICH Guideline E2A: Clinical Safety Data Management: Definitions and Standards for Expedited Reporting, ICH Guideline E-6: Guideline for Good Clinical Practice, 21CFR Parts 312 and 320, and applies

the standards set forth in the National Cancer Institute (NCI), Common Terminology Criteria for Adverse Events (CTCAE), Version [enter 4.0 or correct version applicable to trial] : http://ctep.cancer.gov/reporting/ctc.html.

## 12.1 Definitions

### 12.1.1 Serious Adverse Event (SAE)

An adverse event or suspected adverse reaction is considered "serious" if, in the view of either the investigator or Sponsor( DAIT/NIAID), it results in any of the following outcomes (21 CFR 312.32(a)):

1. Death.
2. A life-threatening event: An AE or SAR is considered "life-threatening" if, in the view of either the investigator or Sponsor( DAIT/NIAID), its occurrence places the subject at immediate risk of death. It does not include an AE or SAR that, had it occurred in a more severe form, might have caused death.
3. Inpatient hospitalization or prolongation of existing hospitalization.
4. Persistent or significant incapacity or substantial disruption of the ability to conduct normal life functions.
5. Congenital anomaly or birth defect.
6. Important medical events that may not result in death, be life threatening, or require hospitalization may be considered serious when, based upon appropriate medical judgment, they may jeopardize the subject and may require medical or surgical intervention to prevent one of the outcomes listed above.

**Asthma hospitalizations not related to study procedures will not be considered SAEs and will be entered on the appropriate Case Report Forms.**

### 12.1.2 Adverse Event (AE)

Any untoward or unfavorable medical occurrence associated with the subject's participation in the research, whether or not considered related to the subject's participation in the research (modified from the definition of adverse events in the 1996 International Conference on Harmonization E-6 Guidelines for Good Clinical Practice) (from OHRP "Guidance on Reviewing and Reporting Unanticipated Problems Involving Risks to Subjects or Others and Adverse Events (1/15/07)" http://www.hhs.gov/ohrp/policy/advevntguid.html#Q2 )

For this study, recorded and reported as an adverse events are limited to 1.) any occurrence or worsening of an undesirable or unintended sign (including an abnormal laboratory finding), symptom, or disease in a study subject that is specifically associated (possible, or definite – defined in section 10.2.2.) with a study procedure; 2) Asthma exacerbations that require hospitalization and are determined to be related to study procedures will be considered a SAE and 3) SAEs which are not asthma exacerbations.

**Study therapy regimen**: Environmental Intervention, generally not applicable. No treatment given as part of this study.

- **Study mandated procedures**: blood draw, nasal sample, skin swab, nasorption and lung function test. For the procedures below, clinical situations are listed that are considered to be outside the normal range of outcomes and will be recorded as Adverse Events. These situations do not limit an investigator from recording and reporting any other events, associated or not with these procedures as AEs.

Allergen Skin Testing

- Prolonged (>24 hours) itching at test site
- Swelling (> 10 cm ) at site of test lasting more than 24 hours
- Nasal allergic symptoms within 30 minutes from the procedure
- Fainting /Vasovagal event within 30 minutes from the procedure

Blood Draw

- Fainting/Vasovagal events
- Bruising at puncture site larger than 2 cm diameter
- Bleeding from puncture site lasting more than 30 minutes
- Swelling at puncture site larger than 2 cm

**Pulmonary Function Testing**

- Wheezing or bronchoconstriction requiring treatment with bronchodilators within 30 minutes from the procedure
- Coughing requiring treatment with bronchodilators within 30 minutes from the procedure

**Nasal Epithelial Cell Collection**

- Epistaxis within 24 hours after the procedure

#### 12.2.1.1 Suspected Adverse Reaction (SAR)

N/A

### 12.2.2 Unexpected Adverse Event

Not Applicable.

### 12.2.1 Grading Criteria

The study site will grade the severity of adverse events experienced by the study subjects according to the criteria set forth in the National Cancer Institute's Common Terminology Criteria for Adverse Events (CTCAE) version 4.0. This document (referred to herein as the NCI-CTCAE manual) provides a common language to describe levels of severity, to analyze and interpret data, and to articulate the clinical significance of all adverse events. The NCI-CTCAE has been reviewed by the Principal Investigator and has been deemed appropriate for the subject population to be studied in this protocol.

Adverse events will be graded on a scale from 1 to 5 according to the following standards in the NCI-CTCAE manual:

Grade 1 = mild adverse event.

Grade 2 = moderate adverse event.

Grade 3 = severe and undesirable adverse event.

Grade 4 = life-threatening or disabling adverse event.

Grade 5 = death.

All AE's related to study procedures will be recorded on the appropriate AE case report form  for this study.

For grading an abnormal value or result of a clinical or laboratory evaluation (including, but not limited to, a radiograph, an ultrasound, an electrocardiogram etc.), a treatment-emergent adverse event is defined as an increase in grade from baseline or from the last post-baseline value that doesn't meet grading criteria. Changes in grade from screening to baseline will also be recorded as adverse events, but are not treatment-emergent. If a specific event or result from a given clinical or laboratory evaluation is not included in the NCI-CTCAE manual, then an abnormal result would be considered an adverse event if changes in therapy or monitoring are implemented as a result of the event/result.

### 12.2.2 Attribution Definitions

The relationship, or attribution, of an adverse event to the  study procedure(s) will initially be determined by the site investigator and recorded on the appropriate AE case report form AE/SAE paper CRF.  Final determination of attribution for safety reporting will be determined by DAIT/NIAID Medical Monitor (MM).  The relationship of an adverse event to study therapy regimen or procedures will be determined using the descriptors and definitions provided in Table 12.2.2.

For additional information and a printable version of the NCI-CTCAE manual, consult the NCI-CTCAE web site: http://ctep.cancer.gov/reporting/ctc.html

**Table 12.2.2 Attribution of Adverse Events**

| Code | Descriptor | Relationship (to study procedure) |
|---|---|---|
| **UNRELATED CATEGORY** | | |
| 1 | Unrelated | There is insufficient evidence to suggest a causal relationship |
| **RELATED CATEGORIES** | | |
| 2 | Possible | The adverse event has a <u>reasonable possibility</u> to be related; there is evidence to suggest a causal relationship. |
| 3 | Definite | The adverse event is clearly related. |

## 12.4 Collection and Recording of Adverse Events

### 12.4.1 Collection Period

Adverse events will be collected from the time of enrollment until a subject completes study participation or until 30 days after he/she prematurely withdraws (without withdrawing consent) or is withdrawn from the study.

#### 12.4.2 Collecting Adverse Events

Adverse events (including SAEs) may be discovered through any of these methods:

- Observing the subject.
- Interviewing the subject [e.g., using a checklist, structured questioning, diary, etc.] .
- Receiving an unsolicited complaint from the subject.
- In addition, an abnormal value or result from a clinical or laboratory evaluation can also indicate an adverse event, as defined in Section 12.2, *Grading and Attribution of Adverse Events.*

#### 12.4.3 Recording Adverse Events

Throughout the study, the investigator will record adverse events and serious adverse events as described previously (Section 12.2, *Definitions)* on the appropriate case report form AE/SAE paper CRF regardless of the relationship to study therapy regimen or study procedure.

Once recorded, an AE/SAE will be followed until it resolves with or without sequelae, or until the end of study participation, or until 30 days after the subject prematurely withdraws (without withdrawing consent)/or is withdrawn from the study, whichever occurs first.

### 12.5 Reporting of Serious Adverse Events and Adverse Events

#### 12.5.1 Reporting of Serious Adverse Events to Sponsor (DAIT/NIAID)

This section describes the responsibilities of the site investigator to report serious adverse events to the sponsor (DAIT/NIAID) via *(*Case Report Form, facsimile, hotline-mail)*.* Timely reporting of adverse events is required by 21 CFR and ICH E6 guidelines.

Site investigators will report to the NIAID Medical Monitor all serious adverse events (see Section 12.1.3, *Serious Adverse Event)*, regardless of relationship or expectedness within 24 hours of discovering the event.

Contact information for NIAID Medical Monitor:

Peter Gergen, MD, MPH
Division of Allergy, Immunology, and Transplantation – NIAID/NIH
5601 Fishers Lane
Bethesda, MD 20892-9828, USA
Phone: 240-627-3545
Fax: 301-480-4258
E-mail: pgergen@niaid.nih.gov*]*

For serious adverse events, all requested information on the AE/SAE Case Report Form will be provided. However, unavailable details of the event will not delay submission of the known information. As additional details become available, the AE/SAE case report form will be updated and submitted.

#### 12.5.1.1 Reporting of Unexpected Non-Serious Adverse Events

Any non-serious adverse event that is of Grade 2 severity or higher **and** study related will be recorded and reported to the NIAID Medical Monitor under the serious adverse event reporting procedure above (i.e. within 24 hours).

### 12.5.2 Reporting to Health Authority

Not Applicable

### 12.5.3 Reporting of Adverse Events to IRBs

All investigators shall report adverse events, including expedited reports, in a timely fashion to their respective IRBs/IECs in accordance with applicable regulations and guidelines.

## 12.7 Reporting of Unanticipated Safety Information

An investigator shall promptly notify the site IRB as well as the DAIT/Medical Monitor and Data Management and Analysis Core when an "unanticipated problem involving risks to subjects or others" is identified, which is not otherwise reportable as an adverse event.

## 12.8 Review of Safety Information

### 12.8.1 Medical Monitor Review

The NIAID Medical Monitor shall receive quarterly reports from the PI/Protocol Chair compiling new and accumulating information on AEs and , SAEsrecorded by the study site on appropriate case report forms.

In addition, the NIAID Medical Monitor shall review and make decisions on the disposition of the SAE received by the PI and Data Management and Analysis Core (See Sections 12.5.1, *Reporting of Serious Adverse Events to Sponsor (DAIT/NIAID)*).

### 12.8.2 Data and Safety Monitoring Board(DDSMB) Review

#### 12.8.2.1 Planned Data and Safety Monitoring Reviews

The Data and Safety Monitoring Board (DSMB) shall review safety data at least yearly during planned DSMB Data Review Meetings. Data for the planned safety reviews will include, at a minimum, a listing of all reported AEs and SAEs.

#### 12.8.2.2 *Ad hoc* DSMB Reviews

In addition to the pre-scheduled data reviews and planned safety monitoring, the DSMB may be called upon for *ad hoc* reviews. The DSMB will review any event that potentially impacts safety at the request of the Protocol Chair or the NIAID Medical Monitor. In addition, the following events will trigger an *ad hoc* comprehensive DSMB Safety Review:

- Any death that occurs in the study, which is possibly or definitely related to study treatment regimen.
- The occurrence of a Grade 3 or higher related and unexpected SAE in 3or more of the study participants who have received a study treatment.

- After review of the data, the DSMB will make recommendations regarding study conduct and/or continuation.

**12.8.2.2.1 Temporary Suspension of enrollment for *ad hoc* DSMB Safety Review**

A temporary halt in enrollment will be implemented if an *ad hoc* DSMB safety review is required. This is very unlikely in an environmental intervention, and study will proceed unless it is determined by DSMB that study must stop due to safety reasons.

# 13. Statistical Considerations and Analytical Plan

## 13.1 Overview

**Note:** SICAS-1 (**S**chool **I**nner-**C**ity **A**sthma **S**tudy) refers to observational R01 (AI 0739654, Phipatanakul), and SICAS-2 (**S**chool **I**nner-**C**ity **A**sthma Intervention **S**tudy) is the proposed clinical trial.

The primary objective of SICAS-2 is to test the efficacy of School Integrated Pest Management (IPM) and Classroom Air Filters in reducing asthma morbidity in school children with asthma. Reducing school/classroom mouse, mold allergen and particulate pollutant exposure will be associated with improvement in asthma outcomes.

Our mechanistic objective is to test the hypothesis that effects of school/classroom-based environmental interventions on symptoms/other measures of asthma control occur through changes in gene methylation or expression in pathways (and secondarily, in genes) relevant to airway function (Research Strategy Section **1.4**) and asthma.

## 13.2 Outcomes (Described in 1.1-1.3)

**Primary Outcome(s)**

## 13.3 Measures to Minimize Bias

*Randomization:* Schools will be randomized to IPM vs no IPM, and within schools, participant classrooms will be randomized to HEPA filter vs Sham Air Cleaner within three weeks of the baseline school visit in a 1:1 ratio using a statistical software package to generate random numbers. The randomization scheme will be developed by the Data Management and Analysis Core (DMAC). Dr. Stravoula Osganian, MD, MPH, DrPH and her team will lead the DMAC and assistance from Dr. Jonathan Bickel's team from the Clinical Research Information Technology Team will provide support for database programming for the trial and program the forms for data capture using REDCap. Carter Perry, MA will provide the statistical programming.

Maintenance of Trial Treatment Randomization Codes
The randomization scheme will be embedded in the data tracking system and access to the scheme will be restricted to the DMAC database programmer. In addition, once a school or participant classroom has been randomized, the group assignment will be locked. The randomization codes may only be broken at the request of the Data Safety Monitoring Board (DSMB) and the minimum number of DMAC staff necessary will be unmasked in order to provide requested analyses to the DSMB.

*Masking :* The Air Filter Classroom Intervention involves double-blinding, with placebo controlled randomization. The Sham Air Cleaner is designed to emit the same quiet background sound as the HEPA filter Air Cleaner. Staff from the HSPH Environmental Assessment and Intervention Core who do not have contact with the study participants will service and change the air filters every 3 months at the school after school hours, ensuring participants, PI, and study staff are blinded to classroom intervention assignment.

For IPM, the benefit of SICAS-2, compared to home-based interventions in which masking is impossible, is that it will be possible to single blind and mask the School IPM procedures. Students will not know whether or not IPM is being applied at the school---IPM procedures are done after hours when the students are not in school. Although it may be possible to attempt to mask some study staff from knowledge of IPM treatment by increasing the number of research assistants at each site (so that 1-2 RAs who ascertained clinical outcomes would not be involved in tasks related to IPM) this would substantially increase the budget. Thus double-blinding for IPM is not feasible or practical even in a school based study.

There are also several aspects of the study that will guard against bias that could result from having unmasked study staff. First, all laboratory assays will be conducted by laboratory technicians who will be masked to group assignments. Second, some of the clinical data that are collected, including exhaled nitric oxide and pre- and post- bronchodilator spirometry are objective measurements that are less subject to influence by the study staff or study participants. Third, secondary analyses will be conducted to determine if any improvements in clinical outcomes are associated specifically with decreases in school allergen, mold or other pollutant levels, not just with group assignment.

While randomization should result in minimal differences between treatment groups in home allergen levels, we will measure home allergens levels and these measures will enable us to control for any residual confounding by home allergen level in analyses. Home samples will be linked to participants as a surrogate measure for home exposure and for adjusted analyses. Home environmental assessments will also be done to differentiate the effect of home exposure in adjusted analyses with the primary focus of this study on the school/classroom specific exposure and clinical outcomes.

All laboratory studies (allergen ELISAs, allergen-specific IgE levels, endotoxin levels, PM 2.5, Black Carbon levels, trace elements, $NO_2$ levels, and mold) will be performed in centralized laboratories in batches to minimize variability of the assay.

## 13.4 Analysis Plan

### 13.4.1 Analysis Populations.

Analyses will be performed using an intent-to-treat (ITT) dataset which will include all participants who are randomized. Analyses will be performed using a per-protocol (PP) dataset and an intent-to-treat (ITT) dataset. The primary analysis will be an ITT analysis. After ensuring that any dropout is missing completely at random (see Missing Data Considerations below). The ITT dataset will include all participants who are randomized, and the per-protocol dataset will include only participants who have completed at least one intervention visit (one IPM/ Air Purifier visit) and one follow up clinical outcome visit.

### 13.4.2 Primary Analysis of Primary Endpoint(s)

**Multi-Level Modelling:**

We will employ multi-level models[111] to quantify the effects of interventions (both class-specific filters and school-wide IPM) on the clinical outcomes, while accounting for the multiple levels (school, classroom, student, time point) of data. Specifically, we will employ non-linear mixed effects models, which intrinsically handle unbalanced data (unequal number of classrooms within schools and unequal number of children with asthma within classrooms) while accounting for child, class, and school-level variability (clustering) in the outcome by treating these factors as random effects. SICAS-1 generally had no more than one to two SICAS asthma students per classroom, so that clustering by classroom will be minimal and classroom random effects are likely not necessary. Count data (such as our primary symptom outcome) will be analyzed by negative binomial regression and continuous outcomes by linear mixed models. Our primary analysis will contrast outcome levels in the intervention and control arms. For symptom-days, we will use the negative binomial regression:

(**Model 1):** $\log E[Y_{ijcs}] = b_0 + b_1 sch_s + b_2 cls_c + b_3 sch_s * post_j + b_4 cls_c * post_j + a^T z_{ij} + u_s + w_{ics}$

where $Y_{ijcs}$ is the number of symptom days for subject i in class c and school s on visit j, $sch_{sj}$ is the indicator of IPM treatment for school s, $cls_c$ is the class-specific filter treatment for class c at visit j, $post_j$ is an indicator variable for post- (versus pre-) randomization for a given visit, and $z_{ij}$ is a vector of potential confounders, including seasonal influences captured by visit number (1-5), and, in the unlikely event that home exposures are not balanced across treatment arms, home exposures.Visit number is the visit (0,1,2,3,4) on which a given measurement was recorded (reflecting time of year at which measurement was taken), as opposed to post which is 0/1 representing pre/post. In this model the main intervention effects ($sch_s$, $cls_c$) represent differences at baseline and allow us to check for imbalance in the randomization. Post is 0/1 for pre/post regardless of the timing of the post visit. We include visit number as a covariate into the analysis to account for the timing of each observation. The terms $u_s$ and $w_{ics}$ represent school-specific and subject-specific random effects, respectively, to account for longitudinal correlation among measurements taken on the same subjects ($w_{ics}$) and among measurements taken on different subjects within the same school ($u_s$). As is traditional in non-linear mixed effects models, we will assume each set of random effects are normally distributed with unknown variance estimated from the data. We will use standard diagnostic methods to check this normality assumption, with some of these having been developed by our group. In the likely event that there is balance in symptoms at baseline and the effects are absent, we can re-fit the model removing these main effects. The sch*post and cls*post terms are the terms of scientific interest and represent how post vs pre randomization changes in symptoms rates differ between intervention and control arms. Because we include visit number as a main effect we do not need to include the main effect of post-randomization. We note that this model is standard for longitudinal pre-post randomization designs[112]. Again, we will add class-specific random effects if subjects are clustered significantly within classrooms within a school. For continuous outcomes such as lung function, we will fit analogous linear mixed effect models**,** which takes the form

(**Model 2)**: $Y_{ijcs} = b_0 + b_1 sch_s + b_2 cls_c + b_3 sch_s * post_j + b_4 cls_c * post_j + a^T z_{ij} + u_s + w_{ics} + e_{ijcs}$,

where the covariates are defined as above for the negative binomial model and $e_{ijcs}$ are normally distributed errors**.** Extensions for secondary analyses (a)-(d) will be handled analogously to those for model (1) (**Please see secondary analyses section**). Standard diagnostics will be applied to detect non-normality of the errors, but experience suggests that this distributional assumption will be appropriate for lung function outcomes. Our primary analyses will entail fitting the models (1) and (2), with $Z_i$ terms including visit number (as a categorical variable) and home exposures that

correspond with the same exposure in school (ie. classroom/school mouse exposure adjusting for home mouse exposure). As written, these models will include the cls and sch intervention terms. Secondary analyses will include (a) the effect of intervention varies by time of year(visit/survey 1-5) (b) exposure concentrations as predictors,(c) whether the effect of intervention or exposure concentration is modified by sensitization or other asthma phenotype at baseline, and (d) assess whether there is a synergistic effect of the classroom and school-wide interventions. The terms $Z_{ij}$ will not include any post-baseline patient measurements that can be influenced by treatment assignment. The terms to be include in this vector will include visit number as a categorical variable, and home exposures. We will test $b_3$ and $b_4$ in separate hypothesis tests.

**Missing Data Considerations:**

**Missing Data:** Missed study visits and drop-outs will occur, and if these events occur completely at random or at random, then the use of multi-level models in the primary analyses will produce consistent estimates of the treatment effects and tests of these effects. It will be extremely rare, if it happens at all, that we will have symptom data for a subject just at baseline with no post-baseline measures. The only time this would happen is if a subject moves to a non-participating school for that academic school year. If this does occur we will first investigate whether dropout is associated with any covariates. If it is, then to obtain valid inferences for the intervention, we will have to control for those covariates explaining the missingness (see Fitzmaurice, Laird, Ware, Chapter 17-18). If it is completely at random (MCAR dropout), then it is acceptable to remove that subject from the analysis and only patients with some post-baseline symptom data will contribute to the primary analysis. As sensitivity analyses, we will conduct analyses using inverse probability weights, which effectively up-weights subjects that are more likely to dropout[113]. We may also have missing covariates, as opposed to dropout. The main tool for adjusting for missing covariate data will be multiple imputation methods, which can now be easily implemented in SAS (PROC MI and PROC MIANALYZE).

**Multiple Comparisons:** For symptoms, we have defined a single primary endpoint for analysis and no multiple comparisons adjustment will be made. For the mechanistic component, we will run the analysis separately for each endpoint and each DNA methylation mediator and will adjust for *multiple comparisons* using False Discovery Rates[114]

#### 13.4.3 Supportive Analyses of the Primary Outcome

The primary clinical outcome measure, maximum number of days of symptoms in the preceding two weeks, will be assessed in relation to classroom/school allergen/mold/particulate levels. The primary analysis will be conducted using the values of the primary outcome at the 2, 4, 6, and 8 month follow-up surveys. The 10 month survey can be used in tertiary analyses to assess for carry-over effects.

#### 13.4.4 Analyses of Secondary and Other Endpoint(s)/Outcome(s)

Secondary analyses will include (a) the effect of intervention varies by time of year (visit 1-5) (b) exposure concentrations as predictors, (c) whether the effect of intervention or exposure concentration is modified by sensitization or other asthma phenotype at baseline, (d) assess whether there is a synergistic effect of the classroom and school-wide interventions. _In (b), exposure concentrations will be matched to health outcomes according to the season in which a particular visit falls. Our preliminary data from SICAS-1 shows that any potential error induced by aligning

health and exposure data in this way will not materially diminish our ability to detect intervention effects, as SICAS-1 analyses showed significant effects using this same design/plan. Also, our preliminary intervention study demonstrated exposure reductions of ≥ 75%. This is greater than any seasonal variability demonstrated in classroom exposures in SICAS-1[9,22], and will allow us to detect the effects of our intervention, regardless. For (c), we will add sensitization, sensitization*visit number, sensitization*$cls_c$, and sensitization*$sch_s$ terms to the model. For (d), to models (1) and (2) we will add $cls_c * sch_s$ and $cls_c * sch_s * post_j$ interaction terms.

Other factors that may modify the effect of the intervention on outcomes include level of mouse allergen exposure, number of other allergic sensitivities, ( i . e . the combination of cat exposure+sensitization, and/or the combination of cockroach exposure+sensitization). Additional clinical characteristics that might affect responsiveness to the intervention include severity of asthma, lung function, and FeNO concentrations. Analyses will again include interaction terms in models to determine of any of these factors predict responsiveness to the intervention.

In secondary analyses, we will adjust for factors that may affect responses to treatment such as the degree of exposure at baseline, the degree of sensitivity to mouse, number of other allergies, and asthma severity. Because hospitalizations, ED visits, unscheduled visits, and corticosteroid bursts are relatively infrequent events, we will assess the distribution of these variables and may decide to dichotomize the outcome as any vs. none and use logistic models.

Other secondary clinical outcome variables that will be examined will include those listed above in **Table 1.**

Other potential confounders will be explored by examining differences between the intervention and control groups and also assessing the association between the potential confounders at screening and at each study visit. In addition, potential confounders will be included in the models to determine the impact the variables have on the relationship between group assignment and the outcome. Some specific confounders that will be carefully examined include, cockroach sensitization and exposure (SICAS-1 had minimal to no cockroach exposure in schools and homes[9]), medication use, upper and lower respiratory infections, changes in exposure to other pollutants and other allergens (note that cat and dogs as pets in schools are not allowed but exposure levels from home and school will be available), environmental tobacco smoke exposure (smoking in schools is banned), home exposures, and endotoxin levels, age, gender, race, household income, perceived stress[29], body mass index[115], and season. Effect modifiers, including the degree of allergen/pollutant reduction, will also be examined by creating an interaction term to include in the final models.

**Mechanistic Outcome Analysis:**

**Mechanistic Analytical Plan:**

**Pathway analysis**: Analysis of the data from the mechanistic portion of the study will focus on pathway-specific effects of the intervention on genome-wide methylation and expression, and their downstream effects on asthma symptoms. Because we will have genome-wide methylation and mRNA expression data collected before and after the intervention, analyses will apply to post-pre differences in site-specific methylation and expression, and assess whether these differences relate to intervention group and asthma symptom. Within this design, in order to uncover pathways or

methylation/expression signatures associated with the intervention and asthma symptom reduction, we will perform (1) a **semi-supervised** pathway analysis and (2) an **unsupervised** methylation/expression signature analysis. These analyses will use all available genes on the chip but can also be used to examine association with candidate pathways. DNA methylation and mRNA expression will be analyzed separately and their results will be cross referenced to interpret the function of genes identified.

Pathway perturbed by intervention and affects asthma symptom and related outcomes

Separately for methylation and expression results, we will first cross reference association results with intervention and asthma symptom to identify pathways and gene networks that are perturbed by intervention and subsequently affect asthma symptoms and related outcomes. In addition to estimating the association of methylation and expression with each intervention and with asthma symptoms, we will also estimate how much the intervention effect on asthma symptoms is through methylation/expression changes using **causal mediation analysis**. Based on the theory of counterfactuals (e.g. what a subject's methylation/expression and symptoms would have been if that person had been instead randomized to the opposite treatment arm), we will apply the VanderWeele and Vansteelandt[82] approach, which is very general in that it allows for the possibility of intervention-mediator (methylation/expression in this case) interactions on symptom outcomes, to estimate the direct and indirect (through a methylation/expression mechanism) effect of intervention on asthma symptoms (or other clinical endpoint). These models will include both interventions (classroom air filter and school-wide IPM). In the absence of intervention and methylation/expression interaction, this approach reduces to the well-known approach of Baron and Kenney[79]. This class of models, which can be easily extended to count outcomes via Poisson or negative binomial regression and also to include random effects to account for the longitudinal design of the study, is one with which our investigative team has extensive prior experience[99,116] **.**

To interpret downstream functional role of the identified methylation loci, we will cross reference pathways and networks identified from methylation data and expression data and test for association between methylation changes and expression changes of the corresponding genes.

Effect modification due to sensitization and related phenotypes

We will repeat the above association analyses by including interaction terms with sensitization and related phenotypes. In model assessing the association of intervention with methylation and gene expression, the interaction term in the model will be each intervention indicator by sensitization or related phenotypes. For association of asthma symptom and methylation and expression, the interaction term in the model will be methylation/expression and sensitization and related phenotypes. We will also characterize subjects based on their methylation/expression patterns at baseline before intervention. We expect that part of these patterns might reflect epithelial and downstream mast cell, $T_H2$ or other functions measurable in nasal samples. We will examine whether molecular characteristics before intervention modifies the size of effect and influences which gene pathways are changed by intervention and lead to downstream reduction of asthma symptoms.

Correction of potential confounding due to cell counts and batch effects

Although we will completely randomized the samples in methylation/expression batches (run, plate, chip, etc) so that batches would not be correlated with intervention and asthma symptoms, thus not likely to be confounding factors. We will examine the sensitivity of our result to potential confounding due to cell counts and batch effects. We will use the experimentally obtained cell counts and the batch effects estimated by surrogate variable analysis (SVA)[117] as covariates in the association analysis. Before computing the post-pre difference in methylation/expression, we will regress out the cell count effect and batch effect first and then calculate the difference in residual methylation/expression.

We will repeat the above association analyses by including interaction terms with sensitization and related phenotypes. In the association of intervention with methylation and gene expression, the interaction term in the model will be intervention indicator and sensitization and related phenotypes. For association of asthma symptom and methylation and expression, the interaction term in the model will be methylation/expression and sensitization and related phenotypes. We will also characterize subjects based on their methylation/expression patterns at baseline before intervention. We expect that part of these patterns might reflect epithelial and downstream mast cell, $T_H2$ or other functions measurable in nasal samples. We will examine whether molecular characteristic before intervention modifies the size of effect and influences which gene pathways will be changed by intervention and lead to downstream reduction of asthma symptom.

Selection of CpGs and genes for technical validation using pyrosequencing and realtime PCR

We will select representative genes and CpGs in expression/methylation pathway and network. The representative genes/CpGs are defined as those that show the most significant individual association with intervention and asthma symptom. We will also select the hub genes/CpGs that show the strongest connection with other genes/CpGs in the same pathway and network.

**Relation of mechanistic study design to clinical trial**

Analysis of the data from the mechanistic portion of the study will focus on pathway-specific effects of the intervention on genome-wide methylation and expression, and on the downstream effects of changes in these intermediate outcomes on asthma symptoms and other relevant clinical outcomes. In evaluating whether school environment exposure abatement results in molecular changes that modify the course of asthma, we will not only expand our understanding of asthma immunopathogenesis, but will also create opportunities to identify potential novel targets for asthma phenotype-specific therapy.

### 13.4.5 Analyses of Exploratory Endpoint(s)

Exploratory data analysis will include compilation of descriptive statistics to detect any outliers or discrepancies in data and to compare baseline characteristics and demographics between IPM and Control, as well as between Filter and Control, Groups. Continuous variables will be summarized using means, medians, standard deviations, ranges, and interquartile ranges. Continuous variables will be analyzed using non-parametric approaches or will be transformed to meet assumptions of normality required for parametric statistics. Categorical variables will be tabulated. The percent of participants who complete the study, losses to follow-up, missed visits, and reasons for discontinuation will be presented.

### 13.4.6 Descriptive Analyses

**Descriptive Analyses**

The change in settled dust allergen levels will be compared between the 4 Groups to assess efficacy of allergen reduction. Asthma symptoms, rescue medication use, and health care utilization will be compared between IPM/Air Filter and Control Groups to assess efficacy of the intervention in reducing asthma symptoms and morbidity.

Descriptive statistics to detect any outliers or discrepancies in data and to compare baseline characteristics and demographics between IPM and Education Groups. Continuous variables will be summarized using means, medians, standard deviations, ranges, and interquartile ranges. Continuous variables will be analyzed using non-parametric approaches or will be transformed to meet assumptions of normality required for parametric statistics. Categorical variables will be tabulated. The percent of participants who complete the study, losses to follow-up, missed visits, and reasons for discontinuation will be presented.

### 13.5 Interim Analyses (Not Applicable: Environmental Intervention- NOT a medication, high risk intervention)

### 13.6 Statistical Hypotheses

**Clinical Trial Hypothesis and Aims:** Our central hypothesis is that reducing classroom/school exposure to particles and their associated allergens (particularly mouse/mold) will decrease asthma morbidity in students with asthma. In **S**chool **I**nner-**C**ity **A**sthma Intervention **S**tudy-**2** (**SICAS-2**) we plan to test our central hypothesis in an intervention study of 300 elementary school-aged children with asthma from multiple classrooms in 40 inner-city elementary schools.

**Aim 1:** To determine the effectiveness of a school/classroom based environmental intervention [**Classroom** particle air filter/purifying units and school-wide integrated pest management (IPM)] to:

(a) Reduce asthma symptoms (primary outcome) as used in other NIAID inner-city asthma studies[1,3];

(b) Reduce medication use, health care utilization, and improve lung function (secondary outcomes).

**Mechanistic Aims:**

**Aim 2:** To test the hypothesis that effects of school/classroom-based environmental interventions on symptoms/other measures of asthma control occur through changes in gene methylation or expression in pathways (and secondarily, in genes) relevant to airway function and asthma. Within pathways or networks of genes (pre-specified or discovered in unsupervised analyses) we will (a) determine how our interventions influence changes in nasal airway cell methylation or gene expression and (b) evaluate how our intervention-associated changes in methylation and gene expression influence asthma outcomes. Finally, using mediation analysis, we will (c) estimate how much of the intervention effects on asthma symptoms occurs through methylation/expression changes[118,119].

**Secondary Aim 3:** To explore whether sensitization or other asthma phenotypes defined at baseline modify which pathways or networks of genes undergo intervention-related changes in methylation or gene expression that, in turn, are associated with improvement in asthma outcomes.

### 13.7 Sample Size Considerations Sample Size Estimates:

**Power and Sample Size Calculations: (a) Primary Clinical Outcome:** We include the Annual Study Schema Figure 1) to aid in understanding both the statistical analysis plan and the power calculations. For the primary clinical outcome, assumptions for the sample size estimate for the symptom outcome are based on classroom/exposure and symptom data from SICAS-1. The proposed primary statistical analysis assessing the impact of the interventions (school- and classroom-based) is based on a multilevel negative binomial regression model, which simultaneously accounts for correlation among clustered observations while accounting for over-dispersion in the symptom counts We calculated power using a simulation-based approach that repeatedly generated data under this model, and calculating the proportion of time we reject the null hypothesis of no school-wide IPM effect (H0: $b_3$=0) and no classroom filter effect (H0: $b_4$=0). We assumed $\theta = 3$. The intercept $b_0$ = log(3) was chosen to correspond to a rate of three symptom days at baseline prior to the intervention. The amount of overdispersion in the negative binomial distribution was set equal to that estimated in SICAS-1. Specifically, this set was using the overdispersion parameter "theta" in the rnegbin function in the MASS package in R. The random effects standard deviations were set equal to 0.5 and 0.2 for the subject and school random effects, respectively. All power calculations were run assuming a two-sided alpha=0.05 level test, and all power estimates are based on 1,000 simulated datasets. Power calc table 2 shows that with a total n = 240, we have >80% power to detect a difference of at least 0.6 days of symptoms and >90% power to detect a difference of 0.75 days between the two groups. Taking into account a conservative projected 20% dropout rate (SICAS-1 > 90% retention), we will enroll 300 participants to ensure that 240 will complete the study. To check our power to detect associations among quintiles of the exposure distribution (as a continuous variable, ), we used the same simulation strategy based on a multilevel negative binomial regression model but with exposure quintile as a covariate.

Using this approach, we estimate that we will have 90% power to detect the slope estimated in SICAS-1 of 0.173 (the effect size in Figure 2), which represents the log relative rate per increase in exposure quintile, in as little of 40 subjects. This result demonstrates the increase in power that results in using the continuous exposure concentrations, when such an effect in fact exists, and suggests that we will be able to detect such associations even if this effect exists only in subjects sensitized to mouse and mold (our SICAS-1 data supports that we will have more than 40 sensitized subjects).

Our power calculations for our primary outcome were determined by the SICAS-1 data on exposure, sensitization and symptoms for mouse and mold, and the pollutant exposures for the entire population and supports that the SICAS-2 population will have adequate power to test our primary hypotheses.

We also estimated our power to detect intervention effects on secondary outcomes, specifically $FEV_1$ reflecting pulmonary function. We used the same simulation approach outlined above for symptoms, but simulating from a linear

mixed effects model for a continuous outcome instead of a negative binomial regression model for counts. We used as variance component estimates values taken from unpublished analyses of the association between $FEV_1$ and air pollution exposure our group (Gold, Coull, Koutrakis) has just recently completed on data from the Childhood Asthma Management Program (CAMP) study. Specifically, from our CAMP analyses using outcome measures taken quarterly, we assumed values of 10 and 6 for the subject-specific and residual error terms, yielding a subject-specific intraclass correlation of approximately 0.6. We assumed a value of 6 for the school-specific error term, yielding a school-specific intraclass correlation of 0.2. For ease of interpretability, following the convention of Cohen[120]we express the minimal detectable effect size as a percentage of the residual standard deviation of the outcome, with 0.2 representing a "small" effect size, 0.5 representing a medium effect size, and 0.8 representing a large effect size. Under these assumptions, we estimated that we will have 80% (90% in parentheses) power to detect an Cohen's effect size (for either class or school-wide intervention) of 0.3 (0.4), suggesting we will have the power to detect relatively modest effects of intervention based on n=240 after a conservative estimate of 20% dropout (again, we observed <10% dropout in SICAS-1). Existing published results suggest the effect of particle exposures may be at least moderate, with an reported correlation between PM2.5 and FEV1 of -0.72 (p<0.01) in Southern California public schoolchildren from the Children's Health Study[121]. Therefore we are likely to have sufficient power to detect plausible effect sizes of the intervention.

| | Sample Size Estimates: **Table 2** | | |
|---|---|---|---|
| Δ sx days/2 weeks | Total N | Power: IPM School | Power: Class |
| 0.60 | 240 | 84 | 89 |
| | 280 | 91 | 92 |
| 0.75 | 240 | 96 | 98 |
| | 280 | 99 | 99 |
| 0.90 | 240 | 99 | 100 |
| | 280 | 100 | 100 |

Analysis will compare IPM versus Control Schools and Air Filter Versus Sham Classrooms, and efficiently testing the effectiveness of the intervention in real life, translatable practicality to all schools, since likely IPM affects school wide exposures, while air filters will affect classroom specific exposures. The power calculations are based on a two-sided 0.05 alpha level test

**Economic Evaluation**
**Introduction: Clinical Significance/Relevance of Primary Outcome/ Effect Size:**

With our sample size, including accounting for drop out, we have 95-98% power to detect a school IPM/Classroom treatment effect of a decrease of at least 0.75 days of symptoms per two week period, and 82-89% power to detect a treatment effect of 0.6 days decrease. In comparison, the ICAS home-based environmental intervention detected a 0.8 day difference in symptom days/2 weeks, and 2.85 children needed to be treated to prevent one unscheduled visit/year[1,15] The NIAID-funded ICAS home-based study was not powered to focus on mouse allergen intervention, but demonstrated that only a 50% reduction in mouse allergen levels in home environments provide clinically significant results[15]. We have demonstrated in a school setting the ability to reduce allergen levels by at least that level.

Perhaps more importantly, as highlighted by the ICAS investigators, this effect size is similar to that seen with inhaled corticosteroids in the Childhood Asthma Management Program[122] Inhaled corticosteroids are recommended in asthma

because they have a clinically meaningful impact on disease outcomes, even at a cost of approximately $1,500/year. In a Boston home-based Community Asthma Initiative of education and referral for home IPM, this simple intervention provided a health care savings of $2,250 per child and a return on investment of 1.46 (break even 1.0)[74]. An environmental intervention with a similar effect size should have a clinically meaningful impact on asthma and be cost-effective by promoting policy changes in school environments and targeting many children in a community at once.

**Overview of Economic Evaluation** This intervention has the potential to generate long-lasting and broad economic benefits to school children with asthma, their caregivers and society overall. As for the ICAS home intervention,[1] our school intervention is most immediately relevant to urban U.S. children in the many schools where asthma morbidity is high and school exposure levels are similar to those that we have measured. In 2008, an estimated 136,267 children in Massachusetts had asthma [123]. Active asthma rates are markedly higher and asthma morbidity is greater in urban areas of Massachusetts[117,124,125]. Of 7032 school children screened in the 40 schools from which SCIAS-1 participants were drawn, 22% had a history of asthma, which for most children was active and involved health care utilization. Of the 7032, 15% had asthma-related missed school days or medical care use in the past 12 months, and 17% had night time asthma symptoms. We estimate each of our interventions will provide a decrease of ≥0.75 days of symptoms/2 weeks or ≥19.5 symptom free days (SFDs)/ year per person. In the Inner City Asthma home intervention trial, 4.82 symptom days were associated with 1 lost school day[1]. The magnitude of the problem—the high urban prevalence of asthma-related missed school days or medical care use in school children--along the estimated benefits of our intervention suggests large societal benefits from our trial. In contrast to home-based interventions, we expect 'economies of scale' as similar in magnitude investments now benefit entire classrooms; (i) 'spill-over' benefits to families and the economy stemming from fewer missed work days; (ii) fewer missed school days which correlates with school performance and likely has cumulative societal benefits, such as lower dependence on welfare programs, reduced likelihood of special education assignment and greater lifetime earnings[126]; as well as (iii) reductions in health cost from decreased ED, inpatient and outpatient care. Because that our intervention could lead to reduction in SFDs and consequent economic as well as social benefits as high as 15% of the urban school population, we hypothesize that the interventions will lead to net savings and improved quality of life, and will be cost-effective.

**Cost-Effectiveness Analysis (CEA) Plan:** Adopting the societal perspective, we will estimate the net costs/savings of each intervention and will conduct a formal cost-effectiveness analysis (CEA). We will follow closely the Asthma Outcomes workshop recommendations for measurement of asthma-specific health care utilization and costs for NIH-initiated research[127]

(1) We first estimate the costs of annualized capital equipment (total purifier costs divided by years of useful life); equipment, materials for IPM evaluation and extermination, pest control education and targeted cleaning; and wages for technicians conducting particle reductions; IPM education and extermination( using wages for IPM professionals in real world settings). The self-reported time that it takes for education and IPM targeted cleaning will be multiplied by the US Bureau of Labor Statistics average wages (by area and occupation).;

(2) We will then calculate health care costs and potential savings that might result from lower rates of outpatient, ED and inpatient medical care (including scheduled and unscheduled visits and school nurse visits) and reduced use of rescue medications. Utilization data will be collected through surveys every two months (using a 2-week recall period) and monetized using state-specific average costs by setting. The number of days that parents and children missed from

work and school, respectively, will be recorded at each data collection wave. For parents we will use the human capital approach (lost wages); school absence will be valued using caregiver's daily wages.

(3) We will use two measures of effectiveness,(1) symptom-free days (SFDs) (using self-reports), and (2)gains in quality-adjusted life years (QALYs), a well-demonstrated internationally accepted tool for estimating burden of disease and benefits accruing from disease reduction. For the latter, we will use the commonly used instrument EQ-5D-Y (Youth) to measure health-related quality of life (http://www.euroqol.org/). Studies have shown that SFDs strongly correlates with QALYs [128]In a recent study, $7.50 / SFD translated into $13,500 / QALY). QALYs have been recommended for CEA of asthma interventions[127].

**Cost-Effectiveness Statistical analysis.** We will calculate total inflation-adjusted costs over 12-months on the aggregate, per school, classroom, and per person as well as QALYs and number of symptom days per person. Incremental costs and effects across arms will be estimated using generalized linear models (e.g., with a long link for costs), adjusting for relevant confounders and for clustering as described elsewhere. In the event of a large proportion of individuals with zero health expenditures, we will consider the use of two part models for health care costs (e.g. a logit model to predict any utilization, followed by a generalized linear model of expenditures among the subgroup with non-zero utilization) . Next, we will estimate the incremental cost-effectiveness ratio (ICER) between trial arms, including cost per SFD and per QALY.
ICER= $Cost_{Intervention\ group}$ - $Cost_{Control\ group}$/$SFD_{Intervention\ group}$ - $SFD_{Control\ group}$=Incremental cost/Incremental effect.
Probabilistic sensitivity analyses (based on bootstrapping with replacement) will be used to construct 95% CIs around the ICERs, and/or cost-effectiveness acceptability curves (CEACs).

**Interpretation:** We hypothesize that the interventions will be effective and will also reduce total societal costs. If the intervention costs are more expensive than savings in health care use (including medications), we will calcula;te the probability of cost effectiveness at different willingness to pay thresholds (this information is presented in the CEACs). We will be guided by widely accepted (50,000/QALY) and more recent estimates [129]of the threshold societal willingness to pay for an additional QALY to determine when interventions are expected to be cost-effective and are therefore, worth implementing.

## 14. Identification and Access to Source Data

### 14.1. Source Data

Source documents and source data are considered to be the original documentation where subject information, visits consultations, examinations and other information are recorded. Documentation of source data is necessary for the reconstruction, evaluation and validation of clinical findings, observations and other activities during a clinical trial. The Data Coordinating Center (DCC) lead will manage the data repository for the clinical and safety database. She will generate reports for review by the Protocol Chair on at least a monthly basis or more frequently as dictated by the rate of participant enrollment and availability of results.

The investigator or designee will to the best of his/her ability encourage compliance in completing case report forms for every participant entered into the trial without being coercive. In addition, the investigator or designee is responsible for the reliability of the data collected and recorded in the database and will review and sign the pertinent case report forms, namely adverse events, severe adverse events, and protocol deviations when completed. Lung function tests will be reviewed and signed by PI or qualified designee (i.e. certified pulmonology specialist, asthma/allergy specialist).

All study questionnaires and CRFs may be directly entered into the RECAP Database. Data collected on hard copy forms will be entered into a REDCAP database, which is a web- based data entry system located on the BCH network. It provides audit trails for tracking data changes and user activity as well as automated export procedures for seamless downloads to Excel and common statistical packages. The database has range and valid value checks to ensure integrity at the time of data entry. Out of range values must be confirmed by the staff person conducting data entry at the time of entry. Missing values will be flagged at the time of data entry and reported to the project manager for follow-up and resolution. Hard copy source documents will be used to verify missing data or discrepancies whenever available.

The study staff will complete data entry into REDCap as data are collected and at the latest by the last day of the month during which data was collected so data the manager may generate accurate and timely reports. Data will also be reviewed on an ongoing basis and in real time by the by the Data Manager. If the Data Manager finds discrepancies or missing data, she will assign data queries to the project manager. It will be the data manager's responsibility to check the data field against the source documentation. S/he will then respond to the query either by verifying the entered value or correcting the value. If the Data Manager finds the response acceptable, she will make the appropriate correction to the database and close the query. Data queries will be therefore be resolved promptly after identification at the time of data entry as well as at monthly intervals during data monitoring and cleaning activities throughout the year.

Several procedures will be in place to confirm the completeness, consistency, and accuracy of all documented data within the database(s) for this trial. These include computerized edit checks at the time of data entry and data query resolution procedures as described above. In addition, double data entry will be performed on a randomly selected sample of study forms, with the percentage depending on relevance to primary and secondary outcomes. Double data entry will be conducted by a second study staff member to evaluate the degree of keying errors and on an ongoing basis during data collection. The data manager will run monthly reports comparing the double entry for discrepancies. If any errors are found, a query will be opened and directed to the project manager, who will need to return to the source documentation to resolve the issue. The project manager will reply with the correct data that should be captured, make the appropriate change, and save the form. The data manager will review changes and close query. Finally, various study status and quality reports will also be programmed and generated by the DCC to monitor study progress, data integrity, and safety. These will include reports that monitor form expectation and completion rates, visit window compliance, missing data and out of range values, recruitment and enrollment rates, and withdrawal or loss to follow-up rates. Adverse event reports and Protocol Deviation Reports will also be generated routinely by the DMAC. Adverse events, protocol deviations study status and data quality reports will be reviewed on an ongoing basis by the PI. In addition, these reports will be generated periodically for DSMB review.

### 14.2. Access to Source Data

The site investigators and site staff will make all source data available to the DAIT/NIAID, as well as to relevant health authorities. Authorized representatives as noted above are bound to maintain the strict confidentiality of medical and research information that may be linked to identified individuals.

## 15. Protocol Deviations

### 15.1. Protocol Deviation Definitions

**Protocol Deviation** – The investigators and site staff will conduct the study in accordance to the protocol; no deviations from the protocol are permitted. Any change, divergence, or departure from the study design or procedures constitutes a protocol deviation. As a result of any deviation, corrective actions will be developed by the site and implemented promptly.

**Major Protocol Deviation (Protocol Violation) -** A Protocol Violation is a deviation from the IRB approved protocol that may affect the subject's rights, safety, or well-being and/or the completeness, accuracy and reliability of the study data. In addition, protocol violations include willful or knowing breaches of human subject protection regulations, or policies, any action that is inconsistent with the NIH Human Research Protection Program's research, medical, and ethical principles, and a serious or continuing noncompliance with federal, state, local or institutional human subject protection regulations, policies, or procedures.

**Non-Major Protocol Deviation** - A non-major protocol deviation is any change, divergence, or departure from the study design or procedures of a research protocol that does not have a major impact on the subject's rights, safety or well-being, or the completeness, accuracy and reliability of the study data.

### 15.2. Reporting and Managing Protocol Deviations

The study site principal investigator has the responsibility to identify, document and report protocol deviations as directed by the study Sponsor (DAIT/NIAID) . However, protocol deviations may also be identified during site monitoring visits or during other forms of study conduct review.

Upon determination that a protocol deviation has occurred, the study staff will a) notify the site Principal Investigator, b) notify the NIAID Project Manager, the DMAC, and c) will complete a Protocol Deviation form. The DAIT/NIAID Project Manager will make the decision as to whether the Deviation is major or not and what the impact of the Deviation on the study participant or the entire study may be. PI will submit the Protocol Deviation reports to the appropriate review bodies (IRB, DAIT/NIAID.) and who will review and approve the action plan that will be implemented as a result of the Protocol Deviation.

## 16. Ethical Considerations and Compliance with Good Clinical Practice

### 16.1. Statement of Compliance

This clinical study will be conducted using good clinical practice (GCP), as delineated in Guidance for Industry: E6 Good Clinical Practice Consolidated Guidance, and according to the criteria specified in this study protocol. Before study initiation, the protocol and the informed consent documents will be reviewed and approved by the IRB. Any amendments to the protocol or to the consent materials will also be approved by the IRB before they are implemented. The Sponsor (DAIT/NIAID) will review and approve the Protocol and consent documents, and all changes in addition to IRB.

### 16.2. Informed Consent Process

The consent process will provide information about the study to a prospective participant and will allow adequate time for review and discussion prior to his/her decision. The principal investigator or designee listed on the IRB approved

Protocol will review the consent and answer questions. The designees will all be trained in human subjects' protection and conflict of interest and able to provide informed consent per Institution guidelines. The prospective participant will be told that being in the trial is voluntary and that he or she may withdraw from the study at any time, for any reason. All participants (or their legally acceptable representative) will read, sign, and date a consent form before undergoing any study procedures. Consent materials will be presented in participants' primary language. Spanish-speaking participants will be provided with consent documents in Spanish and the consent discussion will be facilitated or conducted by a translator. A copy of the signed consent form will be given to the participant.

The consent process will be ongoing. The consent form will be revised when important new safety information is available, the protocol is amended, and/or new information becomes available that may affect participation in the study.

### 16.3. Privacy and Confidentiality

A participant's privacy and confidentiality will be respected throughout the study. Each participant will be assigned a unique identification number and these numbers rather than names will be used to collect, store, and report participant information. Site personnel will not transmit documents containing personal health identifiers (PHI) to the study sponsor (DAIT/NIAID) or their representatives.

## 17. Quality Control and Quality Assurance

The investigator is required to keep accurate records to ensure that the conduct of the study is fully documented. The investigator is required to ensure that all CRFs are completed for every participant entered in the trial. Quality Control and Quality Assurance processes for data handling are discussed in Section 12.1 above.

The sponsor is responsible for regular inspection of the conduct of the trial, for verifying adherence to the protocol, and for confirming the completeness, consistency, and accuracy of all documented data.

In accordance with applicable regulations, GCP, and procedures, PPD will contact the site prior to the start of the study to review with the site staff the protocol, study requirements, and their responsibilities to satisfy regulatory, ethical, and protocol requirements. When reviewing data collection procedures, the discussion will include identification, agreement and documentation of data items for which the CRF will serve as the source document.

PPD contracted by DAIT/NIAID will monitor the study to ensure that the:

- Data are authentic, accurate, and complete.
- Safety and rights of subjects are being protected.
- Study is conducted in accordance with the currently approved protocol and any other study agreements, GCP, and all applicable regulatory requirements.

The investigator agrees to allow the monitor direct access to all relevant documents.

To ensure compliance with GCP and all applicable regulatory requirements, PPD may conduct a quality assurance assessment and/or audit of the site records, and the regulatory agencies may conduct a regulatory inspection at any time during or after completion of the study. In the event of an assessment, audit or inspection, the investigator (and

institution) must agree to grant the advisor(s), auditor(s) and inspector(s) direct access to all relevant documents and to allocate their time and the time of their staff to discuss the conduct of the study, any findings/relevant issues and to implement any corrective and/or preventative actions to address any findings/issues identified.

17.1 Data Handling

The investigator is required to ensure that all CRFs are completed for every participant entered in the trial. All elements of data entry (i.e., time, date, verbatim text, and the name of the person performing the data entry) will be recorded with an audit trail to allow all data changes in the database to be monitored and maintained in accordance with federal regulations.

## 18. Publication Policy

Every possible effort will be made for the primary outcome of the trial to be published in a peer-reviewed journal within 12 months after the database is locked, that DAIT/NIAID will review and comment on any manuscript derived from this trial prior to submission and that, if the trial is funded by a NIAID grant of > $500,000 direct costs/year, the NIH data sharing policy applies (http://grants.nih.gov/grants/policy/data_sharing/data_sharing_guidance.htm#goals) and that data from this trial will be shared in accordance to the specific plan that was included in the grant application.

## 19. References

1. Morgan WJ, Crain EF, Gruchalla RS, et al. Results of a home-based environmental intervention among urban children with asthma. N Engl J Med 2004;351:1068-80.
2. Mitchell H, Senturia Y, Gergen P, et al. Design and methods of the National Cooperative Inner-City Asthma Study. Pediatric pulmonology 1997;24:237-52.
3. Rosenstreich DL, Eggleston P, Kattan M, et al. The role of cockroach allergy and exposure to cockroach allergen in causing morbidity among inner-city children with asthma. N Engl J Med 1997;336:1356-63.
4. Phipatanakul W, Bailey A, Hoffman EB, et al. The school inner-city asthma study: design, methods, and lessons learned. J Asthma 2011;48:1007-14.
5. Wildfire JJ, Gergen PJ, Sorkness CA, et al. Development and validation of the Composite Asthma Severity Index--an outcome measure for use in children and adolescents. The Journal of allergy and clinical immunology 2012;129:694-701.
6. Krishnan JA, Lemanske RF, Jr., Canino GJ, et al. Asthma outcomes: symptoms. The Journal of allergy and clinical immunology 2012;129:S124-35.
7. Vital signs: asthma prevalence, disease characteristics, and self-management education: United States, 2001--2009. MMWR Morbidity and mortality weekly report 2011;60:547-52.
8. Joseph CL, Foxman B, Leickly FE, Peterson E, Ownby D. Prevalence of possible undiagnosed asthma and associated morbidity among urban schoolchildren. J Pediatr 1996;129:735-42.
9. Permaul P, Hoffman E, Fu C, et al. Allergens in urban schools and homes of children with asthma. Pediatric allergy and immunology : official publication of the European Society of Pediatric Allergy and Immunology 2012;23:543-9.

10. Sheehan WJ, Rangsithienchai PA, Muilenberg ML, et al. Mouse allergens in urban elementary schools and homes of children with asthma. Annals of allergy, asthma & immunology : official publication of the American College of Allergy, Asthma, & Immunology 2009;102:125-30.
11. Permaul P, Petty C, Sheehan W, et al. Mouse Allergen Exposure in Urban Schools and its Effect on Childhood Asthma Morbidity. J Allergy and Clin Immunol 2013;131:AB 141.
12. Baxi S, Petty C, Fu C, et al. Classroom Fungal Spore Exposure and Asthma Morbidity in Inner-City School Children. J Aller Clin Immunol 2013;131:AB 54.
13. Gaffin J, Petty C, Sheehan WJ, et al. Classroom-Based Air Pollution Effects on Asthma Morbidity in Inner City School Children. Amer J Respir Crit Care Med 2013;187:A2521.
14. Matsui EC, Eggleston PA, Buckley TJ, et al. Household mouse allergen exposure and asthma morbidity in inner-city preschool children. Annals of allergy, asthma & immunology : official publication of the American College of Allergy, Asthma, & Immunology 2006;97:514-20.
15. Pongracic JA, Visness CM, Gruchalla RS, Evans R, 3rd, Mitchell HE. Effect of mouse allergen and rodent environmental intervention on asthma in inner-city children. Annals of allergy, asthma & immunology : official publication of the American College of Allergy, Asthma, & Immunology 2008;101:35-41.
16. Butz AM, Matsui EC, Breysse P, et al. A randomized trial of air cleaners and a health coach to improve indoor air quality for inner-city children with asthma and secondhand smoke exposure. Arch Pediatr Adolesc Med 2011;165:741-8.
17. Lanphear BP, Hornung RW, Khoury J, Yolton K, Lierl M, Kalkbrenner A. Effects of HEPA air cleaners on unscheduled asthma visits and asthma symptoms for children exposed to secondhand tobacco smoke. Pediatrics 2011;127:93-101.
18. Phipatanakul W, Cronin B, Wood RA, et al. Effect of environmental intervention on mouse allergen levels in homes of inner-city Boston children with asthma. Annals of allergy, asthma & immunology : official publication of the American College of Allergy, Asthma, & Immunology 2004;92:420-5.
19. Gaffin JM, Raby BA, Petty CR, et al. beta-2 Adrenergic receptor gene methylation is associated with decreased asthma severity in inner-city Schoolchildren: Asthma and Rhinitis. Clin Exp Allergy 2014;44:681-9.
20. Permaul P, Sheehan WJ, Baxi SN, et al. Predictors of indoor exposure to mouse allergen in inner-city elementary schools. Annals of allergy, asthma & immunology : official publication of the American College of Allergy, Asthma, & Immunology 2013;111:299-301 e1.
21. Baxi SN, Muilenberg ML, Rogers CA, et al. Exposures to molds in school classrooms of children with asthma. Pediatric allergy and immunology : official publication of the European Society of Pediatric Allergy and Immunology 2013;24:697-703.
22. Sheehan WJ, Hoffman EB, Fu C, et al. Endotoxin exposure in inner-city schools and homes of children with asthma. Annals of allergy, asthma & immunology : official publication of the American College of Allergy, Asthma, & Immunology 2012;108:418-22.
23. Kanchongkittiphon W, Sheehan WJ, Friedlander J, et al. Allergens on desktop surfaces in preschools and elementary schools of urban children with asthma. Allergy 2014;Epub ahead of print.
24. Baxi SN, Sheehan WJ, Gaffin JM, et al. Agreement between parent and student responses to an asthma and allergy questionnaire in a diverse, inner-city elementary school population. Annals of allergy, asthma & immunology : official publication of the American College of Allergy, Asthma, & Immunology 2011;107:371-3.
25. Arbes SJ, Jr., Sever M, Vaughn B, et al. Feasibility of using subject-collected dust samples in epidemiologic and clinical studies of indoor allergens. Environmental health perspectives 2005;113:665-9.
26. Curtin-Brosnan J, Paigen B, Hagberg KA, et al. Occupational mouse allergen exposure among non-mouse handlers. J Occup Environ Hyg 2010;7:726-34.
27. Platts-Mills TA. Allergen avoidance in the treatment of asthma: problems with the meta-analyses. The Journal of allergy and clinical immunology 2008;122:694-6.

28. Ogden CL, Carroll MD, Curtin LR, Lamb MM, Flegal KM. Prevalence of high body mass index in US children and adolescents, 2007-2008. Jama 2010;303:242-9.
29. Islam T, Urman R, Gauderman WJ, et al. Parental stress increases the detrimental effect of traffic exposure on children's lung function. American journal of respiratory and critical care medicine 2011;184:822-7.
30. Curtin-Brosnan J, Paigen B, Hagberg KA, et al. Occupational mouse allergen exposure among non-mouse handlers. Journal of occupational and environmental hygiene 2010;7:726-34.
31. Pacheco KA. New insights into laboratory animal exposures and allergic responses. Current opinion in allergy and clinical immunology 2007;7:156-61.
32. Baxi S, Sheehan WJ, Permaul P, et al. Airborne Fungus Diversity and Concentrations in Inner City Elementary Schools Ped Aller Immunol 2013;24:697-703.
33. Pongracic JA, O'Connor GT, Muilenberg ML, et al. Differential effects of outdoor versus indoor fungal spores on asthma morbidity in inner-city children. The Journal of allergy and clinical immunology 2010;125:593-9.
34. Lignell U, Meklin T, Putus T, et al. Effects of moisture damage and renovation on microbial conditions and pupils' health in two schools--a longitudinal analysis of five years. J Environ Monit 2007;9:225-33.
35. Kioumourtzoglou MA, Coull BA, Dominici F, Koutrakis P, Schwartz J, Suh H. The impact of source contribution uncertainty on the effects of source-specific PM on hospital admissions: A case study in Boston, MA. Journal of exposure science & environmental epidemiology 2014.
36. O'Connor GT, Neas L, Vaughn B, et al. Acute respiratory health effects of air pollution on children with asthma in US inner cities. The Journal of allergy and clinical immunology 2008;121:1133-9 e1.
37. Brunekreef B, Beelen R, Hoek G, et al. Effects of long-term exposure to traffic-related air pollution on respiratory and cardiovascular mortality in the Netherlands: the NLCS-AIR study. Res Rep Health Eff Inst 2009:5-71; discussion 3-89.
38. Permaul P, Hoffman E, Fu C, et al. Allergens in urban schools and homes of children with asthma. Pediatr Allergy Immunol 2012.
39. Fletcher DJ, Lewis SM, Matthews JN. Factorial designs for crossover clinical trials. Statistics in medicine 1990;9:1121-9.
40. Simon R. Designs for efficient clinical trials. Oncology (Williston Park) 1989;3:43-9; discussion 51-3.
41. Niemeijer NR, de Monchy JG. Age-dependency of sensitization to aero-allergens in asthmatics. Allergy 1992;47:431-5.
42. Schwartz J, Weiss ST. Relationship of skin test reactivity to decrements in pulmonary function in children with asthma or frequent wheezing. American journal of respiratory and critical care medicine 1995;152:2176-80.
43. Liccardi G, Cazzola M, D'Amato M, D'Amato G. Pets and cockroaches: two increasing causes of respiratory allergy in indoor environments. Characteristics of airways sensitization and prevention strategies. Respiratory medicine 2000;94:1109-18.
44. Arruda LK, Ferriani VP, Vailes LD, Pomes A, Chapman MD. Cockroach allergens: environmental distribution and relationship to disease. Current Allergy and Asthma Reports 2001;1:466-73.
45. Arshad SH. Indoor allergen exposure in the development of allergy and asthma. Current Allergy and Asthma Reports 2003;3:115-20.
46. Chew GL, Perzanowski MS, Miller RL, et al. Distribution and determinants of mouse allergen exposure in low-income New York City apartments. Environmental health perspectives 2003;111:1348-51.
47. Chew GL, Correa JC, Perzanowski MS. Mouse and cockroach allergens in the dust and air in northeastern United States inner-city public high schools. Indoor Air 2005;15:228-34.
48. Matsui EC, Simons E, Rand C, et al. Airborne mouse allergen in the homes of inner-city children with asthma. Journal of Allergy & Clinical Immunology 2005;115:358-63.
49. Perry T, Matsui E, Merriman B, Duong T, Eggleston P. The prevalence of rat allergen in inner-city homes and its relationship to sensitization and asthma morbidity. Journal of Allergy & Clinical Immunology 2003;112:346-52.

50. Phipatanakul W, Eggleston PA, Wright EC, Wood RA, The National Coooperative Inner-City Asthma S. Mouse allergen. II. The relationship of mouse allergen exposure to mouse sensitization and asthma morbidity in inner-city children with asthma. Journal of Allergy & Clinical Immunology 2000;106:1075-80.
51. Phipatanakul W, Eggleston PA, Wright EC, Wood RA. Mouse allergen. I. The prevalence of mouse allergen in inner-city homes. The National Cooperative Inner-City Asthma Study. The Journal of allergy and clinical immunology 2000;106:1070-4.
52. Phipatanakul W. Rodent allergens. Current Allergy and Asthma Reports 2002;2:412-6.
53. Findley S, Lawler K, Bindra M, Maggio L, Penachio MM, Maylahn C. Elevated asthma and indoor environmental exposures among Puerto Rican children of East Harlem. Journal of Asthma 2003;40:557-69.
54. Simoni M, Lombardi E, Berti G, et al. Mould/dampness exposure at home is associated with respiratory disorders in Italian children and adolescents: the SIDRIA-2 Study. Occupational and environmental medicine 2005;62:616-22.
55. Matheson MC, Abramson MJ, Dharmage SC, et al. Changes in indoor allergen and fungal levels predict changes in asthma activity among young adults. Clin Exp Allergy 2005;35:907-13.
56. Targonski PV, Persky VW, Ramekrishnan V. Effect of environmental molds on risk of death from asthma during the pollen season. The Journal of allergy and clinical immunology 1995;95:955-61.
57. Wallace LA, Mitchell H, O'Connor GT, et al. Particle concentrations in inner-city homes of children with asthma: the effect of smoking, cooking, and outdoor pollution. Environmental health perspectives 2003;111:1265-72.
58. Delfino RJ, Quintana PJ, Floro J, et al. Association of FEV1 in asthmatic children with personal and microenvironmental exposure to airborne particulate matter. Environmental health perspectives 2004;112:932-41.
59. Koenig JQ, Mar TF, Allen RW, et al. Pulmonary effects of indoor- and outdoor-generated particles in children with asthma. Environmental health perspectives 2005;113:499-503.
60. Platts-Mills TA, Vaughan JW, Carter MC, Woodfolk JA. The role of intervention in established allergy: avoidance of indoor allergens in the treatment of chronic allergic disease. Journal of Allergy and Clinical Immunology 2000;106:787-804.
61. Shapiro GG, Wighton TG, Chinn T, et al. House dust mite avoidance for children with asthma in homes of low-income families. Journal of Allergy and Clinical Immunology 1999;103:1069-74.
62. Vojta PJ, Randels SP, Stout J, et al. Effects of physical interventions on house dust mite allergen levels in carpet, bed, and upholstery dust in low-income, urban homes. Environmental health perspectives 2001;109:815-9.
63. Gergen PJ, Mortimer KM, Eggleston PA, et al. Results of the National Cooperative Inner-City Asthma Study (NCICAS) environmental intervention to reduce cockroach allergen exposure in inner-city homes. Journal of Allergy and Clinical Immunology 1999;103:501-6.
64. Wood RA. Environmental control in the prevention and treatment of pediatric allergic disease. Current opinion in pediatrics 1993;5:692-5.
65. Custovic A, Taggart SC, Francis HC, Chapman MD, Woodcock A. Exposure to house dust mite allergens and the clinical activity of asthma. Journal of Allergy & Clinical Immunology 1996;98:64-72.
66. Platts-Mills TA, Ward GW, Jr., Sporik R, Gelber LE, Chapman MD, Heymann PW. Epidemiology of the relationship between exposure to indoor allergens and asthma. International Archives of Allergy and Applied Immunology 1991;94:339-45.
67. Htut T, Higenbottam TW, Gill GW, Darwin R, Anderson PB, Syed N. Eradication of house dust mite from homes of atopic asthmatic subjects: a double-blind trial. Journal of Allergy and Clinical Immunology 2001;107:55-60.
68. Phipatanakul W. Animal allergens and their control. Current Allergy and Asthma Reports 2001;1:461-5.
69. Phipatanakul W. Environmental indoor allergens. Pediatric Annals 2003;32:40-8.
70. Bollinger ME, Eggleston PA, Flanagan E, Wood RA. Cat antigen in homes with and without cats may induce allergic symptoms. Journal of Allergy and Clinical Immunology 1996;97:907-14.
71. Wood RA, Chapman MD, Adkinson NF, Jr., Eggleston PA. The effect of cat removal on allergen content in household-dust samples. Journal of Allergy and Clinical Immunology 1989;83:730-4.

72. Kattan M, Stearns SC, Crain EF, et al. Cost-effectiveness of a home-based environmental intervention for inner-city children with asthma. The Journal of allergy and clinical immunology 2005;116:1058-63.
73. Mitchell H, Cohn RD, Wildfire J, et al. Implementation of Evidence-based Asthma Interventions in Post-Katrina New Orleans: The Head-off Environmental Asthma in Louisiana (HEAL) Study. Environmental health perspectives 2012;120:1607-12.
74. Woods ER, Bhaumik U, Sommer SJ, et al. Community asthma initiative: evaluation of a quality improvement program for comprehensive asthma care. Pediatrics 2012;129:465-72.
75. Howden-Chapman P, Pierse N, Nicholls S, et al. Effects of improved home heating on asthma in community dwelling children: randomised controlled trial. BMJ 2008;337:a1411.
76. Karlsson AS, Renstrom A, Hedren M, Larsson K. Allergen avoidance does not alter airborne cat allergen levels in classrooms. Allergy 2004;59:661-7.
77. Karlsson AS, Andersson B, Renstrom A, Svedmyr J, Larsson K, Borres MP. Airborne cat allergen reduction in classrooms that use special school clothing or ban pet ownership. The Journal of allergy and clinical immunology 2004;113:1172-7.
78. Karlsson AS, Renstrom A, Hedren M, Larsson K. Comparison of four allergen-sampling methods in conventional and allergy prevention classrooms. Clin Exp Allergy 2002;32:1776-81.
79. Pilotto LS, Nitschke M, Smith BJ, et al. Randomized controlled trial of unflued gas heater replacement on respiratory health of asthmatic schoolchildren. International journal of epidemiology 2004;33:208-14.
80. Morales E, Bustamante M, Vilahur N, et al. DNA hypomethylation at ALOX12 is associated with persistent wheezing in childhood. American journal of respiratory and critical care medicine 2012;185:937-43.
81. Baron RM, Kenny DA. The moderator-mediator variable distinction in social psychological research: conceptual, strategic, and statistical considerations. Journal of personality and social psychology 1986;51:1173-82.
82. Vanderweele TJ, Vansteelandt S. Odds ratios for mediation analysis for a dichotomous outcome. Am J Epidemiol 2010;172:1339-48.
83. Kattan M, Crain EF, Steinbach S, et al. A randomized clinical trial of clinician feedback to improve quality of care for inner-city children with asthma. Pediatrics 2006;117:e1095-103.
84. Redline S, Gruchalla RS, Wolf RL, et al. Development and validation of school-based asthma and allergy screening questionnaires in a 4-city study. Annals of allergy, asthma & immunology : official publication of the American College of Allergy, Asthma, & Immunology 2004;93:36-48.
85. Position Statement: Allergen Skin Testing. Journal of Allergy and Clinical Immunology 1993;92:636-7.
86. Dweik R, Boggs P, Erzurum S, et al. An official ATS clinical practice guideline: Interpretation of exhaled nitric oxide levels (FENO) for clinical applications. Am J Respir Crit Care Med 2011;184:602-15.
87. NIH.NHLBI. Guidelines for Diagnosis and Management of Asthma: Expert Panel Report 2. 2002:45-6.
88. Lambrecht BN, Hammad H. The airway epithelium in asthma. Nat Med 2012;18:684-92.
89. Lambrecht BN, Hammad H. Asthma: The importance of dysregulated barrier immunity. Eur J Immunol 2013;43:3125-37.
90. Luttmann-Gibson H, Sarnat SE, Suh HH, et al. Short-term effects of air pollution on oxygen saturation in a cohort of senior adults in Steubenville, Ohio. Journal of occupational and environmental medicine / American College of Occupational and Environmental Medicine 2014;56:149-54.
91. Wylie BJ, Coull BA, Hamer DH, et al. Impact of biomass fuels on pregnancy outcomes in central East India. Environmental health : a global access science source 2014;13:1.
92. Anway MD, Cupp AS, Uzumcu M, Skinner MK. Epigenetic transgenerational actions of endocrine disruptors and male fertility. Science (New York, NY) 2005;308:1466-9.
93. Richards E. Inherited epigenetic variation — revisiting soft inheritance. Nat Rev Genet 2006;7:395-401.
94. Dolinoy DC, Weidman JR, Jirtle RL. Epigenetic gene regulation: Linking early developmental environment to adult disease. . Reprod Toxicol 2006.

95. Rager JE, Bauer RN, Muller LL, et al. DNA methylation in nasal epithelial cells from smokers: identification of ULBP3-related effects. Am J Physiol Lung Cell Mol Physiol 2013;305:L432-8.
96. Beane J, Sebastiani P, Liu G, Brody JS, Lenburg ME, Spira A. Reversible and permanent effects of tobacco smoke exposure on airway epithelial gene expression. Genome Biol 2007;8:R201.
97. Baccarelli A, Wright RO, Bollati V, et al. Rapid DNA methylation changes after exposure to traffic particles. American journal of respiratory and critical care medicine 2009;179:572-8.
98. Baccarelli A, Rusconi F, Bollati V, et al. Nasal cell DNA methylation, inflammation, lung function and wheezing in children with asthma. Epigenomics 2012;4:91-100.
99. Bellavia A, Urch B, Speck M, et al. DNA hypomethylation, ambient particulate matter, and increased blood pressure: findings from controlled human exposure experiments. Journal of the American Heart Association 2013;2:e000212.
100. Madrigano J, Baccarelli A, Mittleman MA, et al. Air pollution and DNA methylation: interaction by psychological factors in the VA Normative Aging Study. Am J Epidemiol 2012;176:224-32.
101. Guo L, Byun HM, Zhong J, et al. Effects of short-term exposure to inhalable particulate matter on DNA methylation of tandem repeats. Environmental and molecular mutagenesis 2014;55:322-35.
102. Hou L, Zhang X, Zheng Y, et al. Altered methylation in tandem repeat element and elemental component levels in inhalable air particles. Environmental and molecular mutagenesis 2014;55:256-65.
103. Tarantini L, Bonzini M, Apostoli P, et al. Effects of particulate matter on genomic DNA methylation content and iNOS promoter methylation. Environmental health perspectives 2009;117:217-22.
104. Sofer T, Baccarelli A, Cantone L, et al. Exposure to airborne particulate matter is associated with methylation pattern in the asthma pathway. Epigenomics 2013;5:147-54.
105. Byun HM, Motta V, Panni T, et al. Evolutionary age of repetitive element subfamilies and sensitivity of DNA methylation to airborne pollutants. Particle and fibre toxicology 2013;10:28.
106. Tarantini L, Bonzini M, Tripodi A, et al. Blood hypomethylation of inflammatory genes mediates the effects of metal-rich airborne pollutants on blood coagulation. Occupational and environmental medicine 2013;70:418-25.
107. Peluso M, Bollati V, Munnia A, et al. DNA methylation differences in exposed workers and nearby residents of the Ma Ta Phut industrial estate, Rayong, Thailand. International journal of epidemiology 2012;41:1753-60; discussion 61-3.
108. Breton CV, Byun HM, Wang X, Salam MT, Siegmund K, Gilliland FD. DNA methylation in the arginase-nitric oxide synthase pathway is associated with exhaled nitric oxide in children with asthma. American journal of respiratory and critical care medicine 2011;184:191-7.
109. Poole A, Urbanek C, Eng C, et al. Dissecting childhood asthma with nasal transcriptomics distinguishes subphenotypes of disease. The Journal of allergy and clinical immunology 2014;133:670-8 e12.
110. Zook PM, Jordan C, Adams B, et al. Retention strategies and predictors of attrition in an urban pediatric asthma study. Clin Trials 2010;7:400-10.
111. Goldstein H. Multilevel statistical models. 3rd ed. London: Arnold; 2003.
112. Fitzmaurice GM LN, Ware JH. Applied Longitudinal Analysis, 2nd Edition. New York: Wiley; 2011.
113. G.M. F, Laird NM, Ware JH, eds. Applied Longitudinal Analysis: Wiley & Sons; 2011.
114. Benjamini Y, Hochberg Y. Controlling the false discovery rate: a practical and powerful approach to multiple testing. Journal of the Royal Statistical Society, Series B 1995;57:289-300.
115. Hedley AA, Ogden CL, Johnson CL, Carroll MD, Curtin LR, Flegal KM. Prevalence of Overweight and Obesity Among US Children, Adolescents, and Adults, 1999-2002. JAMA: The Journal of the American Medical Association 2004;291:2847-50.
116. Bind M-A, Vanderweele T, Coull B, Schwartz J. Mixed effects causal mediation analysis for longitudinal data. Submitted to Biostatistics 2014.

117. Pidsley R, CC YW, Volta M, Lunnon K, Mill J, Schalkwyk LC. A data-driven approach to preprocessing Illumina 450K methylation array data. BMC Genomics 2013;14:293.
118. Leek JT, Storey JD. Capturing heterogeneity in gene expression studies by surrogate variable analysis. PLoS genetics 2007;3:1724-35.
119. Liu Y, Aryee MJ, Padyukov L, et al. Epigenome-wide association data implicate DNA methylation as an intermediary of genetic risk in rheumatoid arthritis. Nature biotechnology 2013;31:142-7.
120. Cohen J. Statistical Power Analysis for the Behavioral Sciences. second ed: Lawrence Erlbaum Associates; 1988.
121. McConnell R, Islam T, Shankardass K, et al. Childhood incident asthma and traffic-related air pollution at home and school. Environmental health perspectives 2010;118:1021-6.
122. CAMP. Long-term effects of budesonide or nedocromil in children with asthma. The Childhood Asthma Management Program Research Group. N Engl J Med 2000;343:1054-63.
123. Asthma prevalence, disease characteristics, and self-management education — United States, 2001–2009. Morbidity and Mortality Weekly Report 2011;60.
124. Gottlieb DJ, Beiser AS, O'Connor GT. Poverty, race, and medication use are correlates of asthma hospitalization rates. A small area analysis in Boston. Chest 1995;108:28-35.
125. Houseman EA, Accomando WP, Koestler DC, et al. DNA methylation arrays as surrogate measures of cell mixture distribution. BMC bioinformatics 2012;13:86.
126. Ungar WJ, ed. Economic Evaluation in Child Health. New York: Oxford University Press Inc; 2010.
127. Akinbami LJ, Sullivan SD, Campbell JD, et al. Asthma outcomes: healthcare utilization and costs. The Journal of allergy and clinical immunology 2012;129:S49-64.
128. Paltiel AD, Fuhlbrigge AL, Kitch BT, et al. Cost-effectiveness of inhaled corticosteroids in adults with mild-to-moderate asthma: results from the asthma policy model. The Journal of allergy and clinical immunology 2001;108:39-46.
129. Braithwaite RS, Meltzer DO, King JT, Jr., Leslie D, Roberts MS. What does the value of modern medicine say about the $50,000 per quality-adjusted life-year decision rule? Med Care 2008;46:349-56.
