# Supplementary material for: Air Purifier Intervention for Respiratory Viral Exposure in Elementary Schools: A Secondary Analysis of a Randomized Clinical Trial
Source: JAMA Netw Open. 2025 Oct 10;8(10):e2536951. doi: 10.1001/jamanetworkopen.2025.36951 (PMC12514627; doi:10.1001/jamanetworkopen.2025.36951)
Supplement: Supplement 2. — eTable 1. Detailed Inclusion and Exclusion Criteria of Parent Study eTable 2. Prevalence and Mean Viral Concentrations by Intervention Group and Period eTable 3. Individual Viral Concentrations in High vs Low Viral Clusters eTable 4. Effect Size of HEPA Intervention on Individual Viral Concentrations eTable 5. Summary of Classrooms With Missing Measures eTable 6. Proportion of Missing Classroom Measure by Cluster eFigure 1. Overview of Study Design Over Each School Year eFigure 2. K-Means Cluster Analysis of Viral Concentrations eFigure 3. Overview of Sample Collection Months Based on Intervention Status and Visit Type eFigure 4. Monthly Trends in Respiratory Viral Exposures Across Classrooms eFigure 5. Comparison of Mean Concentrations of Viruses Between Clusters eFigure 6. Prevalence of High Viral Cluster Based on Season eFigure 7. Receiver Operating Characteristics (ROC) Curves for Elastic Net Regression Model on (A) Training Set and (B) Validation Set eFigure 8. Ranked Variable Importance of Indoor Air Quality and Classroom Characteristics in Predicting High Viral Exposure eFigure 9. Complete Case Analysis Using Elastic Net Model eMethods. eReferences. [file jamanetwopen-e2536951-s002.pdf]

Supplemental Online Content

Sun Y, Haghnazari D, Huang C-Y, et al. Air purifier intervention for respiratory viral exposure in elementary schools: a secondary analysis of a randomized clinical trial. *JAMA Netw Open*. 2025;8(10):e2536951. doi:10.1001/jamanetworkopen.2025.36951

eTable 1. Detailed Inclusion and Exclusion Criteria of Parent Study

eTable 2. Prevalence and Mean Viral Concentrations by Intervention Group and Period

eTable 3. Individual Viral Concentrations in High vs Low Viral Clusters

eTable 4. Effect Size of HEPA Intervention on Individual Viral Concentrations

eTable 5. Summary of Classrooms With Missing Measures

eTable 6. Proportion of Missing Classroom Measure by Cluster

eFigure 1. Overview of Study Design Over Each School Year

eFigure 2. *K*-Means Cluster Analysis of Viral Concentrations

eFigure 3. Overview of Sample Collection Months Based on Intervention Status and Visit Type

eFigure 4. Monthly Trends in Respiratory Viral Exposures Across Classrooms

eFigure 5. Comparison of Mean Concentrations of Viruses Between Clusters

eFigure 6. Prevalence of High Viral Cluster Based on Season

eFigure 7. Receiver Operating Characteristics (ROC) Curves for Elastic Net Regression Model on (A) Training Set and (B) Validation Set

eFigure 8. Ranked Variable Importance of Indoor Air Quality and Classroom Characteristics in Predicting High Viral Exposure

© 2025 Sun Y et al. *JAMA Network Open*

eFigure 9. Complete Case Analysis Using Elastic Net Model

eMethods.

eReferences.

This supplemental material has been provided by the authors to give readers additional information about their work.

eTable 1: Detailed inclusion and exclusion criteria of parent study.

**Inclusion Criteria**	• Subject and/or parent guardian must be able to understand and provide informed consent<br>• Males and females who will be in grades K-8 (age 4-15 at randomization) during the subsequent academic school year after spring screening<br>• Attend one of the schools that study team have permission to obtain classroom/school environmental samples for the subsequent academic school year<br>• Have no plans to move schools within the upcoming 12 months<br>• Have health care insurance and asthma provider<br>• Have physician-diagnosed asthma at least 1 year prior to the screening visit<br>• Have evidence of active asthma disease as defined by at least one of the following:<br>o One asthma-related unscheduled visit to an emergency department (ED), clinic or urgent care facility in the previous 12 months<br>o One asthma-related overnight hospitalization in the previous 12 months<br>o One or more bursts of oral corticosteroids in the previous 12 months<br>o Wheezing symptoms in the past 12 months<br>o On daily controller medications for asthma
**Exclusion Criteria (for student participants)**	• Inability or unwillingness of a participate to give written informed consent or comply with study protocol in the judgement<br>• Inability to perform spirometry<br>• Lung disease, other than asthma, that requires daily medication<br>• Cardiovascular disease that requires daily medication, excluding hypertension<br>• Taking a beta-blocker<br>• Currently receiving unstable level of Immunotherapy (allergy shots)<br>• Switching to a school where staff are not doing environmental sampling for that year
**Exclusion Criteria (for school/classroom)**	• Unable to access areas of school necessary to conduct extermination or place HEPA air purifier<br>• School in extensive state of disrepair/damage as determined by study coordinator/principal investigator (PI)<br>• Presence of centralized air filtration or purifying system (rare in Northeast Schools)

© 2025 Sun Y et al. *JAMA Network Open*

eTable 2: Prevalence and mean viral concentrations by intervention group and period.

	Sham				HEPA			
	Pre-intervention (N = 91)		Post-intervention (N = 147)		Pre-intervention (N = 109)		Post-intervention (N = 185)	
Virus	Prevalence	Mean concentration[a] (SD)	Prevalence	Mean concentration (SD)	Prevalence	Mean concentration (SD)	Prevalence	Mean concentration SD)
Adenovirus	16 (17.6%)	3016.0 (6789.9)	34 (23.1%)	4416.0 (9202.9)	27 (24.8%)	4061.0 (7812.9)	48 (25.9%)	4272.7 (7956.3)
Coronavirus HKU1	10 (11.0%)	303.1 (1791.2)	48 (32.7%)	3095.3 (6965.4)	9 (8.3%)	600.1 (3513.7)	50 (27.0%)	2225.6 (6553.4)
Coronavirus NL63	16 (17.6%)	1850.8 (4757.5)	45 (30.6%)	3715.3 (6643.1)	20 (18.3%)	2128.7 (5410.1)	43 (23.2%)	2251.1 (4959.9)
Coronavirus OC43	18 (19.8%)	3729.1 (9137.7)	92 (62.6%)	12264.5 (11732.7)	25 (22.9%)	4232.7 (8774.8)	84 (45.4%)	8398.0 (10687.6)
Enterovirus	27 (29.7%)	1148.1 (3070.6)	41 (27.9%)	1200.6 (3576.5)	35 (32.1%)	1816.5 (4587.1)	45 (24.3%)	1085.7 (3150.8)
Enterovirus D68	21 (23.1%)	3412.3 (6657.4)	5 (3.4%)	437.1 (2685.9)	21 (19.3%)	2837.5 (6207.0)	8 (4.3%)	538.5 (2613.0)
Influenza A	6 (6.6%)	703.3 (3134.6)	44 (29.9%)	4511.6 (8494.8)	4 (3.7%)	358.3 (1963.6)	40 (21.6%)	3677.0 (7983.8)
Influenza A (H1N1)	4 (4.4%)	435.9 (2940.6)	36 (24.5%)	2516.2 (7148.5)	6 (5.5%)	477.4 (2496.3)	26 (14.1%)	1606.1 (5010.4)
Influenza A (H3N2)	2 (2.2%)	329.2 (2214.2)	25 (17.0%)	2630.0 (6564.6)	3 (2.8%)	271.4 (2011.7)	27 (14.6%)	2579.0 (7051.7)
Influenza B	5 (5.5%)	311.7 (2119.4)	31 (21.1%)	3025.0 (7357.7)	6 (5.5%)	474.0 (2556.8)	34 (18.4%)	2734.0 (6543.7)
Metapneumovirus	7 (7.7%)	253.0 (1594.4)	34 (23.1%)	2844.8 (6408.1)	11 (10.1%)	622.0 (2673.9)	38 (20.5%)	1820.9 (5423.7)
Parainfluenza virus 1	7 (7.7%)	1414.0 (5266.8)	5 (3.4%)	494.3 (2645.2)	5 (4.6%)	706.0 (3370.6)	4 (2.2%)	306.6 (2102.9)
Parainfluenza virus 2	12 (13.2%)	137.0 (1221.4)	13 (8.8%)	38.1 (354.6)	12 (11.0%)	13.3 (113.1)	10 (5.4%)	3.9 (52.5)
Parainfluenza virus 3	12 (13.2%)	977.8 (4039.0)	36 (24.5%)	4202.6 (8558.3)	16 (14.7%)	1005.5 (4173.6)	32 (17.3%)	1684.8 (5344.9)
Parainfluenza virus 4	24 (26.4%)	3635.8 (7544.3)	9 (6.1%)	541.6 (2563.8)	22 (20.2%)	2202.7 (6575.1)	4 (2.2%)	332.1 (2330.1)
Parechovirus	1 (1.1%)	173.8 (1658.0)	2 (1.4%)	226.7 (1956.7)	4 (3.7%)	347.8 (2115.7)	1 (0.5%)	0.0 (0.0)
Respiratory Syncytial Virus A	2 (2.2%)	223.5 (1516.9)	32 (21.8%)	3965.4 (8851.0)	3 (2.8%)	500.8 (3133.0)	29 (15.7%)	2552.5 (6638.0)
Respiratory Syncytial Virus B	7 (7.7%)	1016.1 (4328.2)	54 (36.7%)	6606.0 (9771.2)	13 (11.9%)	1725.9 (5446.6)	53 (28.6%)	4636.1 (8148.2)
Rhinovirus	84 (92.3%)	18578.1 (8923.3)	135 (91.8%)	16927.1 (9433.3)	101 (92.7%)	18425.5 (8732.7)	156 (84.3%)	13831.7 (9744.2)

[a] Mean concentration in viral copies/m^3. Note that some viruses are known to have strong seasonal variation. All pre-intervention samples were collected during the months of October – December, while post-intervention samples were collected from January – June.
Abbreviations: HEPA, high-efficiency particulate air; SD, standard deviation.

© 2025 Sun Y et al. *JAMA Network Open*

eTable 3: Individual viral concentrations in high vs low viral clusters

Virus [a]	High Viral Cluster (N = 118)	Low Viral Cluster (N = 414)	*P*-value [b]
Adenovirus	8294.7 (10989.4)	2845.2 (6606.2)	<0.001
Coronavirus HKU1	6513.5 (9965.4)	461.7 (2342.6)	<0.001
Coronavirus NL63	5657.3 (7405.0)	1679.9 (4557.8)	<0.001
Coronavirus OC43	17133.5 (11607.0)	5158.1 (9060.7)	<0.001
Enterovirus	1033.8 (3344.4)	1347.4 (3662.0)	0.40
Enterovirus D68	498.3 (2678.8)	1750.9 (5003.7)	0.009
Influenza A	8852.6 (11266.5)	970.8 (3347.6)	<0.001
Influenza A (H1N1)	3157.9 (7982.7)	932.6 (3808.0)	<0.001
Influenza A (H3N2)	6285.6 (9822.6)	438.5 (2487.0)	<0.001
Influenza B	5150.1 (9388.7)	1021.2 (3740.8)	<0.001
Metapneumovirus	3919.6 (7546.2)	926.0 (3625.8)	<0.001
Parainfluenza virus 1	497.5 (2672.3)	667.4 (3408.9)	0.62
Parainfluenza virus 2	152.1 (1138.4)	5.5 (71.0)	0.009
Parainfluenza virus 3	3303.5 (7823.4)	1783.2 (5578.7)	0.02
Parainfluenza virus 4	1082.4 (4062.0)	1411.3 (5078.6)	0.52
Parechovirus	282.5 (2182.1)	129.8 (1338.4)	0.35
Respiratory Syncytial Virus A	7368.3 (10930.4)	629.5 (3038.2)	<0.001
Respiratory Syncytial Virus B	8990.0 (10793.2)	2532.7 (6230.8)	<0.001
Rhinovirus	17317.4 (9462.2)	16190.0 (9517.7)	0.26

[a] Values for each listed virus represent the mean (SD) concentration in copies/m^3.
[b] *P*-value derived using student's T-test.
Abbreviations: SD, standard deviation.

© 2025 Sun Y et al. *JAMA Network Open*

eTable 4: Effect size of HEPA intervention on individual viral concentrations.

Virus [a]	β–estimate (95% CI)	Raw P-value [b]	FDR -adjusted P-value
Parainfluenza virus 3	-0.43 (-0.7 – -0.10)	0.01	0.10
Coronavirus OC43	-0.41 (-0.70 – -0.12)	0.005	0.10
Respiratory Syncytial Virus B	-0.35 (-0.66 – -0.03)	0.03	0.19
Coronavirus NL63	-0.33 (-0.64 – -0.01)	0.04	0.19
Rhinovirus	-0.30 (-0.30 – 0.00)	0.05	0.19
Metapneumovirus	-0.28 (-0.61 – 0.06)	0.10	0.28
Respiratory Syncytial Virus A	-0.27 (-0.59 – 0.04)	0.09	0.28
Parechovirus	-0.26 (-0.61 – 0.09)	0.15	0.32
Enterovirus	-0.22 (-0.57 – 0.12)	0.21	0.36
Coronavirus HKU1	-0.21 (-0.53 – 0.12)	0.21	0.36
Influenza A (H1N1)	-0.19 (-0.52 – 0.14)	0.25	0.40
Adenovirus	-0.15 (-0.47 – 0.18)	0.38	0.46
Influenza B	-0.08 (-0.41 – 0.25)	0.64	0.71
Influenza A	-0.03 (0.33 – 0.28)	0.87	0.90
Influenza A (H3N2)	0.02 (-0.31 – 0.35)	0.90	0.90
Enterovirus D68	0.15 (-0.18 – 0.48)	0.38	0.46
Parainfluenza virus 1	0.16 (-0.19 – 0.50)	0.38	0.46
Parainfluenza virus 2	0.16 (-0.19 – 0.51)	0.37	0.46
Parainfluenza virus 4	0.26 (-0.07 – 0.58)	0.12	0.29

[a] Viruses are ordered from lowest to highest β-estimate. β-estimates represent the change in viral concentration in standard deviation units relative to the mean across all samples

[b] P-value derived from generalized additive mixed effect models.

Abbreviations: HEPA, high efficiency particulate air; FDR, False Discovery Rate (FDR).

© 2025 Sun Y et al. *JAMA Network Open*

eTable 5: Summary of classrooms with missing measures.

Classroom Characteristic [a]	**Classrooms with missing measures (N = 92)**	**Classrooms without missing measures (N = 108)**	**P-value**[b]
Average CO_2	803.2 (698.3 -907.2)	818.4 (691.5 - 912.8)	0.67
$PM_{2.5}$	4.5 (3.6 - 6.0)	4.4 (3.5 - 5.7)	0.79
Coarse PM	6.9 (5.6 - 8.4)	7.0 (5.4 - 9.1)	0.58
Relative Humidity %	42.4 (39.8 - 45.7)	42.0 (39.2 - 46.7)	0.95
Temperature	21.0 (20.3 - 21.7)	20.8 (19.9 - 21.7)	0.22
Air exchange rate	3.2 (3.0 - 3.4)	3.1 (2.9 - 3.4)	0.23
Students per class	19 (17 - 20)	19 (18 - 20)	0.60
Central HVAC	23 (25.0%)	24 (22.2%)	0.78
Classroom Dimensions	241.5 (219.7 - 270.1)	257.8 (229.2 - 298.7)	0.02
Grade	3.0 (2.0 - 5.0)	3.0 (1.8 - 4.3)	0.03
School Characteristics			
Average number of days absent	8.8 (8.1 - 9.8)	9.0 (8.0 - 10.3)	0.10
Total number of students in school	401.0 (294.8 - 545.0)	405.0 (326.0 - 629.0)	0.24
Student demographics			
Male %	50.8 (48.4 - 52.4)	51.0 (50.0 - 52.3)	0.78
Low-income %	57.8 (40.8 - 65.7)	63.2 (50.3 - 69.7)	0.06
Hispanic %	38.4 (29.5 - 71.0)	40.5 (29.0 - 70.0)	0.94
African American %	16.6 (5.0 - 38.4)	26.0 (5.1 - 36.8)	0.17
White %	14.7 (5.1 - 30.7)	5.7 (2.5 - 24.4)	0.01

[a] Summary statistics are presented as median (IQR) for continuous variables and N (%) for categorical variables. Classroom characteristics with repeated measures (CO_2, $PM_{2.5}$, coarse PM, relative humidity, temperature and air exchange rate) are averaged over all measures in the school year, including imputed values.
[b] *P*-values derived using Wilcoxon rank-sum test for continuous variables, and Chi-square testing for categorical variables
Abbreviations: IQR, interquartile range; HEPA, high-efficiency particulate air; HVAC, heat, ventilation, air conditioning; PM, particulate matter; CO_2, carbon dioxide.

eTable 6: Proportion of missing classroom measure by cluster.

	High viral cluster (N = 118)	Low viral cluster (N = 414)	
**Missing Variable**	**Proportion Missing – N (%)**	**Proportion Missing – N (%)**	**P- value[a]**
Classroom dimensions (m^3)	4 (3.4%)	16 (3.9%)	1.00
Carbon dioxide	11 (9.3%)	44 (10.6%)	0.81
Coarse particulate matter ($\mu g/m^3$)	7 (5.9%)	45 (10.9%)	0.16
Fine particulate matter ($\mu g/m^3$)	8 (6.8%)	46 (11.1%)	0.23
Relative humidity (%)	11 (9.3%)	44 (10.6%)	0.81
Temperature (°C)	11 (9.3%)	44 (10.6%)	0.81
Air exchange rate (exchanges/hour)	11 (9.3%)	45 (10.9%)	0.75

[a] *P*-values derived using Wilcoxon rank-sum test for continuous variables

© 2025 Sun Y et al. *JAMA Network Open*

eFigure 1: Overview of study design over each school year.

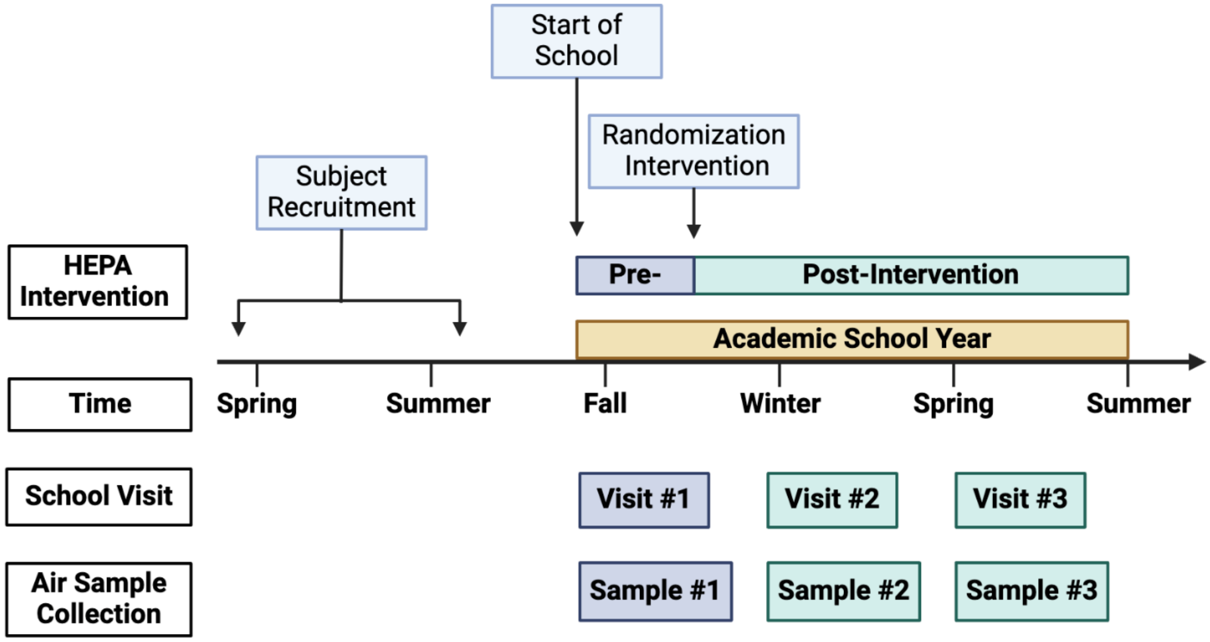

© 2025 Sun Y et al. *JAMA Network Open*

eFigure 2: *K*-means cluster analysis of viral concentrations.

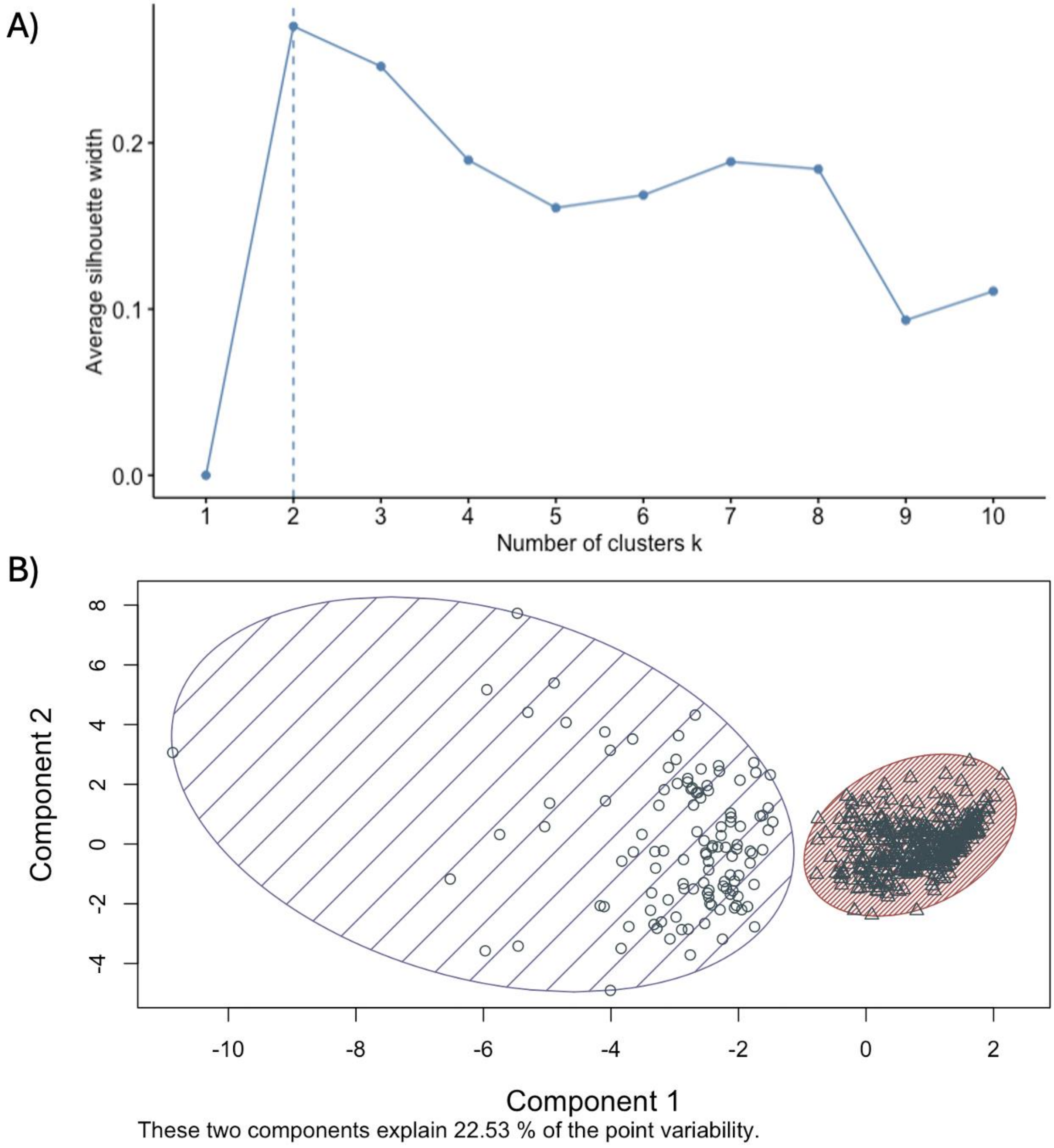

A) K-means clustering showing average silouette width is optimal at K = 2. B) Visualization of viral clusters based on principal component analysis. Each point represents an individual sample, with each circle indicating a different cluster assignment.

© 2025 Sun Y et al. *JAMA Network Open*

eFigure 3: Overview of sample collection months based on intervention status and visit type.

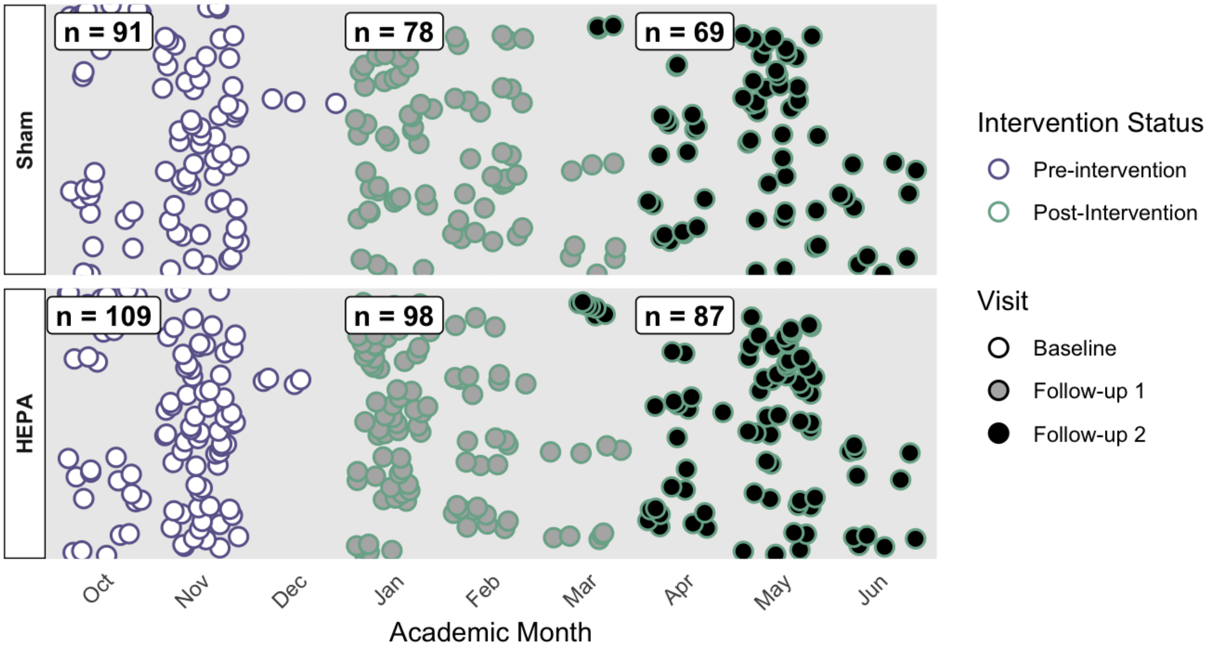

© 2025 Sun Y et al. *JAMA Network Open*

eFigure 4. Monthly trends in respiratory viral exposures across classrooms.

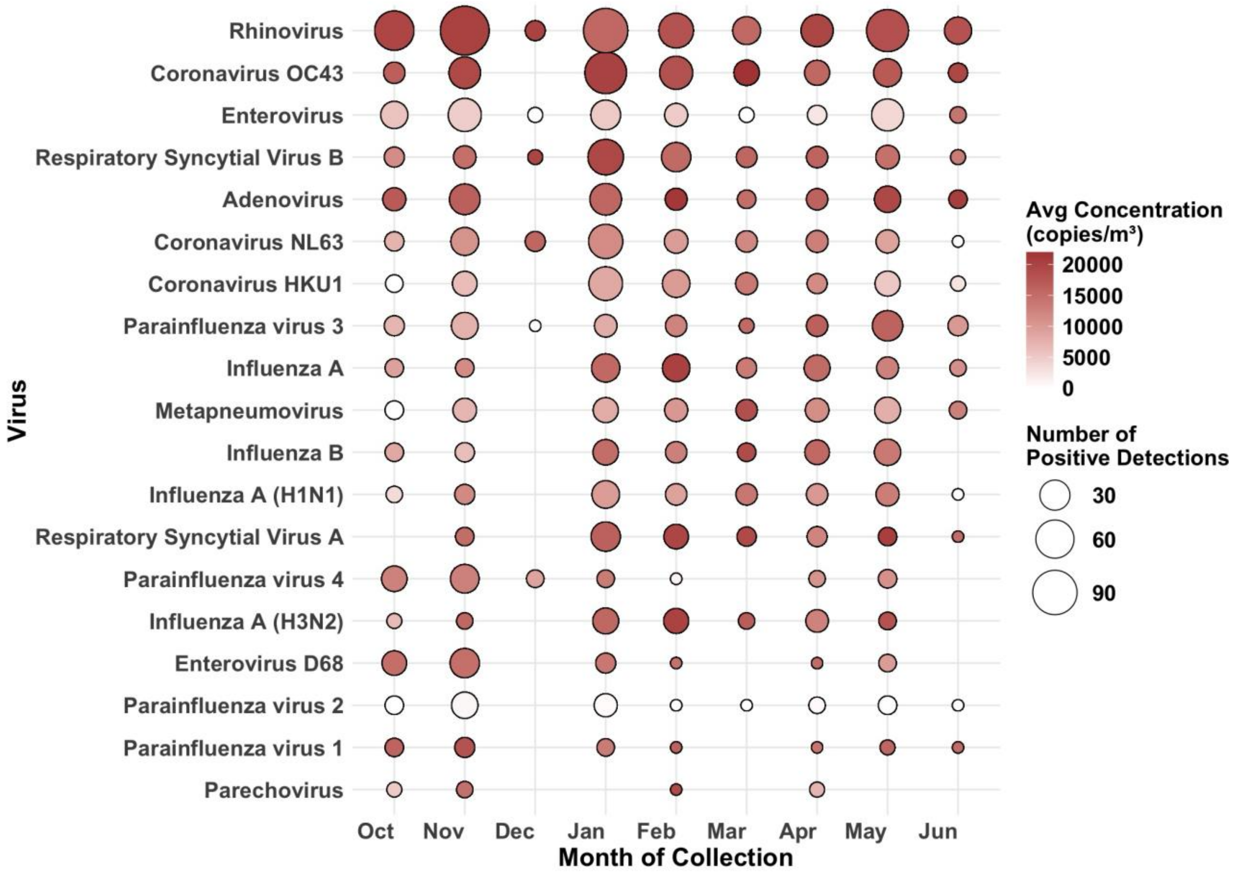

Y-axis shows viruses, arranged form most (top) to least prevalent virus (bottom). X-axis shows academic school months. Circle size indicates the number of positive detections, while the color within each circle reflects the average viral concentration (copies/m³) per month.

© 2025 Sun Y et al. *JAMA Network Open*

eFigure 5: Comparison of mean concentrations of viruses between clusters

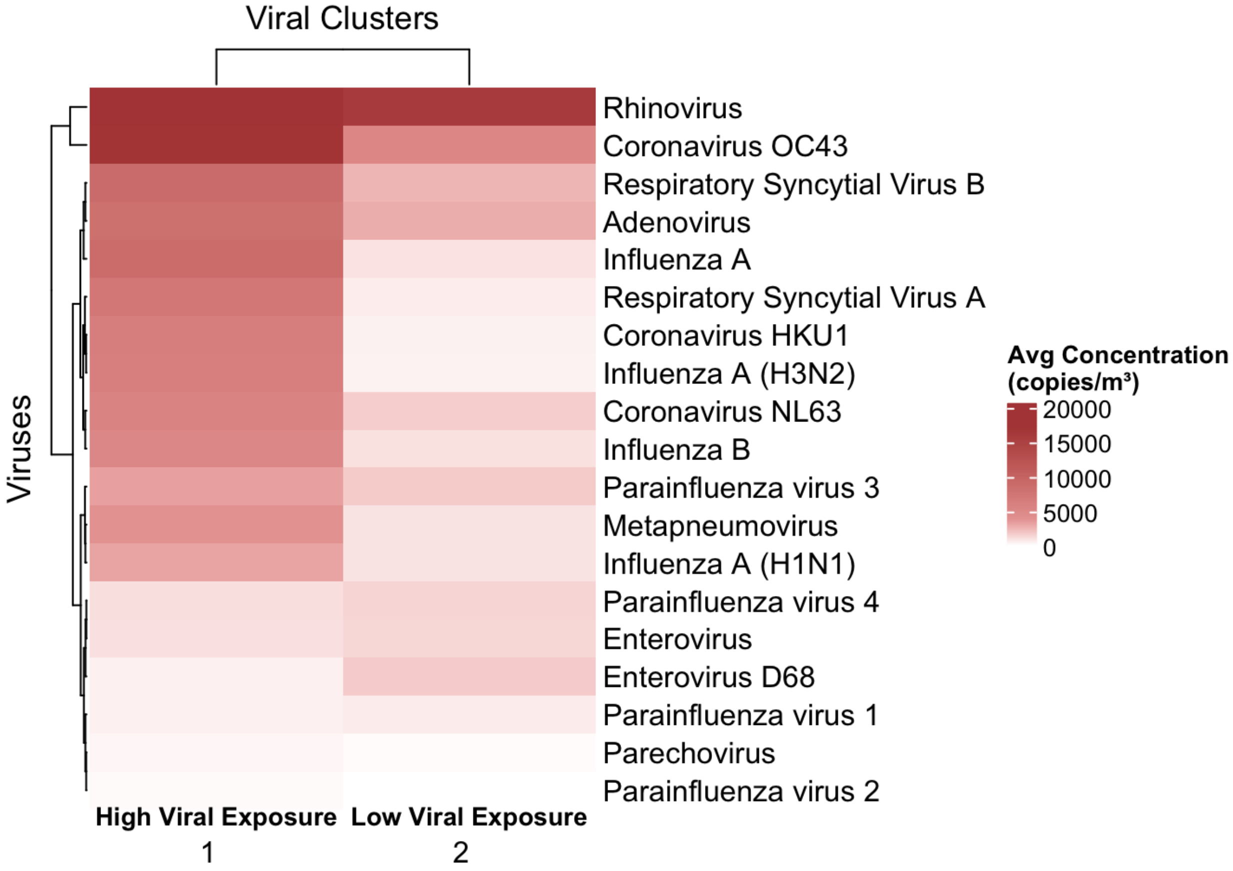

Average viral concentrations for each viral cluster identified based on K-means cluster analysis. Cluster 1 shows overall higher concentration of viruses compared to Cluster 2 and is thus designated as a marker of high global viral exposure.

© 2025 Sun Y et al. *JAMA Network Open*

eFigure 6: Prevalence of high viral cluster based on season.

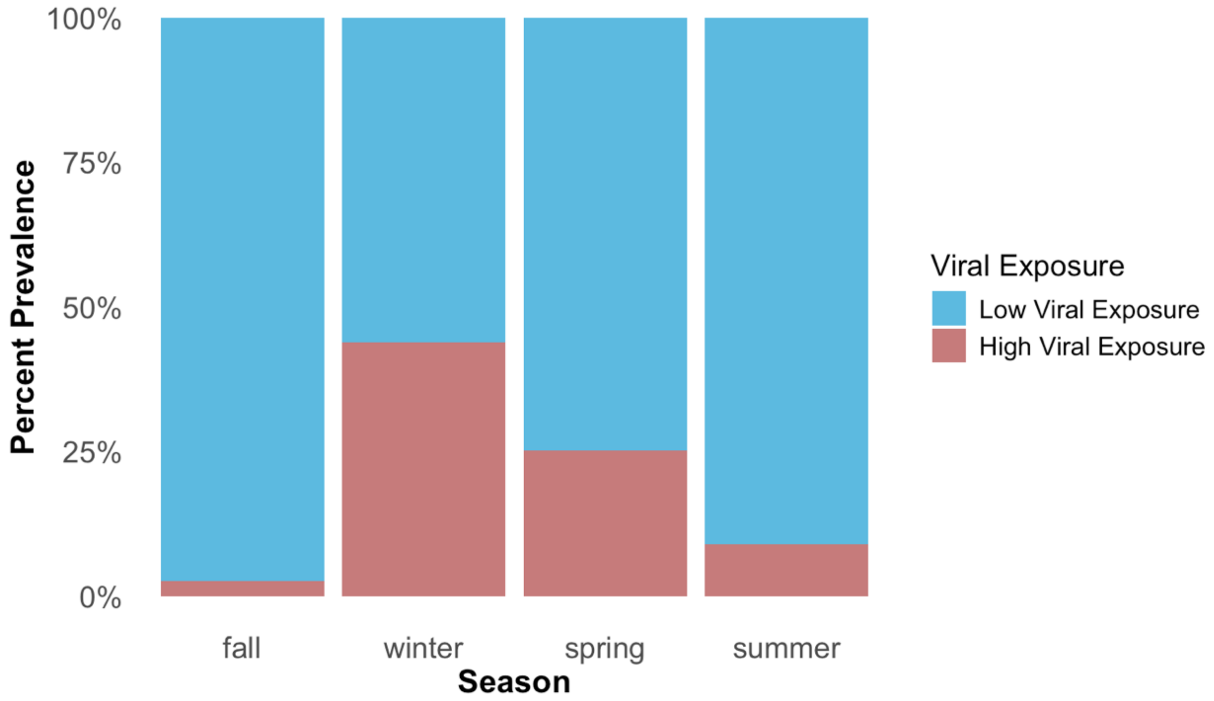

© 2025 Sun Y et al. *JAMA Network Open*

eFigure 7: Receiver Operating Characteristics (ROC) curves for Elastic Net regression model on (A) Training Set and (B) Validation Set.

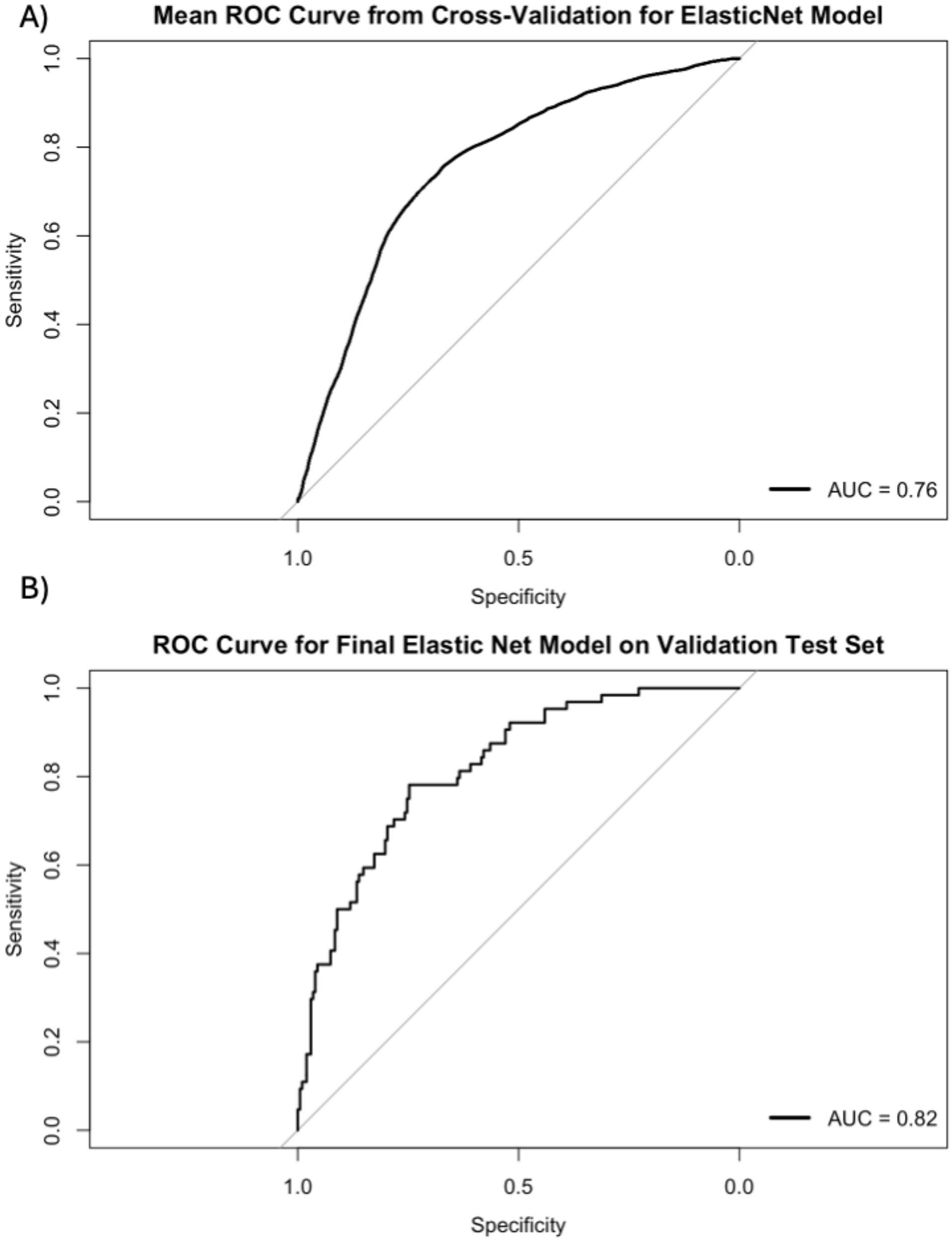

A) Receiver operating characteristics (ROC) curve for Elastic Net regression model derived using repeated 5-fold cross-validation using training data. B) ROC curve for final model tested on validation test set. Area Under the Curve (AUC) values are shown. Training and validation datasets were split 50:50 prior to model building.
Abbreviations: ROC, receiver operating characteristics; AUC, area under the curve.

© 2025 Sun Y et al. *JAMA Network Open*

eFigure 8. Ranked variable importance of indoor air quality and classroom characteristics in predicting high viral exposure.

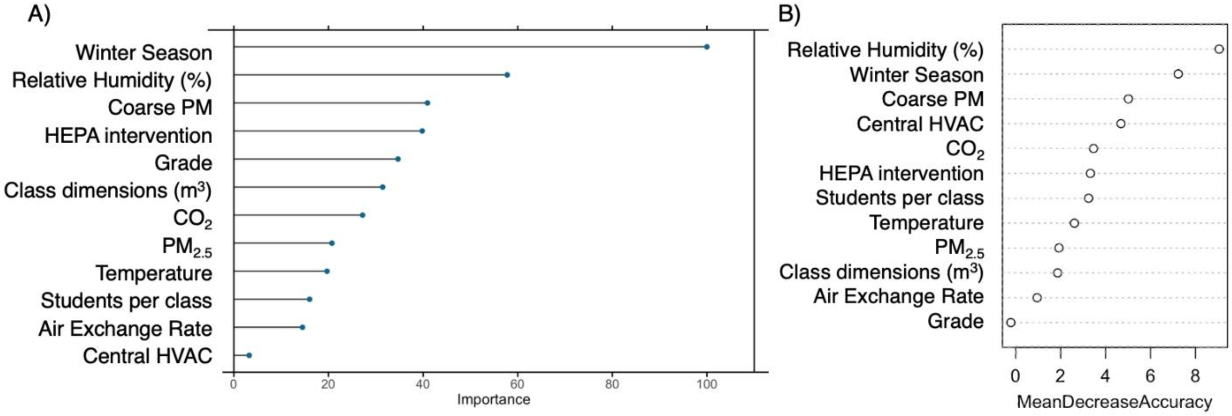

Indoor air quality and classroom characteristics ranked by variable importance in predicting high viral exposure using A) Elastic Net regression and B) Random Forest.
Abbreviations: HEPA, high-efficiency particulate air; HVAC, heat, ventilation, air conditioning; PM, particulate matter; CO_2, carbon dioxide.

© 2025 Sun Y et al. *JAMA Network Open*

eFigure 9. Complete case analysis using Elastic Net model.

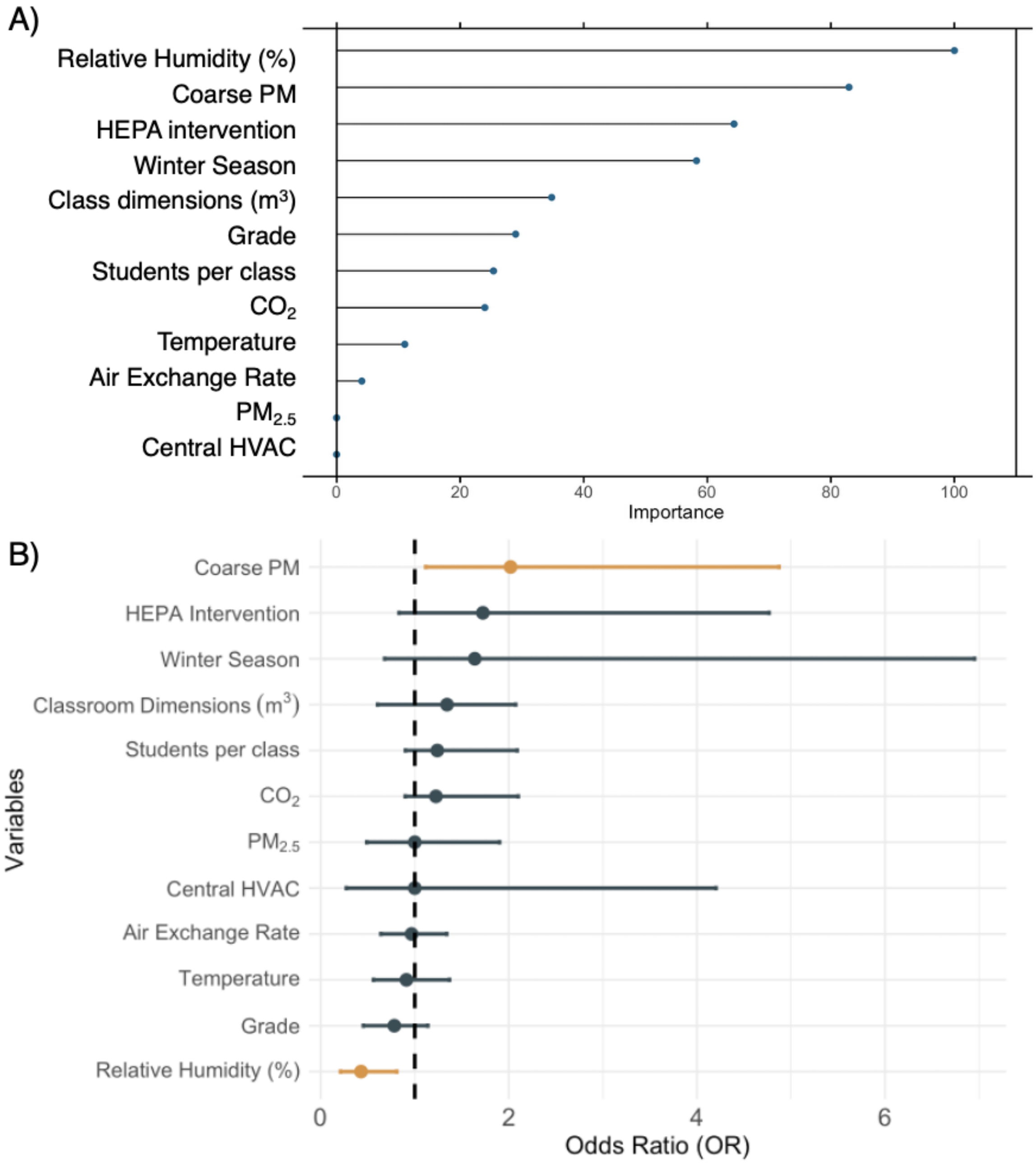

A) Indoor air quality and classroom characteristics ranked by variable importance in predicting high viral exposure using Elastic Net regression using complete cases only. B) Odds Ratios (ORs) of predictive indoor air quality measures and classroom characteristics derived from Elastic Net Regression using complete cases only. Variables are arranged from highest OR (top) to lowest OR (bottom), with 95% confidence intervals (CIs) derived using boostrapping with 2000 resamples are shown. Variables where CIs do not cross 1 are depicted in yellow.

Abbreviations: HEPA, high-efficiency particulate air; HVAC, heat, ventilation, air conditioning; PM, particulate matter; CO_2, carbon dioxide.

© 2025 Sun Y et al. *JAMA Network Open*

eMethods:

Viral bioaerosol sampling:

The bioaerosol samplers were placed 1.3 meters above the floor to approximate the breathing zone of the typical elementary school student. The research team was instructed to place the samplers at least 3 feet away from walls, windows, doors, and the HEPA cleaner. Given the heterogeneity of the classroom layouts and the need to coordinate the placement of the samplers with the homeroom teacher to not interfere with classroom activities, it was not possible to strictly standardize the location of the sampler within each classroom although prior studies have shown that in similarly sized indoor passively ventilated spaces, the air is well mixed with little coefficient of variation when sampling from different locations[1]. Bioaerosol samples were collected using open-faced samplers equipped with the 5 µm pore polytetrafluroethylene (PTFE) filters that were previously heat-treated at 260 °F for 3 hours to eliminate any background nucleic acids and operated at a flow rate of 3 L/min over a one-week period. Field blanks were collected alongside samples to confirm the absence of viral nucleic acid contamination. Unlike polycarbonate filters, PTFE filters have an internal structure featuring a highly porous fibrous network with tortuous pore pathways; this allows for capture of airborne particles smaller than the pore size via multiple mechanisms (inertial impaction, interception, diffusion, gravitational settling) and not solely by simple size exclusion (i.e. mechanical sieving). As shown by Burton et al[2], the choice of PTFE as a filter material is more critical than pore size for viral collection efficiency; no significant difference was observed across PTFE filters with pore sizes of 0.3 µm, 0.5 µm, 1 µm, and 3 µm for virions smaller than 80 nm whereas polycarbonate filters had significantly reduced collection efficiency; smaller pore sizes are also associated with a greater pressure drop across the filter and thus higher risk of pump failure. Soo et al[3] has shown that 5µm PTFE filters have approximately 95% collection efficiency for particles ranging from 10-400nm in size; there was no impact on collection efficiency on variable flow rates ranging from 1.7 to 4.4 liters per minute.

In this study, bioaerosol sampling was conducted throughout active school hours with students and teachers present. The selected filter type and flow rate was optimized to balance viral collection efficiency, minimize noise, and maintain filter and sampling pump integrity throughout the extended sampling period. Upon sampling completion, filters were retrieved and stored at –80°C until nucleic acid extraction.

Indoor Air Quality Measures:

Particulate Matter:

Particulate matter (PM) was measured using two-stage cascade impactors with flow rate of 5 L/min to obtain gravimetric mass concentrations of fine ($PM_{2.5}$) and coarse PM ($PM_{2.5\text{-}10}$). The first impaction stage removes particles with sizes > than 10µm. The second impaction stage collects coarse PM ($PM_{2.5\text{-}10}$). The Teflon filter downstream of the second stage collects $PM_{2.5}$.[4] Cascade samplers were placed in classrooms and ran continuously for one week per sampling period. Pre-weighed Teflon filters collected $PM_{2.5}$ on the lower stage and polyurethane foam substrates collected $PM_{2.5\text{-}10}$ in the upper stage. Filters and polyurethane foam substrates were weighed and conditioned under controlled temperature and humidity before and after sampling. PM concentrations were calculated from weight differences normalized to µg/m^3 of total sampled air volume, which was determined by measuring flow and sampling duration.

Relative Humidity, Carbon Dioxide (CO_2), Temperature:

Sensor-based continuous measurements of relative humidity, CO_2 and temperature (°C) were captured over the one week of environmental sampling in each classroom using Netatmo Smart Home Weather Stations (Netatmo, France). Measurements were obtained in 5-minute intervals. To account for potential sensor malfunctions, values below the 1st percentile or above the 99th percentile were excluded from analysis. Average relative humidity, CO_2, and temperature were calculated over week of sampling, excluding measurements obtained outside of school days. Peak CO_2 is also recorded per sampling period.

Air Exchange Rate:

Air exchange rate, or air changes per hour (ACH), a surrogate marker for ventilation efficiency, was estimated based on rate of decay of CO_2 at the end of the school day.[5–7] First, ambient CO_2 levels were determined for each day and classroom by averaging CO_2 values recorded between 1:00 AM and 4:00 AM to best estimate CO_2 with minimal

activity and room occupancy. Then, rolling windows of consecutive CO_2 measurements over 60 minutes were calculated during school hours for each weekday using the following formula:

$$ACH = \frac{\ln\left(\frac{CO_{2\,(initial)} - CO_{2\,(ambient)}}{CO_{2\,(end)} - CO_{2\,(ambient)}}\right)}{\Delta\, time}$$

This represents the exponential rate of decay for CO_2 over 60 minutes. For each day of sampling in each classroom, the maximum ACH value was calculated. These daily values were then averaged over the week of sampling in order to determine the air exchange rate for each classroom sample.

HEPA intervention:

Active high efficiency particulate air (HEPA) filters were modified by installing ports to measure pressure drop (ΔP) using a Magnahelic gage (range 0.000 to 0.500 inches of water). After, sham filters were modified by first removing the HEPA filter and then installing noise generators (to simulate sound of active air purifiers). Prior to installation in classrooms, fresh HEPA filters were placed inside all active air purifiers. Pressure drop was measured with the fresh filters installed and recorded. All HEPA filters had an equivalent Minimum Efficiency Reporting Value (MERV) rating of 17. We verified that the number of portable HEPA cleaners per classroom was sized appropriately. Each of the portable HEPA cleaners had a flow of 106 CFM. The typical classroom size was about 400 square feet, with a ceiling height of about 10 feet, with an average volume of 4000 cubic feet. The total flow of four portable HEPA cleaners was then 424 cubic feet per minute or 25440 cubic feet per hour, resulting in an average 6.4 air changes per hour (ACH). Centers for Disease Control and Prevention (CDC) guidance has recommended targeting a minimum 5 equivalent ACH for reducing viral exposure[8].

After randomization, the four portable HEPA cleaners were placed in the middle of each of the classroom walls, equidistant to each other. The HEPA cleaners were left on at all times; the on/off buttons for the HEPA cleaners were disabled thus the only way to turn them off was to unplug them. During each school visit, a research assistant assessed the units to document functionality, whether the unit was plugged in at the time of the school visit. Reminders are taped on the units to encourage compliance. If the units were unplugged, study staff communicated with the school contacts to encourage compliance. The air filters were changed quarterly and checked throughout the year to ensure pressure drop was consistent throughout the school year. The flow of each air cleaner was monitored by measuring the pressure drop across the filter. The initial flow was verified prior to initial deployment, and measured before each filter change, after each filter change, and at the end of the school year. The flow decreases as particles collect on the filter, but the maximum decrease over each of the three deployment periods for all filters was about 15%.

In our primary outcomes paper for the clinical trial published in 2021[9] we have reported that the HEPA intervention led to a 2.2 (1.4 – 3.0) $\mu g/m^3$ decrease in coarse particulate matter and 1.7 (0.9 – 2.5) $\mu g/m^3$ decrease in fine particulate matter, indicating that the intervention was effective in reducing classroom indoor air particulate matter.

Imputation of Missing Variables:

Imputation was not performed for the primary intention-to-treat analysis. For robust predictive modeling using machine learning in order to determine which classroom features and indoor air quality measures were most predictive of high viral exposure, missing data in the predictor variables were imputed. There was an overall rate of missingness of 22.4%. Missing variables included classroom dimension (m^3) (3.8% missing), indoor air quality measures including relative humidity, CO_2, temperature and air exchange rate (10.3% missing), $PM_{2.5}$ (9.8%), and coarse PM (10.2%). Missingness primary resulted from equipment or deployment failures (e.g. air sensors not deploying correctly or measures not recorded) and was therefore assumed to be missing completely at random. Given this pattern of missingness, we then selected K-nearest neighbors (KNN) imputation. This method replaces missing values with the average of the *K* most similar observations (nearest neighbors) based on Euclidean distance.

© 2025 Sun Y et al. *JAMA Network Open*

To determine the optimal value of *K*, we conducted a simulated analysis in which we artificially introduced 20% missingness to our complete dataset, mirroring our overall missingness rate of 22.4%. To account for the randomness in missing data patterns, we repeated this process across 100 iterations, each with different randomly generated missingness pattern. For each iteration, we applied KNN imputation using K values ranging from 1-20 and calculated root mean square error (RSME) between the imputed and true values. Average RSME across these 100 simulations were then calculated. Using the elbow method, we then identified *K* = 5 as the optimal value, as RSME values plateaued beyond this point, indicating limited additional benefit from larger *K*-values.

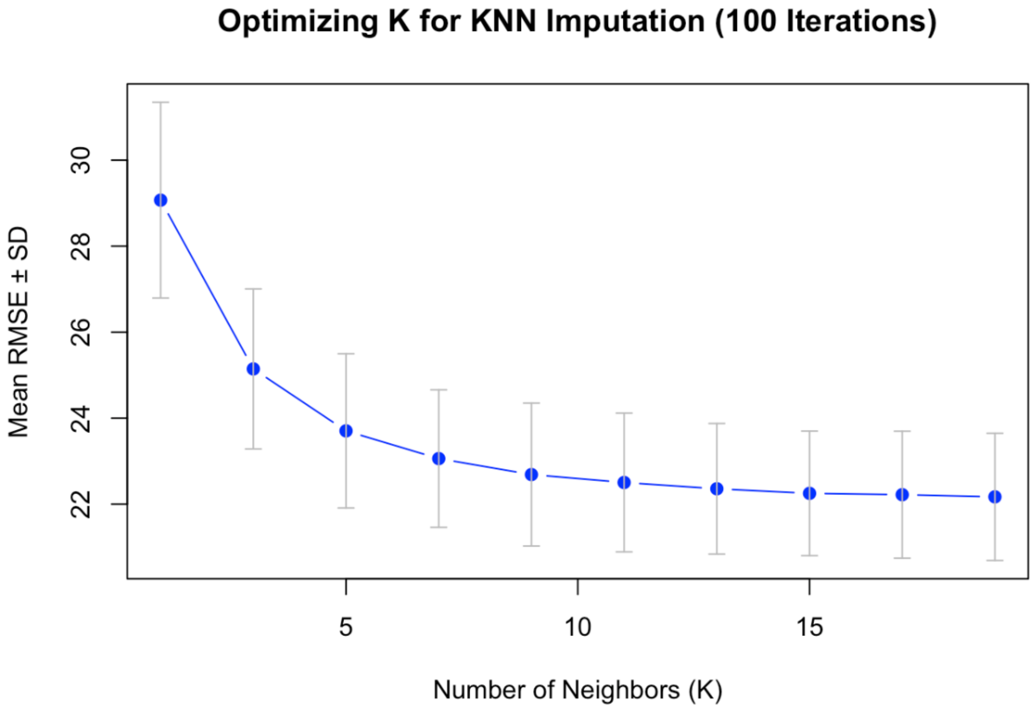

This approach is supported by prior studies using similar methodology where KNN imputation was used with K = 5.[13,14] Imputation was done using the *VIM* packaged in R.

Quantification of viral concentration:

Nucleic acid extraction: Bioaerosol samples were collected between 2015 – 2019 and stored at -80°C until nucleic acid extraction. Viral nucleic acid extraction was optimized through a series of experiments where synthetic SARS-CoV-2 (Seracare Catalog #0505-0126) ranging from 10 to 1000 copies was spiked on to PTFE filters and recovery rates compared based on qPCR targeting the SARS-CoV-2 N1 gene region. These experiments demonstrated that neither mechanical lysis with beadbeating nor addition of carrier RNA improved viral nucleic acid recovery, as well as identified the best approach to elute viral nucleic acids off the PTFE filters. Our viral nucleic extraction procedure utilized a magnetic bead-based approach on the automated Kingfisher flex platform using the Maxwell HT Viral TNA Kit (Promega AX2340). Off-deck sample lysis was first performed by transferring the PTFE filters to 2 mL Eppendorf tubes, adding lysis buffer and proteinase K, incubating the Eppendorf tubes at 10 minutes at 1200 rpm at room temperature, then incubating the tubes for 10 minutes at 1200 rpm at 60°C. The sample lysate was then transferred to deep-well plates on the Kingfisher flex and extraction was continued according to manufacturer instructions.

To increase sensitivity of detection, pre-amplification using real-time polymerase chain reaction (RT-PCR) for 14 cycles was performed (TrueMark Respiratory Panel 2.0 PreAmp Primers, ThermoFisher). No-template negative

controls were included at each step of extraction and pre-amplification. Xeno spike-in (TaqMan, Applied Biosystems) was added during the extraction process to verify amplification efficiency. After amplification, quantitative PCR was used to detect both Xeno as well as human RNAse P (RPPH1) (ThermoFisher) to confirm adequate amplification and adequate bioaerosol collection, respectively.

Digital droplet PCR (ddPCR) Overview:
After pre-amplification, ddPCR was performed with using the following primers and probes:

Probe	Nucleic Acid Type	Manufacturer	Assay ID	Color
Human Coronavirus OC43	RNA	ThermoFisher	Vi06439646_s1	FAM
Human Enterovirus D68	RNA	ThermoFisher	Vi06439669_s1	FAM
Human Parainfluenza virus 2	RNA	ThermoFisher	Vi06439672_s1	FAM
Human Coronavirus HKU1	RNA	ThermoFisher	Vi06439674_s1	FAM
Human Enterovirus (pan assay)	RNA	ThermoFisher	Vi06439631_s1	FAM
TaqManTM Universal RNA Spike In/Reverse Transcription (Xeno) Control		ThermoFisher	Ac00010014_a1	FAM
Human Parainfluenza virus 3	RNA	ThermoFisher	Vi06439670_s1	VIC
Human Parainfluenza virus 1	RNA	ThermoFisher	Vi06439642_s1	VIC
Human RNase P RPPH1 gene		ThermoFisher	Hs04930436_g1	VIC
Human Coronavirus NL63	RNA	ThermoFisher	Vi06439673_s1	VIC
Adenovirus	DNA	ThermoFisher	Vi99990001_po	Cy5
Human Metapneumovirus (hMPV)	RNA	ThermoFisher	Vi99990004_po	Cy5
Human Respiratory Syncytial Virus A (RSV-A)	RNA	ThermoFisher	Vi99990014_po	Cy5
Influenza A	RNA	ThermoFisher	Vi99990011_po	Cy5
Influenza A (H1N1 subtype)	RNA	ThermoFisher	Vi99990009_po	Cy5
Human Parechovirus	RNA	ThermoFisher	Vi99990006_po	Cy5
Human Respiratory Syncytial Virus B (RSV-B)	RNA	ThermoFisher	Vi99990015_po	Cy5.5
Influenza A (H3N2 subtype)	RNA	ThermoFisher	Vi99990010_po	Cy5.5
Influenza B	RNA	ThermoFisher	Vi99990012_po	Cy5.5
Human Parainfluenza virus 4	RNA	ThermoFisher	Vi99990005_po	Cy5.5
Human Rhinovirus 1/2	RNA	ThermoFisher	Vi99990016_po	Cy5.5

Abbreviations: RNA, ribonucleic acid; DNA, deoxyribonucleic acid.

Multiplexed ddPCR assays were designed, optimized, and tested with up to four viral targets per panel using plasmid-based positive controls (TruMark Respiratory Panel 2.0 Amplification Control, ThermoFisher). Negative controls included triplicates of yeast tRNA and poly(A) as well as nucleic acids from field blanks. After droplet generation with the QX200 Auto Droplet Generator and PCR amplification, the QX600 Droplet Reader was used to quantify viral copy number with output analyzed using the QX Manager Software (v2.2, Bio-Rad, Hercules, CA, USA). For quality control, only samples with a minimum of 10,000 droplets generated were analyzed. If samples fail to meet this threshold, repeat testing was done until the minimum droplet threshold was achieved. The limit of detection was calculated using negative controls specific to each multiplexed assay and probe target. Positive droplets were identified using thresholding, with positive detection defined as droplets exceeding the limit of detection.

Multiplexed ddPCR design:

<u>Testing of viral probes:</u> To ensure robust detection of all 19 viral targets of interest, viral probes were first tested individually using quantitative PCR. Each probe was tested with a positive control made up of plasmid viral controls (TrueMark™ Respiratory Panel 2.0 Amplification Control) at 20,000 viral copies/µl and 10,000 viral copies/µl to ensure adequate detection of each viral target of interest. Two different types of controls, yeast tRNA and poly (A) RNA, were used to assess potential off target binding and non-specific amplification. To ensure consistency, yeast tRNA and poly(A) both underwent 14 cycles of preamplification using the same pre-amplification primers as were used for samples (TrueMark Respiratory Panel 2.0 PreAmp Primers (ThermoFisher)).

© 2025 Sun Y et al. *JAMA Network Open*

Design of Multiplexed Assays: Different combinations of viral probes were tested using a standard set of positive and negative controls. Positive controls included serially diluted plasmids that included each viral target of interest with concentration of 100,000, 10,000, 1000, 100, 10, 1, and 0.1 viral copies/µl. The negative controls included eight replicates each of both yeast tRNA and Poly (A), as well as one no-template control. After each multiplexed panel was validated using this method, each panel was then used to test each air sample. Final multiplex assays are summarized below:

Assay	Target 1 (FAM)	Target 2 (VIC)	Target 3 (Cy5)	Target 4 (Cy 5.5)
Panel 1		Parainfluenza 3	Influenza A (H1N1)	
Panel 2	Parainfluenza 2			
Panel 3	Coronavirus (HKU1)	Influenza C	hMPV	
Panel 4	Enterovirus (D68)	Parainfluenza 1	RSV-A	Influenza B
Panel 5	Coronavirus (OC43)		Adenovirus	Parainfluenza 4
Panel 6	Enterovirus	Coronavirus (NL63)	Parechovirus	Influenza A (H3N2)
Panel 7	Rhinovirus			
Panel 8		Enterovirus	RSV-B	
Panel 9	Influenza A		RSV-B	

Abbreviations: ddPCR, digital droplet polymerase chain reaction; hMPV, Human Metapneumovirus; RSV, respiratory syncytial virus

Thresholding: Droplet fluorescence was analyzed using the Bio-Rad QX600 Droplet Reader and the QX Manager (v2.2) software. Thresholds for distinguishing positive and negative droplets were set for each target in each plate based on the fluorescence intensity of positive samples and no template controls (NTCs). Thresholding was performed using automated cutoffs set at 20-40% of the difference between the amplitude of positive samples and NTCs, with manual adjustments applied for droplets exceeding these thresholds.

Calculation of limit of detection (LOD): The LOD was defined as the lowest concentration of target DNA that could be reliably detected in ≥95% of replicate reactions. The threshold for positive detection was set based on the number of positive droplets detected from negative controls (yeast tRNA and Poly(A)) plus three standard deviations (mean + 3SD). Minimum LOD for each target was 3 droplets.

Viral Quantification:

To adjust for effect of preamplification, we used viral plasmid standards with starting concentrations of 0.1, 1, 10, 100, and 1000 copies/µl. These standards underwent preamplification for 4x, 8x, 14x, and 20x cycles using the same preamp primers used for our air samples (TrueMark Respiratory Panel 2.0 PreAmp Primers (ThermoFisher)). After this, ddPCR analysis was performed using the same multiplex assays as detailed above in order to calculate copies/µl (after preamplification). Linear regression modeling was then conducted to estimate the starting copies/µl (before preamplification). The model, shown below, included $\log_2$-transformed copies/µl (after preamplification), number of preamplification cycles, and an interaction term between these two predictors as variables:

$$Y = \beta_0 + \beta_1 X + \beta_2 C + \beta_3 (X \times C) + \epsilon$$

Here, Y = log2-transformed copies/µl (before preamplification), X = log2-transformed copies/µl (after preamplification), C = number of preamplification cycles, $X \times C$ = interaction term between post-preamplification copies and cycle number, β_0 = intercept, $\beta_1, \beta_2, \beta_3$ = regression coefficients, ϵ = error term. This approach achieved an R^2 of 0.73 and was used to estimate copies/µl (before preamplification) for each air filter sample. The estimated viral copies/µl (before preamplification) were then normalized to the volume of air filtered during the week-long sampling period to calculate viral copies per cubic meter (copies/m^3) of air filtered. All analyses were performed using R.

© 2025 Sun Y et al. *JAMA Network Open*

K-means clustering of individual viral concentrations into exposure groups:

K-means clustering was used to group samples based on viral concentration (copies/m^3). Viral concentrations were first normalized using Z-score transformation to allow adequate comparisons between different viruses. The optimal number of clusters was determined by applying the elbow method, which evaluates the within-cluster sum of squares, and the silhouette method to assess adequate cluster separation. Both methods indicated that the optimal number of clusters was two. Principal component analysis (PCA) showed clear separation of viral clusters **(eFigure 2 in Supplement 2)**. Based on this, samples are binarized using the *cluster()* function in R into two distinct clusters, with cluster 1 showing overall higher concentration of viruses **(eFigure 5 in Supplement 2)**. A summary of viral concentrations in each cluster are shown in **eTable 3 in Supplement 2**. Given that cluster 1 had overall higher viral concentrations, this cluster was used as a marker of high global viral exposure, which was our primary outcome.

Statistical Modeling:

Primary intention to treat analysis:

1) **Primary Outcome:**

The primary outcome of high viral exposure (binary) was analyzed using a generalized linear mixed effects model (GLMM) with a logit link:

$$\text{logit}\left(P\left(Y_{ijk}=1\right)\right) = \beta_0 + \beta_1 * \text{hepa}_j + \beta_2 * \text{post\_hepa}_i + \beta_3 * \left(\text{hepa}_j * \text{post\_hepa}_i\right) + \upsilon_k + \omega_{k(j)}$$

Here, Y_{ijk} is the primary outcome (high viral exposure) for i^{th} sample in class j in school k. This model accounts for fixed effect for HEPA intervention group (hepa_j, where 1 indicates HEPA-intervention group and 0 indicates sham group) and the intervention period (post_hepa_i, where 1 indicates post-HEPA intervention, and 0 indicates pre-HEPA intervention). The primary effect of interest is the interaction term $\text{hepa}_j * \text{post\_hepa}_i$, representing the differential effect of HEPA over time. Random intercepts were included for schools (υ_k) and classrooms nested within schools ($\omega_{k(j)}$), to account for both classroom- and school-level clustering as well as repeated measurements within classrooms over time.

2) **Secondary outcomes:**

The secondary outcome of viral diversity (continuous) and individual viral concentrations (continuous), all models were linear mixed-effects models (LMMs) with the same random effect structure:

$$Y_{ijk} = \beta_0 + \beta_1 * \text{hepa}_j + \beta_2 * \text{post\_hepa}_i + \beta_3 * \left(\text{hepa}_j * \text{post\_hepa}_i\right) + \upsilon_k + \omega_{k(j)} + \varepsilon_{ijk}$$

Here, Y_{ijk} is the primary outcome (viral diversity or individual viral concentrations). We Z-score transformed viral concentrations in order to allow for better comparability between different viruses. Similarly to our GLMM model, we include fixed effects for HEPA intervention group (hepa_j) and intervention period (post_hepa_i), with the interaction term ($\text{hepa}_j * \text{post\_hepa}_i$) our primary effect of interest.

Sensitivity Analysis:

To adjust for seasonality, we chose to utilize a generalized additive mixed effect model (GAMM), which allows flexible modeling of non-linear relationships. A spline term from the days from the start of school ($\text{days since school start}_\text{i}$) in order to model season. Fixed and random effects are maintained from our GLMM model. For the primary outcome of high viral exposure, a GAMM with binomial distribution and logit link was used:

$$\begin{aligned}\text{logit}\left(P\left(Y_{ijk}=1\right)\right) = \\ &= \beta_0 + \beta_1 * \text{hepa}_j + \beta_2 * \text{post}_{\text{hepa}_i} + \beta_3 * \left(\text{hepa}_j * \text{post}_{\text{hepa}_i}\right) \\ &+ f(\text{days since school start}_\text{i}) + \upsilon_k + \omega_{k(j)}\end{aligned}$$

© 2025 Sun Y et al. *JAMA Network Open*

For our continuous secondary outcomes, GAMMs with gaussian distributions were applied:

$$Y_{ijk} = \beta_0 + \beta_1 * \text{hepa}_j + \beta_2 * \text{post}_{\text{hepa}_i} + \beta_3 * \left(\text{hepa}_j * \text{post}_{\text{hepa}_i}\right) +$$

$$f(\text{day since school start}_\text{i}) + \upsilon_k + \omega_{k(j)} + \varepsilon_{ijk}$$

In both models, fixed and random effect structures specified from the GLMMs and LMMs were used.

Predictive modeling of high viral exposure:

To investigate which environmental factors are most predictive of high viral exposure, we developed a predictive model using Elastic Net regression. First, all modifiable indoor air quality measures (CO_2, relative humidity, temperature, $PM_{2.5}$, coarse PM, and air exchange rate) were normalized using Z-score transformation. Then, the entire dataset was randomly split 50:50 into testing and validation testsets to ensure equal distribution of our outcome of high viral exposure across datasets. Using the *glmnet* method within the *caret* package in R, we trained our model using 5-fold cross-validation to optimize hyperparameters and prevent overfitting. High viral exposure was modeled as a binary outcome, while predictors included indoor air quality measures, classroom characteristics (central HVAC system, grade, classroom dimensions, and class size), seasonality (modeled as a binary variable for winter), and HEPA-intervention (modeled as a binary variable based on whether HEPA intervention was implemented).

Elastic Net Model: Hyperparameter tuning was first performed to optimize the alpha mixing parameter to balance L1 (Lasso) and L2 (Ridge) regularization, allowing for both feature selection and shrinkage. We further optimized our model using 50 different values for the regularization parameter (lambda). The model was trained on the training dataset using 5-fold cross validation, repeated 5 times. Tuning was done to maximize area under the receiver operating characteristic curve (AUC), ensuring the best predictive performance **(eFigure 7A in Supplement 2).** The final model was then fitted onto the validation dataset to assess model performance **(eFigure 7B in Supplement 2)**. Each predictor was ranked based on feature importance, and coefficients were extracted from model derived using the validation set in order to calculate ORs associated with each feature.

Bootstrapping: To assess the reliability of our model estimates, we then employed bootstrapping to find confidence intervals for the model coefficients obtained using the validation dataset. Specifically, we performed 2,000 bootstrap iterations, fitting the final Elastic Net model on each resampled dataset. From each resampled iteration, we derived 95% CIs for the model coefficients, which were then transformed into ORs for enhanced interpretability. Model coefficients and 95% CIs are shown in **Figure 4**, with features arranged from highest OR (top) to lowest OR (bottom). Variables where the 95% does not cross 1 are highlighted in yellow.

Random Forest: Lastly, to ensure that predictive modeling results were robust across machine learning methods, we used Random Forest as a second approach. Similar to Elastic Net, all continuous indoor air quality and classroom factors were Z-score transformed prior to analysis, and the dataset was split 50:50 into training and validation test sets. Hyperparameter tuning for optimal *mtry* (number of variables evaluated at each split) and *ntree* (number of trees) were done using 5-fold cross validation repeated five times in order to maximize AUC. Final AUC for the training model was 0.80. When this model was tested on the validation dataset, AUC was 0.80 indicating good generalizability. Features ranked based on importance are shown **(eFigure 8B in Supplement 2)**. Analysis was performed using *randomForest* package in R.

Post-hoc power analysis: We conducted a post-hoc power analysis based on estimated parameters of in our primary GLMM model with high viral exposure as our primary outcome, including all the coefficients, and the random variances (intraclass correlation coefficients). The estimated power (assuming constant intraclass correlation coefficients) to detect each odds ratio was as follows:

**Odds Ratio**	**Power (%)**
0.5	37.6

© 2025 Sun Y et al. *JAMA Network Open*

0.45	53.1
0.4	68.1
0.36	80.0
0.33	90.0

These results suggest that our study was well powered to detect large effect sizes (OR <= 0.36) but had limited power for more modest reductions in overall viral exposure.

eReferences

1. Lee WC, Catalano PJ, Yoo JY, Park CJ, Koutrakis P. Validation and Application of the Mass Balance Model To Determine the Effectiveness of Portable Air Purifiers in Removing Ultrafine and Submicrometer Particles in an Apartment. *Environ Sci Technol*. 2015;49(16):9592-9599. doi:10.1021/acs.est.5b03126

2. Burton NC, Grinshpun SA, Reponen T. Physical collection efficiency of filter materials for bacteria and viruses. *Ann Occup Hyg*. 2007;51(2):143-151. doi:10.1093/annhyg/mel073

3. Soo JC, Monaghan K, Lee T, Kashon M, Harper M. Air sampling filtration media: Collection efficiency for respirable size-selective sampling. *Aerosol Sci Technol*. 2016;50(1):76-87. doi:10.1080/02786826.2015.1128525

4. Demokritou P, Gupta T, Ferguson S, Koutrakis P. Development and laboratory performance evaluation of a personal cascade impactor. *J Air Waste Manag Assoc*. 2002;52(10):1230-1237. doi:10.1080/10473289.2002.10470855

5. Batterman S. Review and Extension of CO_2-Based Methods to Determine Ventilation Rates with Application to School Classrooms. *Int J Environ Res Public Health*. 2017;14(2):145. doi:10.3390/ijerph14020145

6. Ekberg LE, Kraenzmer M. Determination of Ventilation Rates by CO2 Monitoring: Assessment of Inaccuracies. In: Proceedings of the 17th AIVC Con ference,. AIVC; 1998:461-468.

7. Zand MS, Spallina S, Ross A, et al. Ventilation during COVID-19 in a school for students with intellectual and developmental disabilities (IDD). *PLoS One*. 2024;19(4):e0291840. doi:10.1371/journal.pone.0291840

8. Ventilation Mitigation Strategies. Department of Health and Human Services, Centers for Disease Control and Prevention, National Institute for Occupational Safety and Health. Accessed July 17, 2025. https://www.cdc.gov/niosh/ventilation/prevention/index.html

9. Phipatanakul W, Koutrakis P, Coull BA, et al. Effect of School Integrated Pest Management or Classroom Air Filter Purifiers on Asthma Symptoms in Students With Active Asthma: A Randomized Clinical Trial. *JAMA*. 2021;326(9):839-850. doi:10.1001/jama.2021.11559

© 2025 Sun Y et al. *JAMA Network Open*
