# Supplementary material for: Air Purifier Intervention for Respiratory Viral Exposure in Elementary Schools: A Secondary Analysis of a Randomized Clinical Trial
Source: JAMA Netw Open. 2025 Oct 10;8(10):e2536951. doi: 10.1001/jamanetworkopen.2025.36951 (PMC12514627; doi:10.1001/jamanetworkopen.2025.36951)
Supplement: Supplement 3. — Data Sharing Statement [file jamanetwopen-e2536951-s003.pdf]

Data Sharing Statement

Sun. Air Purifier Intervention for Respiratory Viral Exposure in Elementary Schools. *JAMA Netw Open*. Published October 10, 2025. doi:10.1001/jamanetworkopen.2025.36951

Data
Additional Information: ClinicalTrials.gov identifier: NCT02291302
Data available: Yes
Data types: Data (not involving human participants), Data dictionary
How to access data: plai@mgh.harvard.edu
When available: With publication

Supporting Documents
Document types: Statistical/analytic code
How to access documents: Data analysis code and analytical plan can be accessed at: https://osf.io/nku9b/?view_only=eb9773e974184778b470254970d95511
When available: With publication

Additional Information
Who can access the data: researchers whose proposed use of the data has been approved
Types of analyses: For approved purpose
Mechanisms of data availability: After approval of a proposal with signed data access agreement and relevant regulatory approvals
